# Supplementary material for: Mouse frontal cortex mediates additive multisensory decisions
Source: Neuron. 2023 Aug 2;111(15):2432–2447.e13. doi: 10.1016/j.neuron.2023.05.008 (PMC10957398; doi:10.1016/j.neuron.2023.05.008)
Supplement: Document S1. Figures S1–S8 [file mmc1.pdf]

Neuron, Volume *111*

Supplemental information

Mouse frontal cortex mediates additive multisensory decisions

Philip Coen, Timothy P.H. Sit, Miles J. Wells, Matteo Carandini, and Kenneth D. Harris

Neuron, Volume *111*

Supplemental information

Mouse frontal cortex mediates additive multisensory decisions

Philip Coen, Timothy P.H. Sit, Miles J. Wells, Matteo Carandini, and Kenneth D. Harris

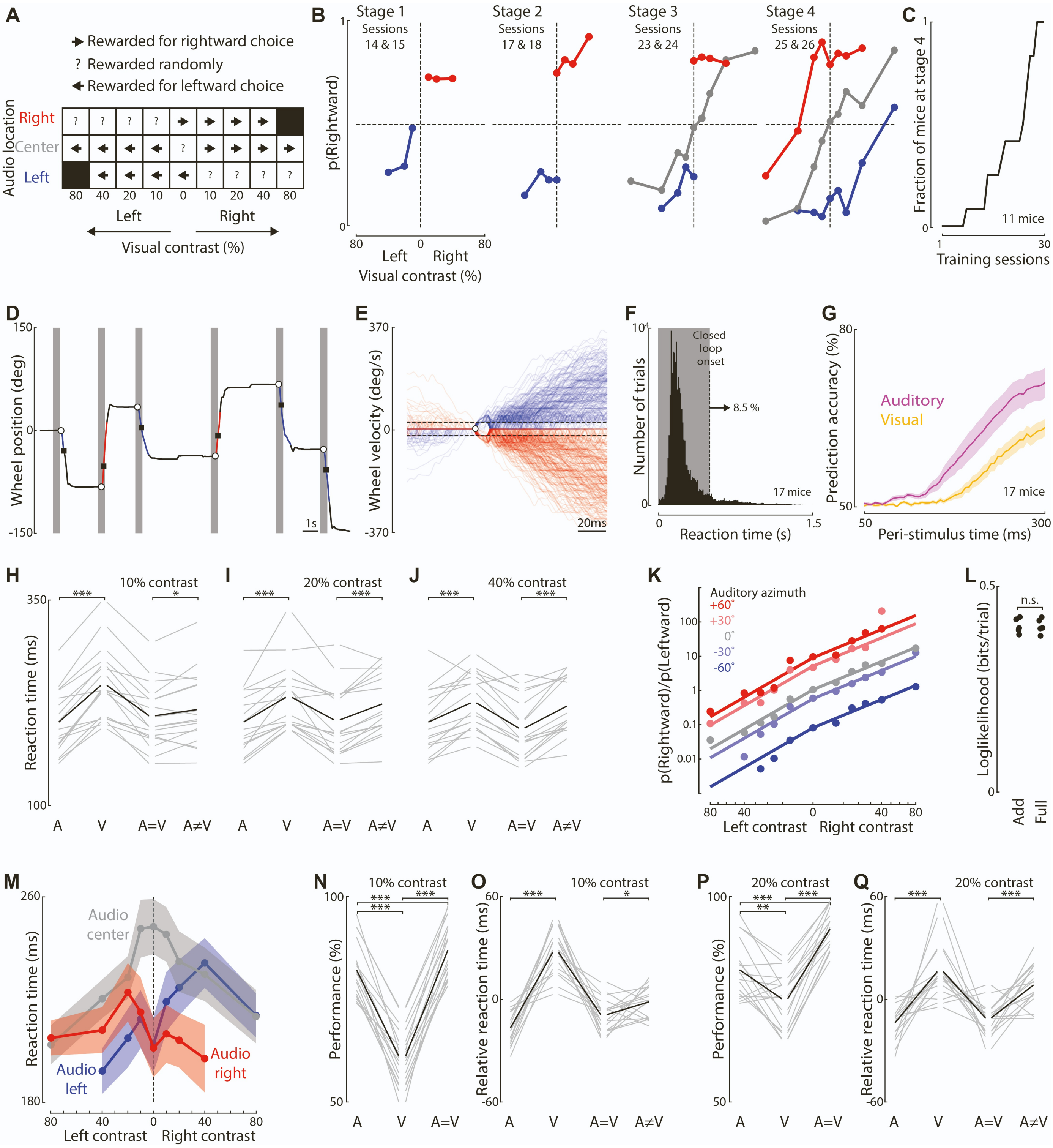

Figure S1 Training and behavioral classification, related to Figure 1

(A) *Matrix of stimulus conditions and their corresponding reward criteria.* ***(B)*** *Training progression for one mouse. Mice first reach proficiency with audiovisual coherent trials (Stage 1), before we introduce unisensory auditory (Stage 2), unisensory visual (stage 3) and conflict (Stage 4) stimulus types. Plots for stages 1, 2, and 3, were made from the final two sessions of these stages. For stage 4, data is taken from the mouse's first two sessions at that stage.* ***(C)*** *Number of sessions required to train mice. In our final training pipeline, ~80% of mice learned the task. Those that did learn required less than 30 sessions (median: 24.5).* ***(D)*** *Sample of wheel position trace. Grey regions indicate the 500ms open-loop period, beginning with stimulus onset. Open circles and squares indicate the movement onset and decision threshold (see STAR Methods).* ***(E)*** *Zoom in to wheel velocity traces around the time of movement onset (open circle), for the same behavioral session as (D). Red and blue lines indicate leftward and rightward choices. Dashed lines represent the velocity thresholds for movement onset (see STAR Methods).* ***(F)*** *Histogram of reaction times across 17 mice (~156K trials). Shaded region indicates the 500ms open-loop period, during which 91.5% of movements were initiated.* ***(G)*** *Accuracy of predicting whether a stimulus was presented on the left or right side from wheel velocity at each time point relative to stimulus onset, using a threshold obtained by minimizing an SVM loss function, for unisensory visual (yellow) or unisensory auditory (magenta) trials. Shaded areas indicate the standard error across 17 mice. Earlier predictions on auditory trials confirm that mice can decode the location of auditory stimuli earlier than visual stimuli (i.e. earlier auditory reaction times do not reflect guesses).* ***(H)*** *Comparing median reaction times for each stimulus type for each mouse, for trials with 10% visual contrast. Grey and black lines indicate individual mice and the mean across mice. ***: p*

< 0.001, $*$: $p < 0.05$ (17 mice, paired t-test); only comparisons between auditory and visual unisensory, and between conflict and coherent are shown. ***(I)*** *As in (H), but for trials with 20% visual contrast.* ***(J)*** *As in (H), but for trials with 40% visual contrast.* ***(K)*** *Fit of the additive model to a mouse presented with 5 different auditory locations and 11 different contrast levels. Two additional parameters were used to fit the additional auditory stimuli (see STAR Methods).* ***(L)*** *5-fold cross-validated estimates of the loglikelihood for the additive and full (a parameter for each stimulus combination) behavioral models relative to a bias-only model. n.s.* $p > 0.05$ ***(M)*** *The mean (across 17 mice) of the median reaction times for each stimulus condition (∼156K trials). Shading: standard error across mice.* ***(N)*** *Mouse performance (% rewarded trials) for unisensory auditory, unisensory visual, and coherent multisensory stimulus conditions (correct performance on conflict trials is undefined). This panel shows only trials with 10% visual contrast. Grey and black lines indicate individual mice and the mean across mice.* $***$: $p < 0.001$ *(17 mice, paired t-test).* ***(O)*** *As in (H), but for reaction times relative to the mouse's mean reaction time across all stimulus types.* ***(P)*** *As in (N), but for trials with 20% visual contrast.* $***$: $p < 0.001$ *(17 mice, paired t-test). Data with 40% contrast is shown in Figure 1E.* ***(Q)*** *As in (I), but with reaction times relative to the mouse's mean reaction time across all stimulus types. Data with 40% contrast is shown in Figure 1B.*

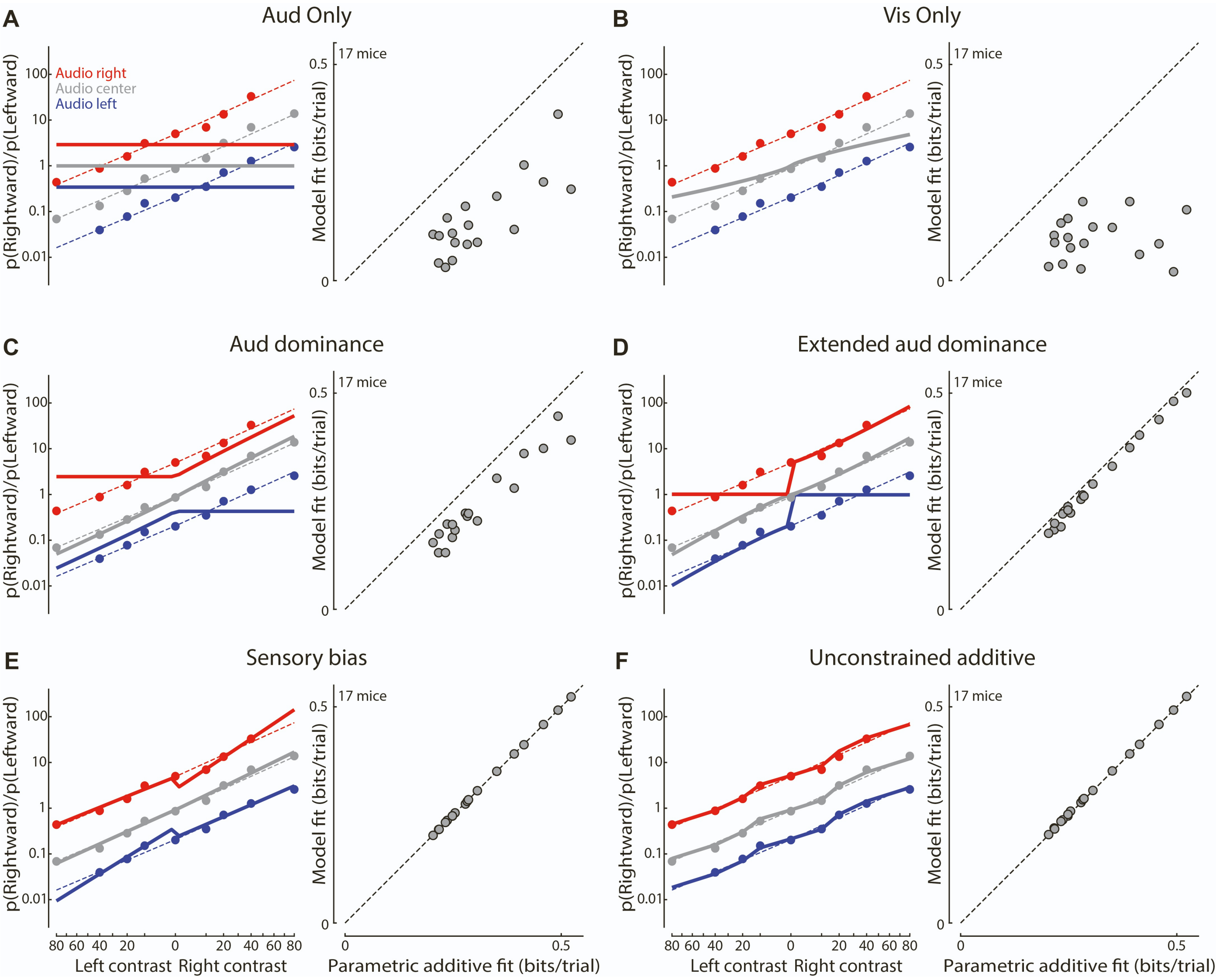

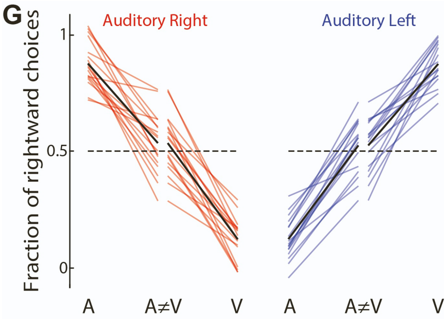

Figure S2 No evidence for sensory bias in behavior, related to Figure 1

***(A) Left**: Fit of an auditory-only model where both visual sensitivities (v_R and v_L) are set to 0. Data plotted as the odds of choosing right vs. left (in log coordinates, Y-axis), as a function of visual contrast raised to the power γ. Closed circles: data combined across 17 mice. Red, grey, and blue curves: combined fit across all mice (17 mice ~156K trials). Dashed lines: fit of the additive model to the same data. The x-axis is visual contrast raised to the power γ from the additive model. **Right**: $\mathrm{Log_2}$-likelihood ratio for the auditory-only model versus the parametric additive model (both models assessed by 5-fold cross-validation relative to a bias-only model). The parametric additive model is a significantly better fit ($p < 0.001$, paired t-test). **(B)** As in (A), but for a visual-only model where both auditory sensitivities (a_R and a_L) are set to zero. The parametric additive model is a significantly better fit ($p < 0.001$). **(C)** As in (A), but for a modified model that incorporates auditory dominance. Here, both visual sensitivities (v_R and v_L) are set to zero on audiovisual conflict trials. The parametric additive model is a significantly better fit ($p < 0.001$). **(D)** As in (A) but for an extended auditory dominance model with an additional parameter to allow for a change in auditory sensitivity on conflict trials (see STAR Methods). The parametric additive model is a significantly better fit ($p < 0.001$). **(E)** As in (A), but for a general sensory bias model with 4 additional parameters to allow for a change in visual or auditory sensitivity on conflict or coherent trials. The additive model is a subset of this model (see STAR Methods). There is no significant difference in fit quality between this model and the parametric additive model ($p > 0.05$). **(F)** As in (A), but for the unconstrained additive model with a parameter for each auditory and visual stimulus condition. The superiority of the additive unconstrained model can be seen for example at visual contrast of +10, where the probability of a rightward choice is slightly below the parametric prediction for all three auditory conditions. The unconstrained additive model is a significantly better fit than the parametric additive model ($p < 0.01$). **(G)** Fraction of rightward choices for auditory, visual, and conflict stimulus types at the "matched" visual contrast for each mouse. Red or blue indicate stimulus conditions where the auditory stimulus was on the right or left. The "matched" contrast is the contrast that produces ~(1-X) fraction of rightward choices, where X is the fraction of rightward choices in the paired auditory stimulus condition. For both left and right auditory stimuli, the fraction of rightward choices on conflict trials is not significantly different from 0.5 ($p > 0.05$, t-test, $n = 17$ mice).*

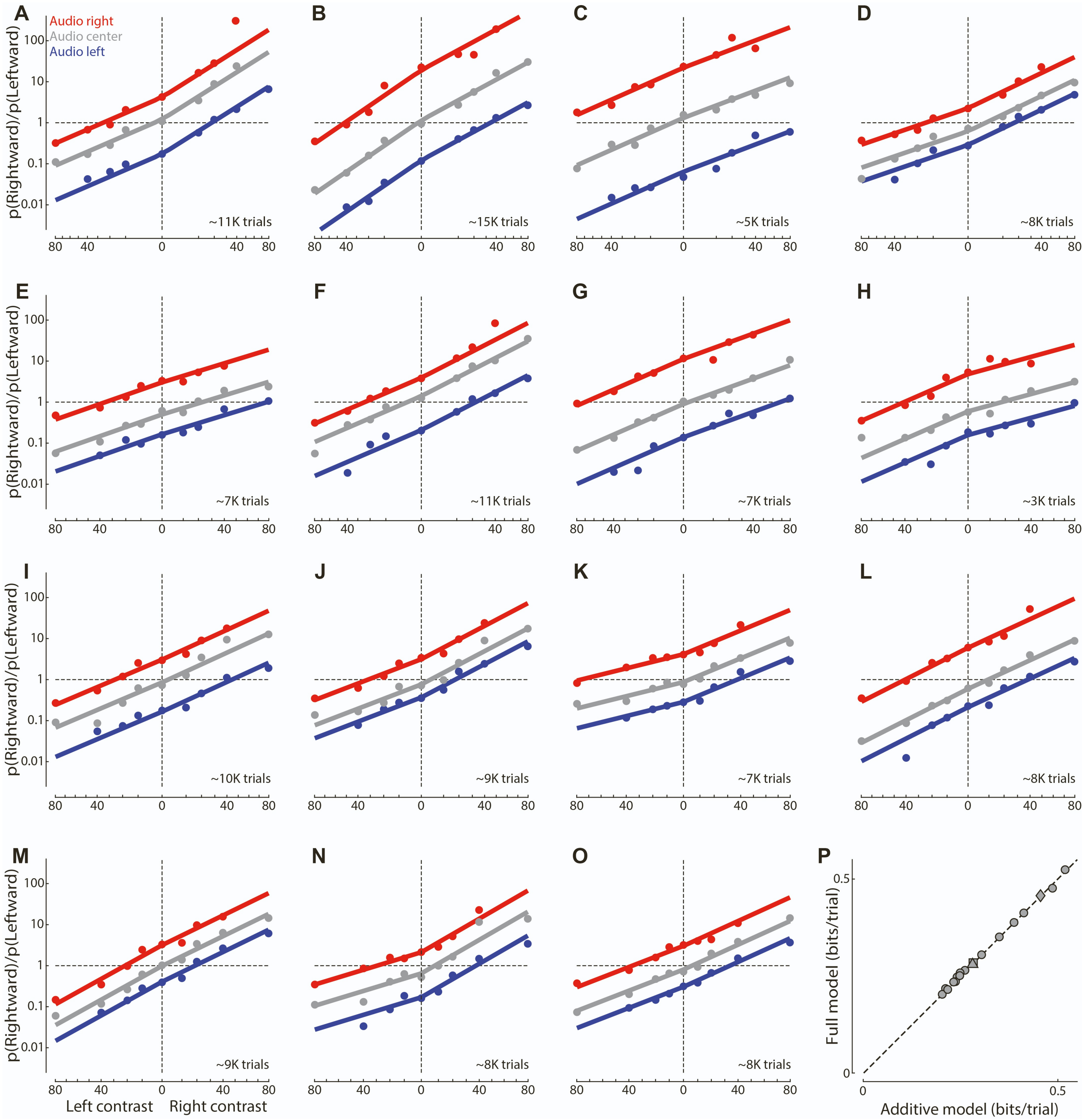

Figure S3 Additive model performance for each mouse, related to Figure 1

(A-O) *Fits of the additive model to all 15 mice additional to those shown in Figure 1C, with different auditory and visual proficiencies, plotted as in Figure 1F.* ***(P)*** *Cross-validated loglikelihood ratio (relative to a bias-only model) for the full model versus the additive model trained on only unisensory stimulus conditions, plotted as in Figure 1H. There is no significant difference between models. p > 0.05 (17 mice, paired t-test).*

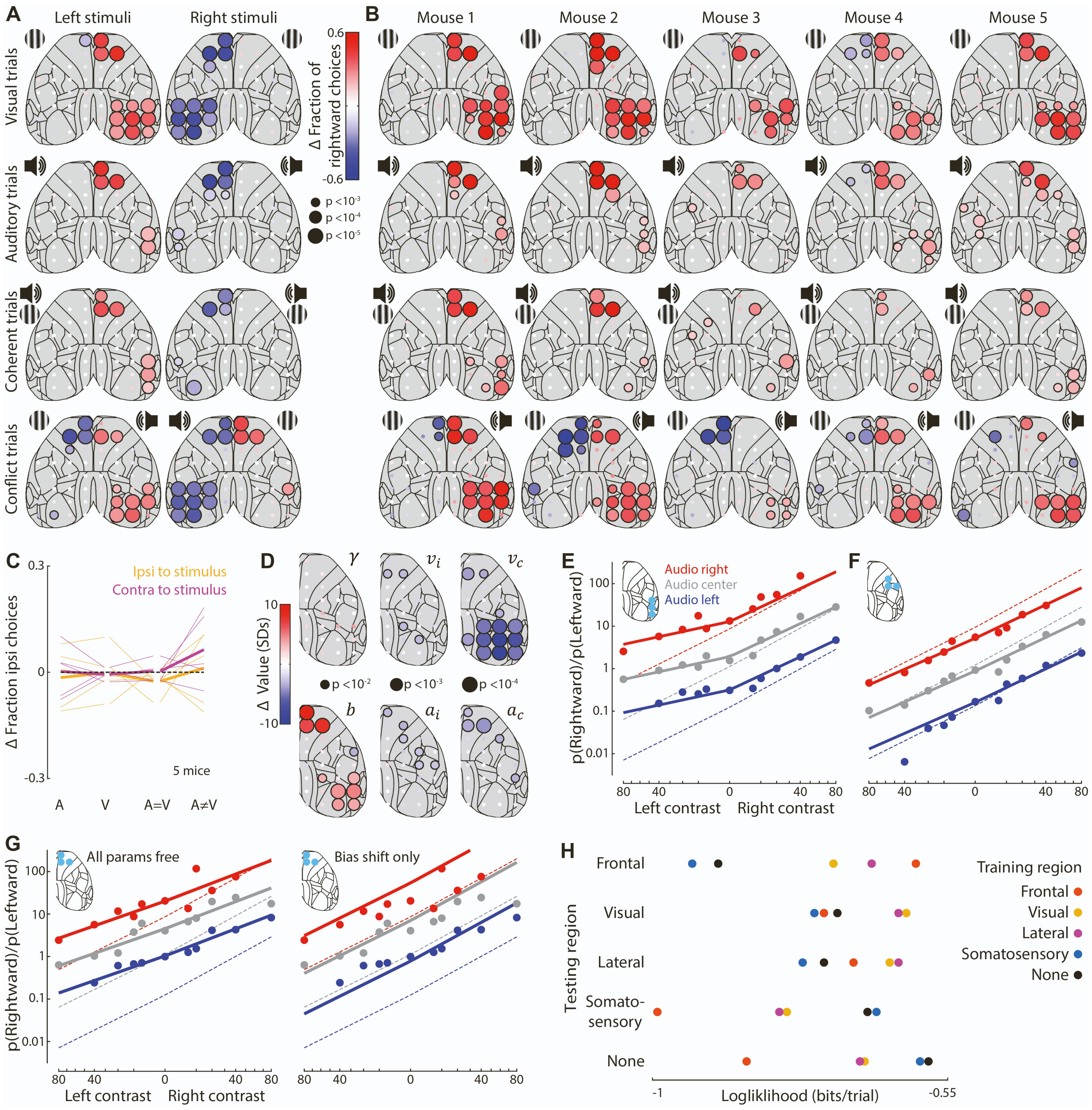

Figure S4 Further analysis of optogenetic inactivation effects on choice, model, and brain regions, related to Figure 2

(A) *Change in the fraction of rightward choices for 52 stimulation sites for unisensory visual, unisensory auditory, coherent, and conflict stimulus conditions. The position of the grating and loudspeaker symbol indicate the locations of the visual and auditory stimuli in each case; Left and right columns represent mirrored cases of the same stimulus condition. Dot color indicates change in the fraction of rightward choices and dot size represents statistical significance (5 mice, shuffle test, see STAR Methods).* ***(B)*** *As in (A), but each column represents an individual mouse, and mirrored trials were combined, reversing if necessary, so that the stimulus (visual in conflict trials) was always on the left. Dot color indicates change in the fraction of rightward choices.* ***(C)*** *Change in the fraction of ipsilateral choices on trials when inactivation target was outside the brain (on the dental cement and/or skin of the mouse). No significant effect was observed for any stimulus condition for ipsilateral (gold) or contralateral (magenta) inactivation sites. $p > 0.05$ (paired t-test).* ***(D)*** *As in main text Figure 2F, but also allowing the contrast gain saturation parameter γ to be fit. No significant changes in the contrast gain parameter (γ) were observed (5 mice, shuffle test, see STAR Methods).* ***(E)*** *Fit of additive model to trials in which any of 3 sites in lateral sensory cortex were inactivated (5 mice, 6600 trials), plotted as in Figure 2G-H. Trials with inactivation of left lateral sensory cortex were included in the average after reflection. The deviation in model parameters was significant (paired t-test, $p < 0.05$).* ***(F)*** *As in (E), but for trials when somatosensory cortex was inactivated (5 mice, 6689 trials). The deviation in model parameters was significant ($p < 0.05$).* ***(G)*** *Fit of the additive model to right frontal cortex inactivation trials (5 mice, 5612 trials), with all parameters free (left, as in Figure 2H, γ was fixed) or with only the bias parameter free to change from its non-inactivation value (right). This "Bias shift only" approach resulted in a worse fit (paired t-test, $p < 0.05$), due to its inability to model changes in sensory sensitivity.* ***(H)*** *Loglikelihood for trials when each testing region was inactivated, evaluated using model parameters from a fit to trials in which the training region was inactivated. In each case, loglikelihood is highest when testing and training regions are the same. Every inter-region loglikelihood is significantly worse ($p < 0.05$, shuffle test, Bonferroni-corrected, see STAR Methods).*

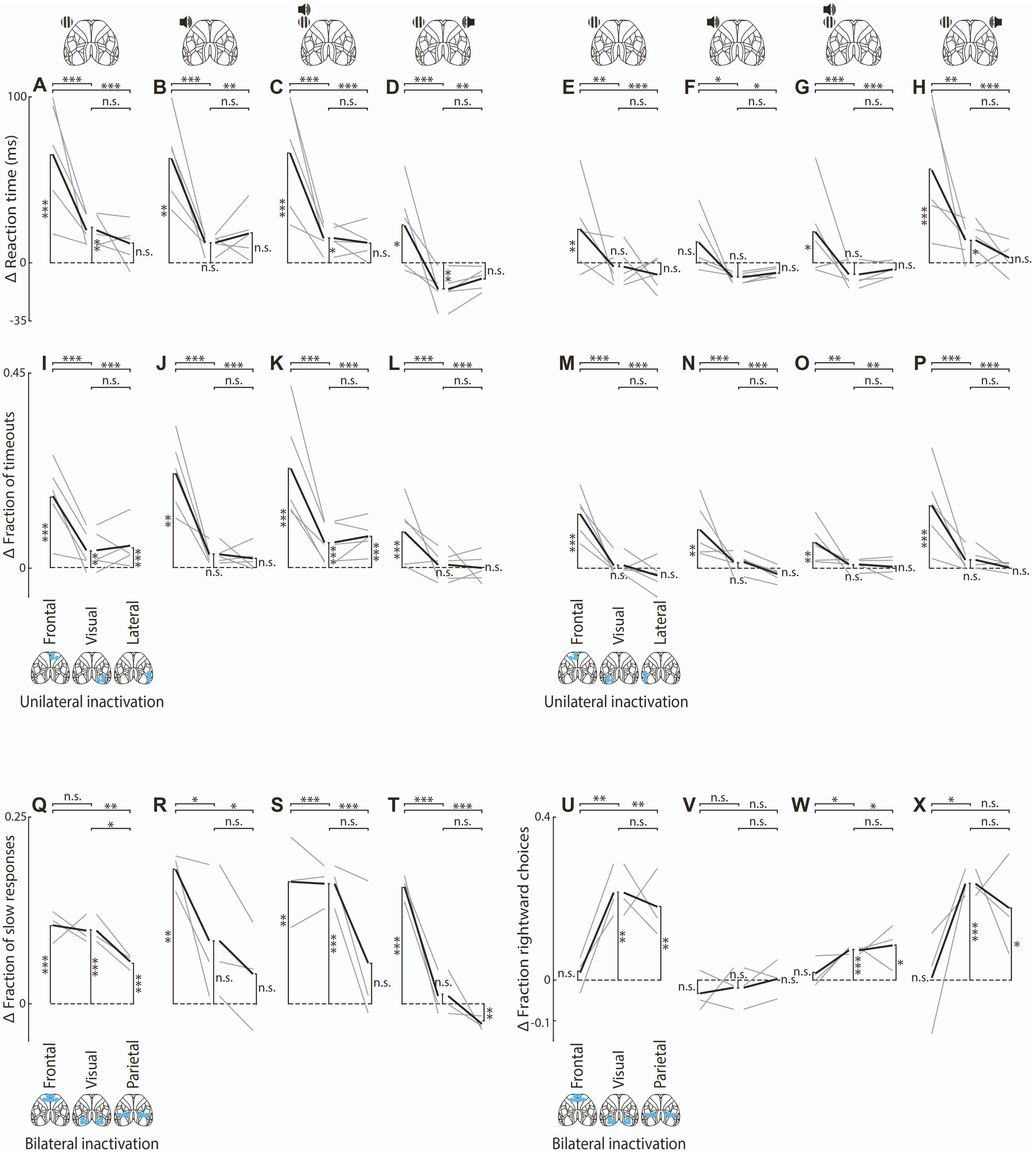

Figure S5 Further effects of uni- and bilateral inactivation on behavior, related to Figure 2

(A) Change in reaction times when frontal, visual, and lateral sensory cortex were inactivated (left, center and right) on visual trials, relative to non-inactivated trials with the same (contrast-matched) stimuli. Inactivated regions could be on the left or right, but were always contralateral to the visual stimulus. Grey and black lines indicate individual mice (n = 5) and the mean across mice. The values for each mouse were calculated by first computing the median reaction time for each contrast, then taking the mean of across contrasts. Values above 100 ms were truncated to 100 ms for visualization but not analyses. n.s: p > 0.05, ∗: p < 0.05, ∗∗: p < 0.01, ∗∗∗: p < 0.001 (linear mixed effects model). ***(B)*** *As in (A) but for auditory trials.* ***(C)*** *As in (A) but for coherent trials.* ***(D)*** *As in (A), but for conflict trials where the visual stimulus was contralateral to the inactivated region.* ***(E-H)*** *As in (A-D) but for trials of reversed laterality such that the stimulus (visual in the case of conflict trials) was ipsilateral to the inactivated region.* ***(I-P)*** *As in (A-H) but for the change in the fraction of timeout trials (no response within 1.5s) rather than reaction times.* ***(Q-T)*** *As in (A-D) but for bilateral inactivation of frontal, visual, and parietal cortices (n = 3 mice). Here, a "slow response" is defined as any response time longer than 300 ms. We used this binarized metric to increase statistical power in view of the limited number of trials in the bilateral, compared to the unilateral, inactivation experiments.* ***(U-X)*** *As in (Q-T) but for the change in the fraction of rightward choices.*

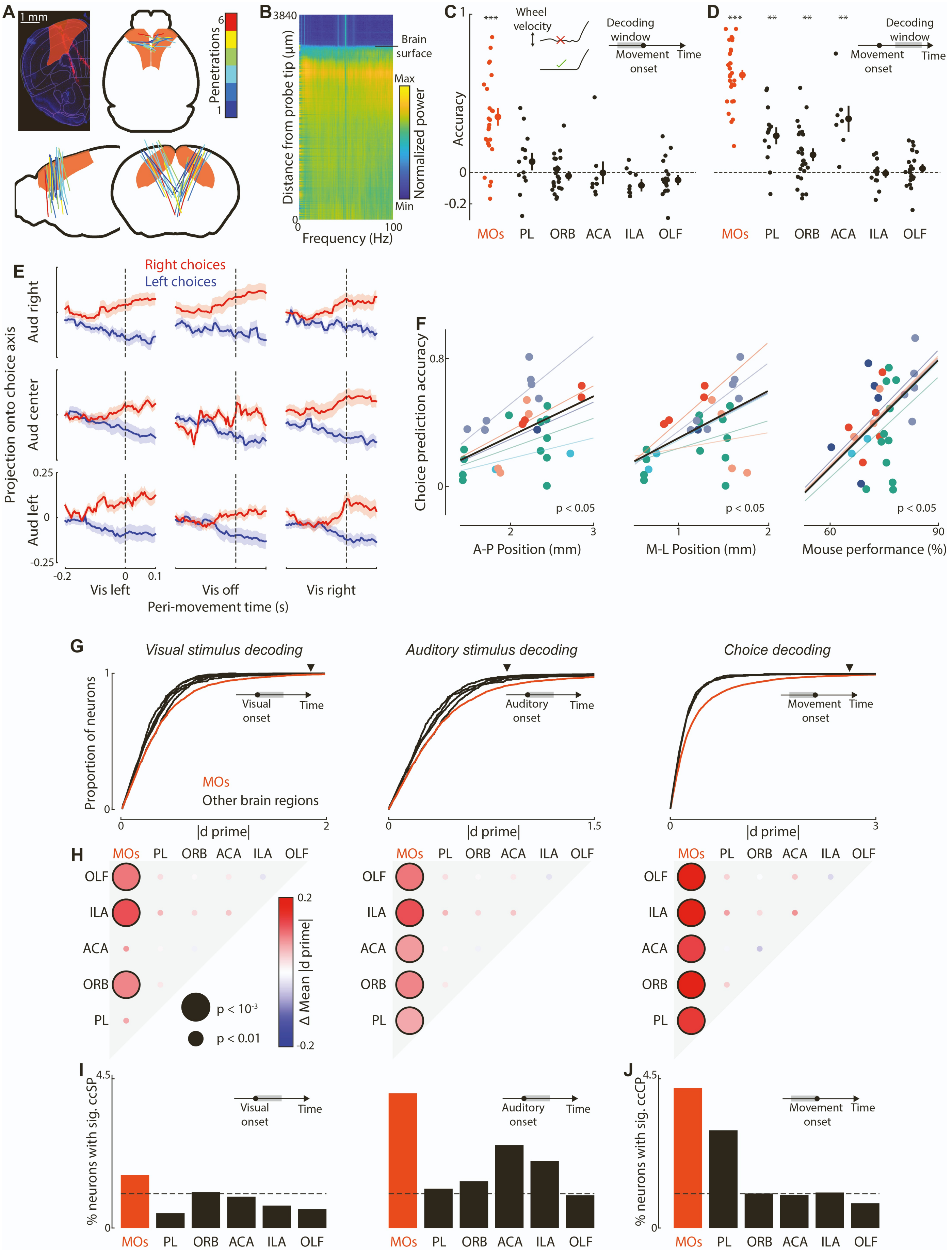

A
1 mm
Penetrations
6
1
B
3840
Distance from probe tip (μm)
Brain surface
Max
Normalized power
Min
0
0
100
Frequency (Hz)
C
1
Accuracy
0
-0.2
***
Wheel velocity
Decoding window
Movement onset
Time
MOs
PL
ORB
ACA
ILA
OLF
D
***
**
**
**
Decoding window
Movement onset
Time
MOs
PL
ORB
ACA
ILA
OLF
E
Right choices
Left choices
Projection onto choice axis
Aud right
Aud center
Aud left
0.25
0
-0.25
-0.2
0
0.1
Vis left
Vis off
Vis right
Peri-movement time (s)
F
Choice prediction accuracy
0.8
0
p < 0.05
A-P Position (mm)
p < 0.05
M-L Position (mm)
p < 0.05
60
90
Mouse performance (%)
G
Visual stimulus decoding
Auditory stimulus decoding
Choice decoding
Proportion of neurons
1
0
Visual onset
Time
MOs
Other brain regions
|d prime|
2
Auditory onset
1.5
Movement onset
3
H
MOs
PL
ORB
ACA
ILA
OLF
0.2
Δ Mean |d prime|
-0.2
p < 10^-3
p < 0.01
I
% neurons with sig. ccSP
4.5
0
J
% neurons with sig. ccCP

Figure S6 Electrophysiology methods and controls, related to Figure 3

(A) *Histological reconstruction of electrode tracks. Top left: Example coronal section (DAPI-staining, blue) at 1.86mm anterior to bregma with tracks from 2 insertions. Slices were registered to the Allen Atlas (Wang et al., 2020) (overlaid, with MOs highlighted in orange) using custom software (see STAR Methods). Right and bottom: reconstructed trajectories of all recordings analyzed, projected onto coronal, sagittal, and horizontal planes. Color indicates the number of penetrations along a given trajectory (one DiI-labelled penetration, same angles and point of insertion).* ***(B)*** *Normalized power spectrum (log(power), z-scored across probe depth) versus depth along probe for a single penetration. The point where the probe enters the brain can be identified by a sudden increase in power.* ***(C)*** *Cross-validated accuracy (relative to a bias model, see STAR Methods) of an SVM decoder trained to predict rightward or leftward choices based on population spiking activity time-averaged over a time window 0 ms to 130 ms preceding movement. Trials where mice made sub-threshold movements prior to movement onset were excluded (illustrated in inset). Each point represents the decoding accuracy from neurons in one brain region (Secondary motor (MOs), orbitofrontal (ORB), anterior cingulate (ACA), prelimbic (PL), infralimbic (ILA), or Olfactory (OLF)), from a single experimental session. ∗∗∗: $p < 0.001$ (≥ 5 sessions from 2-5 mice for each region, one-sided t-test).* ***(D)*** *As in (C), but with a time window of 150 ms to 300 ms after movement onset (see STAR Methods). Trials where mice made sub-threshold movements were not excluded. ∗∗: $p < 0.01$, ∗∗∗: $p < 0.001$.* ***(E)*** *For each stimulus condition, the projection of the population vector at time relative to movement onset to the choice axis (see STAR Methods), with red indicating rightward choice trials and blue indicating leftward choice trials. Solid lines are the mean projection across trials, with the shaded area indicating +/-1 SEM.* ***(F)*** *The accuracy of decoders trained to decode the upcoming choice of the animal 0 – 130 ms before movement onset as a function of either probe position relative to bregma along the anterior-posterior (A-P) axis (left), medial-lateral (M-L) axis (center), or mouse performance (percentage of correct choices, right). Each dot indicates an experiment session and dots of the same color represent sessions from the same mouse. Faint lines show the line of best fit using a linear mixed effects model with a random effect of mouse identity (intercept and slope), and the solid black line shows the mean of the lines of best fit. We observed a significant effect of A-P/M-L probe location, and mouse performance ($p < 0.05$, $n = 5$ mice, linear mixed effects model). There was no significant effect of probe location on stimulus decoding during passive stimulus presentation (data not shown).* ***(G)*** *Cumulative values of the discriminability indices (absolute d-prime, see STAR Methods*) across sessions. *Lines represent MOs (orange) and all other recorded brain regions (black). Arrows indicate the absolute d-prime values for the example neurons in (Figure 3B-D).* ***(H)*** *Significance for inter-region comparison of d-prime values from (G). d-prime values in MOs were significantly greater than all other regions for encoding auditory location and upcoming choice, but not significantly greater than ILA or ACA when considering visual location (Linear mixed effects model, see STAR Methods).* ***(I)*** *Proportion of neurons sensitive to visual (left) or auditory (right) stimulus location, estimated with combined conditions stimulus probability analysis (ccSP; see STAR Methods), after controlling for the other stimulus and for choice, using neural activity time-averaged over a window 0 to 300 ms after stimulus onset. In both cases, MOs has the highest proportion of significant neurons (more than 700 neurons were recorded in each area). Dashed line indicates the percentage of neurons expected by chance.* ***(J)*** *As in (I), but using combined conditions choice probability (ccCP; see STAR Methods) to estimate the proportion of neurons sensitive to the upcoming choice, after controlling for both stimuli, using neural activity time-averaged over a window 0 to 130 ms before movement onset.*

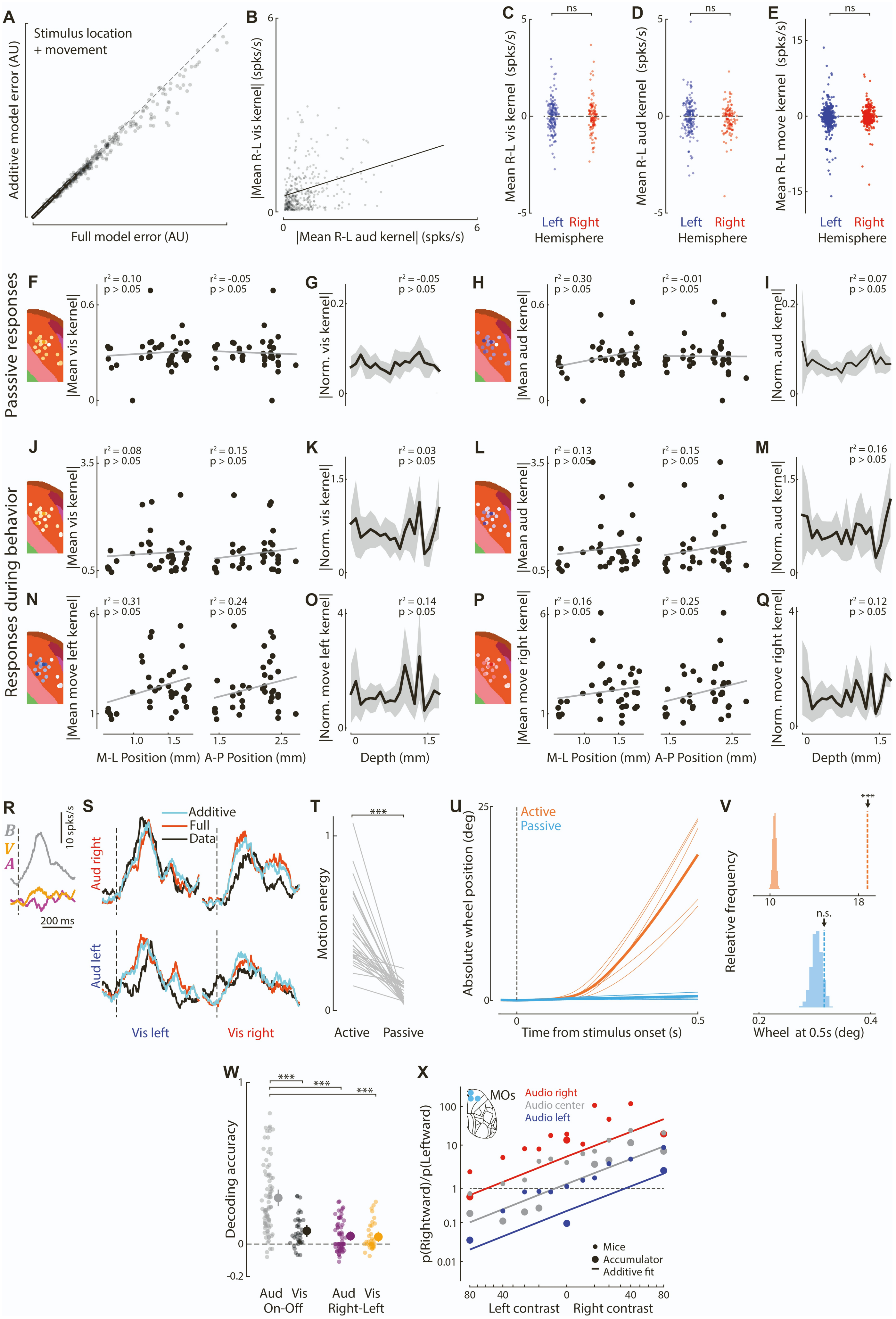
A
Stimulus location
+ movement
Additive model error (AU)
Full model error (AU)
B
|Mean R-L vis kernel| (spks/s)
|Mean R-L aud kernel| (spks/s)
C
Mean R-L vis kernel (spks/s)
D
Mean R-L aud kernel (spks/s)
E
Mean R-L move kernel (spks/s)
ns
Left Right
Hemisphere
Passive responses
F
G
H
I
|Mean vis kernel|
|Norm. vis kernel|
|Mean aud kernel|
|Norm. aud kernel|
r² = 0.10
p > 0.05
r² = -0.05
p > 0.05
r² = 0.30
r² = -0.01
r² = 0.07
Responses during behavior
J
K
L
M
r² = 0.08
r² = 0.15
r² = 0.03
r² = 0.13
r² = 0.16
N
O
P
Q
|Mean move left kernel|
|Norm. move left kernel|
|Mean move right kernel|
|Norm. move right kernel|
r² = 0.31
r² = 0.24
r² = 0.14
r² = 0.25
r² = 0.12
M-L Position (mm)
A-P Position (mm)
Depth (mm)
R
B
V
A
10 spks/s
200 ms
S
Additive
Full
Data
Aud right
Aud left
Vis left
Vis right
T
Motion energy
Active
Passive
U
Active
Passive
Absolute wheel position (deg)
Time from stimulus onset (s)
V
Relative frequency
n.s.
Wheel at 0.5s (deg)
W
Decoding accuracy
Aud Vis
On-Off
Aud Vis
Right-Left
X
MOs
Audio right
Audio center
Audio left
p(Rightward)/p(Leftward)
Mice
Accumulator
Additive fit
Left contrast
Right contrast

Figure S7 Additional analysis for neural recordings, related to Figure 4 and Figure 6

(A) *Prediction error for each neuron for the additive and full sensory-movement models (see STAR Methods). The full model has significantly higher error. (p < 0.01, 2183 cells, linear mixed-effects model). For visualization purposes, the largest 1% of errors were excluded from the plot, but not from statistical analysis.* ***(B)*** *Absolute value of time-averaged visual R-L kernel (V), versus absolute value of time-averaged auditory R-L kernel (A), after fitting the additive model under passive conditions for significant neurons. A time window from 0 to 300 ms after stimulus onset was used in both cases. Absolute responses are correlated, suggesting that if a neuron is predictable from spatial stimuli in one modality, it is more likely to be predictable from the other modality ($p < 10^{-5}$, 2509 cells, linear mixed-effects model).* ***(C)*** *Signed time average of the visual R-L kernel (V, Eqn 5 0 ms to 300 ms after stimulus onset) versus recorded hemisphere, after fitting the additive neural model ($N = 0$) under passive conditions ($D, M = 0$) (see STAR Methods). There is no significant lateralization in spatial preference (p > 0.05; 273 cells, linear mixed-effects model).* ***(D)*** *As in (C), but for the auditory R-L kernel (A). (p > 0.05; 287 cells).* ***(E)*** *As in (C), but the movement R-L kernel (D, -200 to 400 ms relative to movement onset), after fitting the additive model during active behavior (p > 0.05; 784 cells).* ***(F)*** *Left: each dot represents the mean position of MOs neurons located in a single probe. Color saturation indicates the magnitude of the mean absolute visual kernel size in neurons recorded from each probe during passive conditions. Right: the relationship between the mean absolute visual kernel of neurons in each probe and their medial-lateral position and anterior-posterior position (n = 35 penetrations, Pearson's correlation test).* ***(G)*** *Relationship between the mean absolute visual kernel size and depth from the brain surface in a model fitted to normalized firing rates (see STAR Methods). Black lines and grey shading indicated the median and ± 1 median absolute deviation across probes (n = 35 penetrations, Pearson's correlation test).* ***(H-I)*** *Same as (F-G) but for auditory kernel size.* ***(J-K)*** *Same as (F-G) but for neural activity recorded during the behavioral task (n = 36 penetrations).* ***(L-M)*** *Same as (J-K) but for auditory kernel size.* ***(N-O)*** *Same as (J-K) but for movement-left kernel size.* ***(P-Q)*** *Same as (J-K) but for movement-right kernel size.* ***(R)*** *Example sensory kernels from fitting the additive neural model to a single neuron under passive conditions. The selected neuron has opposing sensitivities for auditory (left-preference) and visual (right-preference) stimulus locations. B: baseline kernel; V: visual direction kernel; A: auditory direction kernel.* ***(S)*** *Cross-validated fit using the model kernels from (R) to neural responses under passive conditions for all audiovisual combinations. Cyan and orange lines show predictions of the additive and full models, black line shows test-set average responses.* ***(T)*** *Difference in facial video motion energy (see STAR Methods) when mice are performing the behavior (active) versus passive presentation of stimuli (passive). Mice exhibit significantly less motion under passive conditions. $***$: p < 0.001 (30 sessions, paired t-test).* ***(U)*** *Absolute wheel position, relative to position at stimulus onset, during active behavior (orange) and passive stimulus presentation (blue). Thin lines represent the average of all trials for individual mice (n = 5), thick lines represent the mean across mice. Dashed line indicates stimulus onset time.* ***(V)*** *To statistically test for increased wheel movement following stimulus onset, we used a randomization method. Dashed vertical lines indicate mean absolute wheel position 0.5 s after stimulus onset during active behavior (top) and passive stimulus presentation (bottom), averaged across mice. Histograms show the same statistic computed from 1000 "pseudosessions" where stimulus onset times were randomized (see STAR Methods). Wheel displacement was significantly larger than the shuffled distribution for active behavior ($***$: p < 0.001, shuffle test) but not during passive presentation (p > 0.05, shuffle test).* ***(W)*** *Neurons were tested individually to see if they encoded the presence or location of visual and auditory stimuli (see STAR Methods). The plot shows the single-neuron decoding accuracy of all significant neurons for each discrimination. Neurons encoding auditory stimulus presence (grey, 82 cells) have higher decoding accuracy than all other categories (36/59/36 cells for black/ magenta/gold). $***$: p < 0.001 (t-test).* ***(X)*** *Mean behavior of the accumulator model with the activity of all neurons in the right hemisphere reduced by 60% to simulate inactivation of right MOs (large circles), plotted with the mean behavior from MOs-inactivated mice (5 mice, small circles, cf. Figure 2H). Solid lines represent the fit of the additive model to the accumulator model output. This model cannot capture the rightward choice bias following right MOs inactivation because MOs neurons preferring either direction of stimulus are found equally in both hemispheres.*

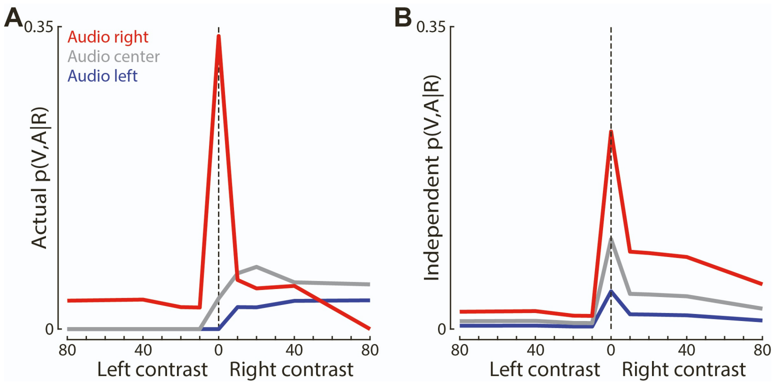

Figure S8 Conditional independence holds only approximately in our task, related to STAR Methods

(A) *The actual value of* $p(V, A \mid R)$ *for each contrast when the auditory stimulus was presented on the right/center/left (red/grey/blue). Values were computed by Bayes' theorem* $p(V, A \mid R) = p(R|V, A)p(V, A)/p(R)$*, summing* $p(V, A)$ *over ~156K trials from 17 mice.* ***(B)*** *As in (A), but for the conditional independence model* $p(V \mid R)p(A, \mid R)$*.*
