# Supplementary material for: Mouse frontal cortex mediates additive multisensory decisions
Source: Neuron. 2023 Aug 2;111(15):2432–2447.e13. doi: 10.1016/j.neuron.2023.05.008 (PMC10957398; doi:10.1016/j.neuron.2023.05.008)
Supplement: Document S2. Article plus supplemental information [file mmc2.pdf]

Neuron

Article

Mouse frontal cortex mediates additive multisensory decisions

Highlights

- Mice combine visual and auditory evidence additively to solve an audiovisual task
- Optogenetic inactivation indicates a key role for frontal cortex in the task
- After learning, neurons in frontal area MOs represent stimuli and predict choice
- An accumulator model applied to their activity reproduces mouse behavior

Authors

Philip Coen, Timothy P.H. Sit, Miles J. Wells, Matteo Carandini, Kenneth D. Harris

Correspondence

p.coen@ucl.ac.uk

In brief

Coen, Sit, et al. train mice in an audiovisual task and show that they combine evidence across modalities additively, even when it is conflicting. This combination relies on activity in frontal area MOs. After learning, this activity represents auditory and visual stimuli additively and can be used to reproduce behavior.

Check for updates

Coen et al., 2023, Neuron *111*, 2432–2447
August 2, 2023 © 2023 The Authors. Published by Elsevier Inc.
https://doi.org/10.1016/j.neuron.2023.05.008

CellPress

Article

Mouse frontal cortex mediates additive multisensory decisions

Philip Coen,[1,3,4,6,*] Timothy P.H. Sit,[2,4] Miles J. Wells,[1] Matteo Carandini,[3,5] and Kenneth D. Harris[1,5]
[1]UCL Queen Square Institute of Neurology, University College London, London, UK
[2]Sainsbury-Wellcome Center, University College London, London, UK
[3]UCL Institute of Ophthalmology, University College London, London, UK
[4]These authors contributed equally
[5]These authors contributed equally
[6]Lead contact
*Correspondence: p.coen@ucl.ac.uk
https://doi.org/10.1016/j.neuron.2023.05.008

SUMMARY

The brain can combine auditory and visual information to localize objects. However, the cortical substrates underlying audiovisual integration remain uncertain. Here, we show that mouse frontal cortex combines auditory and visual evidence; that this combination is additive, mirroring behavior; and that it evolves with learning. We trained mice in an audiovisual localization task. Inactivating frontal cortex impaired responses to either sensory modality, while inactivating visual or parietal cortex affected only visual stimuli. Recordings from >14,000 neurons indicated that after task learning, activity in the anterior part of frontal area MOs (secondary motor cortex) additively encodes visual and auditory signals, consistent with the mice's behavioral strategy. An accumulator model applied to these sensory representations reproduced the observed choices and reaction times. These results suggest that frontal cortex adapts through learning to combine evidence across sensory cortices, providing a signal that is transformed into a binary decision by a downstream accumulator.

INTRODUCTION

A simple strategy to combine visual and auditory signals, which is optimal if they are independent,[1,2] is to add them. Given independent visual evidence V and auditory evidence A, the log odds of a stimulus being on the right or left (R or L) is a sum of functions that each depend on only one modality (see derivation in STAR Methods):

$$\log\left(\frac{p(R \mid A,V)}{p(L \mid A,V)}\right) = \log\left(\frac{p(V \mid R)}{p(V \mid L)}\right) + \log\left(\frac{p(A \mid R)}{p(A \mid L)}\right) + \log\left(\frac{p(R)}{p(L)}\right) = f(V) + g(A) + b \quad \text{(Equation 1)}$$

Multisensory integration in humans and animals is often additive.[3–13] Nevertheless, some studies suggest that humans,[14–16] other primates,[17] and mice[18,19] can break this additive law. One way that the additive law could be broken is if one modality is dominant, meaning that if the modalities conflict, the non-dominant modality is ignored.[18]

In rodents and other mammals, neurons integrating across modalities have been observed in superior colliculus,[20–24] thalamus,[25–28] parietal cortex,[4,6,7,18,29–38] frontal cortex,[39] and possibly[40] even primary sensory cortices.[41–50] However, it is not clear which cortical areas support multisensory decisions or how multisensory signals are encoded by neuronal populations in these regions.[51] Perturbation studies have focused primarily on parietal cortex and disagree as to whether this region is[18] or is not[29,52,51] critical for multisensory behavior.

The brain could use different strategies to make multisensory decisions. For example, while visual and auditory cortices might be necessary and sufficient for behavioral responses to unisensory visual and auditory stimuli, a third region might be required for multisensory responses while playing no role in responses to either modality alone. Alternatively, unisensory and multisensory evidence could be processed by the same circuits: information from both senses may converge on a brain region that has a causal role in behavioral responses to both modalities, alone or in combination. If this region added evidence from the two modalities, it could drive behavior according to the additive law (Equation 1).

We studied an audiovisual localization task in mice and found support for the second hypothesis: multisensory evidence is processed by circuits that also process unisensory evidence, and these circuits involve the frontal cortex. Mouse behavior was consistent with the additive model (Equation 1). Optogenetic inactivation of visual or parietal cortex affected responses to visual stimuli only. Inactivating anterior frontal cortex (secondary motor area MOs) affected responses to

 © 2023 The Authors. Published by Elsevier Inc.
This is an open access article under the CC BY license (http://creativecommons.org/licenses/by/4.0/).

CellPress
OPEN ACCESS

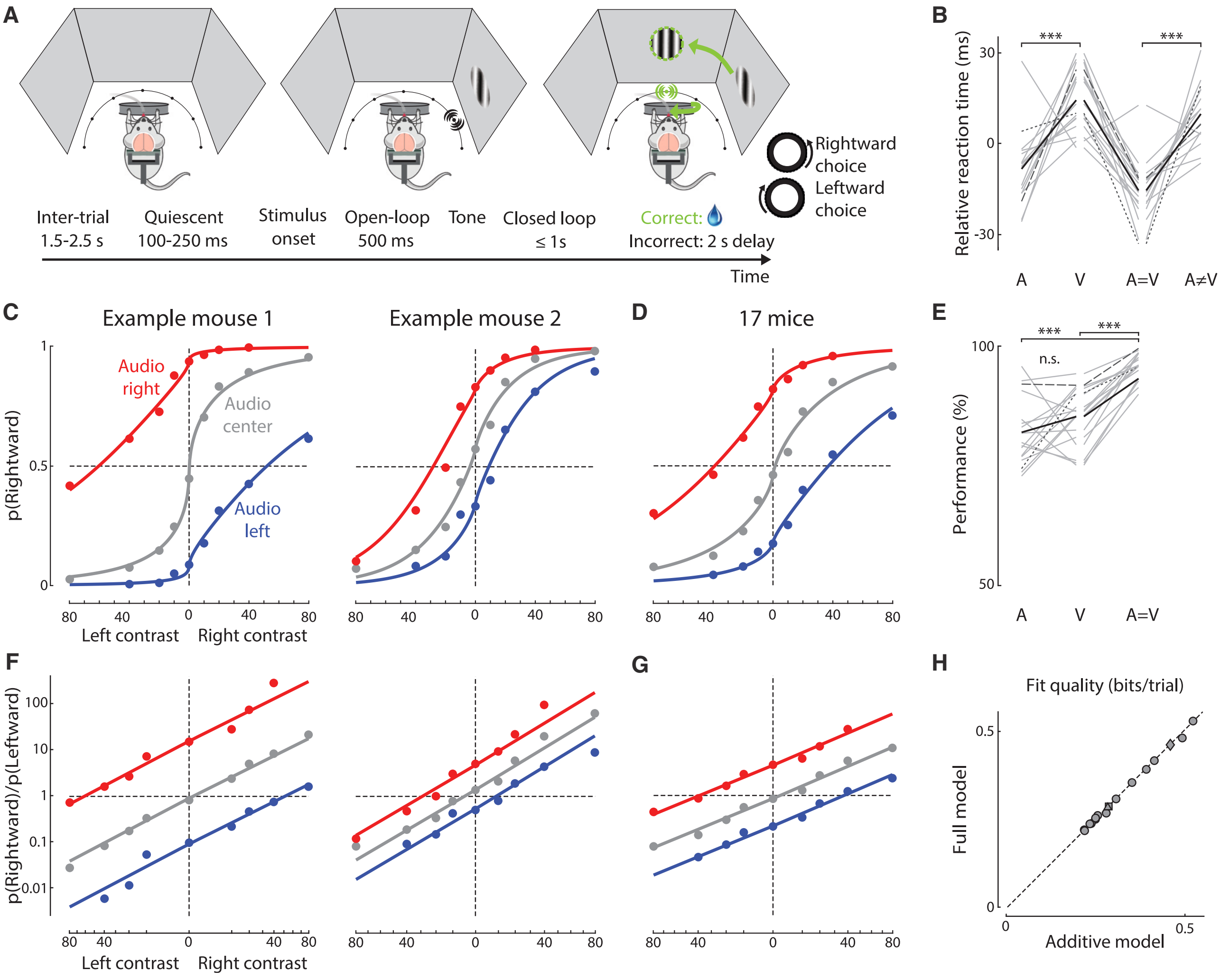

Figure 1. Spatial localization task reveals additive audiovisual integration

(A) Behavioral task. Top: visual and auditory stimuli are presented using 3 screens and 7 speakers. In the example, auditory and visual stimuli are presented on the right, and the subject is rewarded for turning the wheel counter-clockwise to center the stimuli (a "rightward choice"). Bottom: task timeline. After inter-trial interval of 1.5–2.5 s, mice must hold the wheel still for 100–250 ms. They then have 1.5 s to indicate their choice. During the first 500 ms of this period, the stimulus does not move ("open loop"), but during the final 1 s, stimulus position is yoked to wheel movement. After training, over 90% of choices occurred during open loop (Figures S1D–S1F).

(B) Median reaction times for each stimulus type, relative to mean across stimulus types. Only trials with 40% contrast were included. Gray lines: individual mice; black line: mean across 17 mice. Long and short dashes indicate example mice from left and right of (C).

(C) Fraction of rightward choices at each visual contrast and auditory stimulus location for two example mice. Curves: fit of the additive model.

(D) As in (C), but averaged across 17 mice (~156,000 trials). Curves: combined fit across all mice.

(E) Mouse performance (% rewarded trials) for different stimulus types ("correct" is undefined on conflict trials). Plotted as in (B).

(F and G) Data from (C) and (D), replotted as odds of choosing right vs. left (in log coordinates, Y axis) as a function of visual contrast raised to the power γ. Model predictions are straight lines.

(H) Log_2-likelihood ratio for the additive vs. full model where each combination of visual and auditory stimuli is allowed its own behavioral response that need not follow an additive law. (5-fold cross-validation, relative to a bias-only model). Triangles and diamonds: mice from left and right of (C). Squares: combined fit across 17 mice. There is no significant difference between models ($p > 0.05$). ***$p < 0.001$ (paired t test).

both modalities. Population recordings revealed that this region encoded stimuli of both modalities additively. Its sensory responses developed with task learning and persisted during passive stimulus presentation. An accumulator model applied to these passive responses reproduced the pattern of choices and reaction times observed in the mice.

RESULTS

We developed a two-alternative forced-choice audiovisual spatial localization task for mice (Figure 1A). We extended a visual task where mice turn a steering wheel to indicate whether a grating of variable contrast was on their left or right[53] by adding an array of

speakers. On each trial, at the time the grating appeared, the left, center, or right speaker played an amplitude-modulated noise. On coherent multisensory trials (auditory and visual stimuli on the same side), and on unisensory trials (zero contrast or central auditory stimulus), mice earned a water reward for indicating the correct side. On conflict multisensory trials (auditory and visual stimuli on opposite sides), or on neutral trials (central auditory and zero contrast visual), mice were rewarded randomly (Figure S1A). Mice learned to perform this task proficiently (Figure S1B), reaching 96% ± 3% correct (mean ± SD, n = 17 mice) for the easiest stimuli (coherent trials with the highest contrast).

Mice responded fastest in coherent trials with high-contrast visual stimuli (Figure 1B). Reaction times were typically 190 ± 120 ms (median ± MAD, n = 156,000 trials in 17 mice) and were 22 ± 20 ms faster in unisensory auditory than unisensory visual trials (mean ± SD, n = 17 mice, $p < 0.001$, paired t test), suggesting that the circuits responsible for audiovisual decisions receive auditory signals earlier than visual signals[54,55] (Figures 1B, S1M, S1O, and S1Q). In multisensory trials, reaction times were 25 ± 18 ms faster for coherent than conflict trials ($p < 0.001$, paired t test). This suggests that multisensory inputs feed into a single integrator, rather than two unisensory integrators racing independently to reach threshold.[5,56] Reaction times were faster at higher contrasts for unisensory visual and coherent multisensory trials ($p < 0.001$, linear mixed-effects model) and were possibly even faster in coherent trials than unisensory auditory trials, particularly at high contrast levels ($p < 0.08$, paired t test).

Spatial localization task reveals additive audiovisual integration

Mice used both modalities to perform the task, even when the two were in conflict. The fraction of rightward choices, which depended smoothly on visual contrast, further increased or decreased when sounds were on the right or on the left (Figures 1C and 1D, red vs. blue). Mice performed more accurately on coherent trials than unisensory trials (Figures 1E, S1N, and S1P), indicating that they attended to both modalities.[8,12,54,55,57,58]

To test whether mice make multisensory decisions additively, we fit the additive model to their choices. Equation 1 can be rewritten as:

$$p(R) = \sigma(f(V) + g(A) + b) \quad \text{(Equation 2)}$$

where $p(R)$ is the probability of making a rightward choice, and $\sigma(x) = 1/(1 + \exp(-x))$ is the logistic function. We first fit this model with no constraints on the functions f and g and found that it provided excellent fits (Figure S2F). We further simplified it by modeling f with a power function to account for contrast saturation in the visual system[59]:

$$f(V) = v_R V_R^{\gamma} - v_L V_L^{\gamma} \quad \text{(Equation 3)}$$

$$g(A) = a_R A_R - a_L A_L \quad \text{(Equation 4)}$$

Here V_R and V_L are right and left contrasts (at least one of which was always zero), and A_R and A_L are indicator variables for right and left auditory stimuli (with value 1 or 0 depending on the auditory stimulus position). This model performed almost as well as the 11-parameter unconstrained model (Figure S2F) with only 6 free parameters: bias (b), visual exponent (γ), visual sensitivities (v_R and v_L), and auditory sensitivities (a_R and a_L). In the rest of the paper, we thus adopted this simplified version of the additive model.

The additive model provided excellent fits to the multisensory decisions of all mice. It fit both the choices of individual mice (Figure 1C) and the choices averaged across mice (Figure 1D). A simple view of these data can be obtained by representing them in terms of log odds of rightward choices (as in Equation 1) vs. linearized contrast (contrast raised by the exponent γ, Equation 3). As predicted by the model, the responses to unisensory visual stimuli fall on a line, and auditory cues shift this line additively (Figures 1F, 1G, and S3A–S3O). The intercept of the line is determined by the bias b, the slope by the visual sensitivity v, and the additive offset by the auditory sensitivity a.

The additive model performed better than non-additive models, including models where one modality dominates the other (Figures S2A–S2E). It performed as well as a full model, which used 25 parameters to fit the response to each stimulus combination, without additive constraints (Figure 1H). The additive model could be fit from the unisensory choices alone, indicating that mice use the same behavioral strategy on coherent and conflict trials (Figure S3P). As predicted by the additive model, equal and opposite auditory and visual stimuli (i.e., stimuli eliciting an equal probability of left and right choices when presented alone) led to neutral behavior when presented together, i.e., a 50% chance of left or right choices (Figure S2G). In contrast, a model of sensory dominance would predict that these stimuli lead to choices determined by the dominant modality.

Optogenetic inactivation identifies roles of sensory and frontal cortical areas

To determine which cortical regions are necessary to perform the task, we used laser-scanning optogenetic inactivation across 52 sites in dorsal cortex. We inactivated with transcranial laser illumination in mice expressing ChR2 in parvalbumin interneurons[59–62] (3 mW; 462 nm; 1.5 s duration following stimulus onset; Figure 2A). We combined results across mice and hemispheres because they were qualitatively consistent and symmetric (Figures S4A and S4B). Control measurements established that mouse choices were unaffected by target locations just outside the brain (Figure S4C). Because of light scattering in the brain, we expect inactivation to impact areas ~1 mm from the target location[59,63,64] (Figure 2A). For this reason, and because brain curvature hides auditory cortex, laser sites between primary visual and auditory areas likely inactivated both visual and auditory cortices. We refer to these sites as "lateral sensory cortex." We found that inactivating them impacted the choices, as did inactivating visual and frontal areas. However, inactivating these different regions had distinct impacts on task performance, which we detail below.

Inactivating visual cortex impaired visual but not auditory choices. As seen in visual tasks,[53,59,65] inactivation of visual cortex reduced responses to contralateral visual stimuli, whether presented alone (Figure 2B) or with auditory stimuli (Figures 2D and 2E). It had a smaller effect in coherent trials, when those choices could be based on audition alone (Figure 2D, $p < 0.01$,

CellPress
OPEN ACCESS

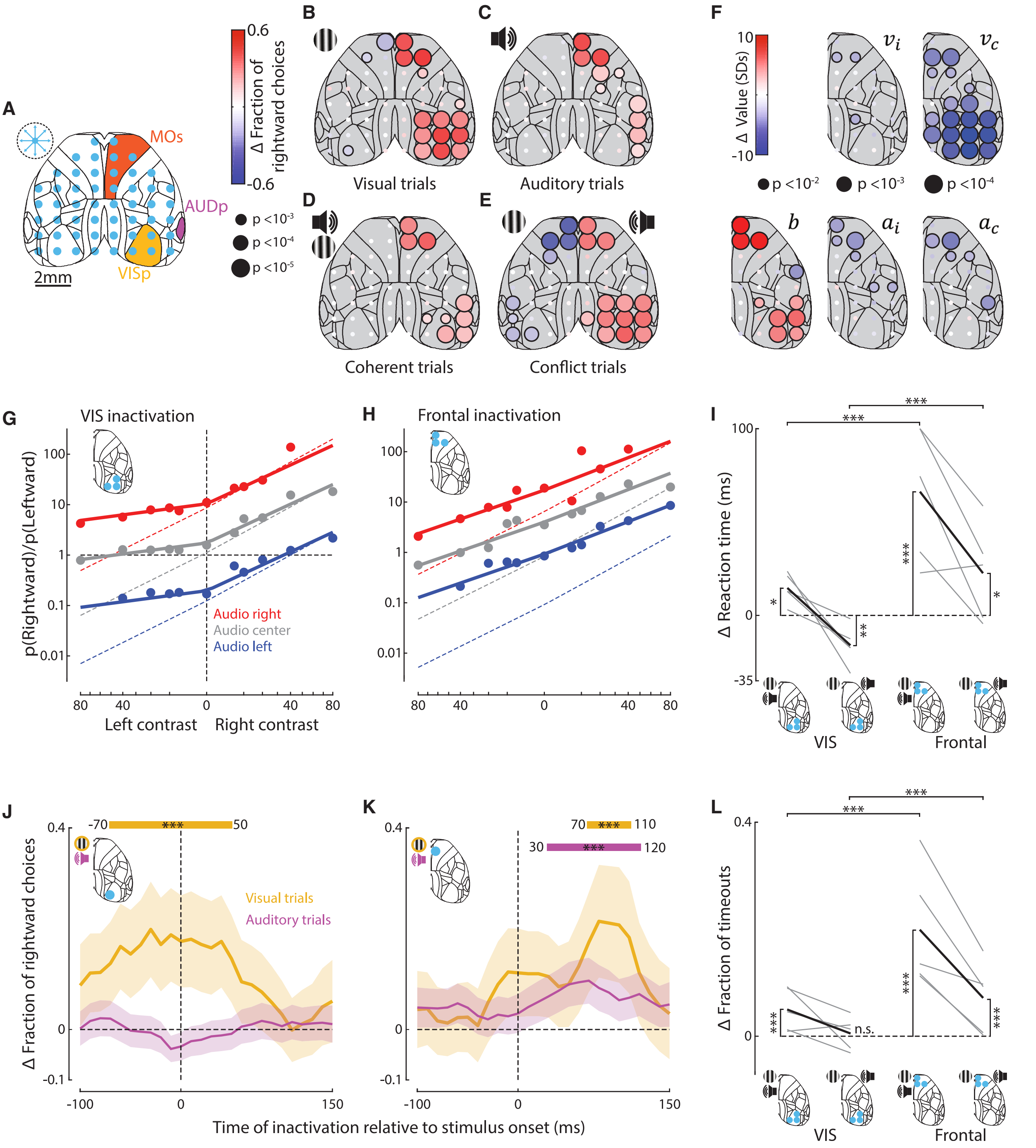

Figure 2. Optogenetic inactivation identifies roles of sensory and frontal cortical areas

(A) Schematic of inactivation sites. On ~75% of trials, a blue laser randomly illuminated one of 52 sites (blue dots) for 1.5 s following stimulus onset. Dashed circle: estimated radius (1 mm) of effective laser stimulation. Yellow, orange, and magenta: primary visual region (VISp), primary auditory region (AUDp), and secondary motor cortex (MOs).

(B) Change in the fraction of rightward choices for each laser site for unisensory left visual stimulus trials. Red and blue dots: increases and decreases in fraction of rightward choices; dot size represents statistical significance (5 mice, shuffle test, see STAR Methods). Data for right stimulus trials were included after reflecting the maps (see Figure S4A for both individually).

(C) As in (B), but for unisensory auditory trials.

(legend continued on next page)

paired t test across 5 mice). Conversely, it did not affect unisensory auditory choices (Figure 2C), indicating that visual cortex does not play a substantial role in processing auditory signals in this task. Finally, bilateral inactivation of visual or parietal cortex reduced the fraction of choices toward the location of the visual stimulus in both unisensory visual and multisensory trials (Figures S5U–S5X).

Inactivating frontal cortex impaired choices based on either modality with similar strength, suggesting a role in integrating visual and auditory evidence (Figures 2B–2E). On visual trials, inactivating frontal cortex had a similar effect as inactivating visual cortex: it reduced responses to contralateral stimuli (Figure 2B, as in visual detection tasks[53,59,65]). However, it also caused a similar reduction in the responses to contralateral auditory stimuli ($p > 0.05$, t test across mice; Figure 2C). In coherent multisensory trials, frontal inactivation reduced responses to contralateral stimuli (Figure 2D). On conflict trials, it reduced the responses to the contralateral stimulus, whichever modality it came from (Figure 2E). Bilateral inactivation of frontal cortex slowed responses but did not bias the animal's choices in either direction for any stimulus type (Figures S5Q–S5X).

Finally, inactivating lateral sensory cortex strongly impaired visual choices and weakly impaired auditory choices. It decreased correct responses to contralateral stimuli whether visual alone (Figure 2B), auditory alone (Figure 2C), or combined (Figures 2D and 2E) but had a larger effect on visual than auditory choices (Figures 2B and 2C, $p < 0.05$, t test across mice). These results might suggest a multisensory role but might, more simply, arise from light spreading into both visual and auditory areas: indeed, because of brain curvature, light likely passes through overlying tissue before reaching auditory cortex, which is required for auditory localization.[66] Attenuation by this overlying tissue may explain the weaker effect on auditory choices. The minor effect of inactivating somatosensory cortex (Figure S4F) may also arise from light spreading.

The results of these inactivations were well captured by the additive model. The model accounted for the effects of inactivating visual cortex via a decrease in the sensitivity for contralateral visual stimuli v_c (Figure 2F), which reduced performance for contralateral visual stimuli regardless of auditory stimuli (Figure 2G). Inactivating lateral sensory cortex had a similar effect and more weakly decreased contralateral auditory sensitivity a_c (Figures 2F and S4E, $p < 0.07$, t test across mice). Inactivating frontal cortex reduced visual and auditory sensitivity by a similar amount ($p > 0.65$, t test across mice) and increased bias b to favor ipsilateral choices (Figures 2F, 2H, and S4G). The model revealed that the effects of inactivating visual, lateral, and frontal cortices were statistically different from each other (Figure S4H). For example, inactivating frontal cortex reduced sensitivity to both contralateral and ipsilateral stimuli, but inactivating lateral sensory cortex only reduced sensitivity to contralateral stimuli (Figure 2F).

The effect of inactivation on reaction times revealed a difference between frontal and other cortices. Inactivating frontal cortex delayed responses in all stimulus conditions (Figures 2I and S5A–S5P). In contrast, the effect of inactivating visual cortex depended on the stimulus condition: responses to contralateral visual stimuli or coherent contralateral audiovisual stimuli were delayed, but responses to conflicting stimuli with a contralateral visual component were accelerated (Figures 2I and S5A–S5H). It effectively caused the mouse to ignore the contralateral visual stimulus and respond as on unisensory auditory trials (Figure 1B). The effects of inactivating the lateral cortex were similar to visual cortex but did not reach statistical significance. Similar results were seen in the fraction of timeouts, i.e., trials where the mouse failed to respond within 1.5 s (Figures 2L and S5I–S5P). Bilateral inactivation of visual or parietal cortex delayed responses to unisensory visual or coherent multisensory trials, while bilateral inactivation of frontal cortex delayed responses to all trial types (Figures S5Q–S5T). These data indicate that inactivating visual or lateral cortex mimicked the absence of a contralateral stimulus, which may speed or slow reaction times depending on whether this absence resolves a conflict. They also indicate that inactivating frontal cortex slows all choices, consistent with a process of multisensory evidence integration and possibly also of premotor planning or motor execution.

The critical time window for inactivation was earlier for visual cortex than for frontal cortex (Figures 2J and 2K). We used 25-ms laser pulses to briefly inactivate visual and frontal cortex at different times relative to stimulus onset on unisensory trials[59] (see STAR Methods). Inactivating right visual cortex significantly increased the fraction of rightward choices if the laser pulse

(D) As in (B), but for coherent multisensory trials.

(E) As in (B), but for conflict multisensory trials.

(F) As in (B)–(E), but dot color indicates the change in parameters of the additive model. b, bias toward ipsilateral choices (relative to inactivation site); v_i and v_c, sensitivity to ipsilateral and contralateral contrast; a_i and a_c, sensitivity to ipsilateral and contralateral auditory stimuli.

(G) Fit of additive model to trials when a site in visual cortex was inactivated. Dashed lines: model fit to non-inactivation trials. Trials with inactivation of left visual cortex were included after reflecting the maps (5 mice, 6,497 trials). Inactivation significantly changed model parameters (paired t test, $p < 0.05$).

(H) As in (G), but for trials when frontal cortex was inactivated (5 mice, 5,612 trials). Inactivation significantly changed model parameters (paired t test, $p < 0.05$).

(I) Change in multisensory reaction times when visual or frontal cortex was inactivated contralateral to the visual stimulus. Gray and black lines: individual mice (n = 5) and the mean across mice. Reaction times are the mean across the medians for each contrast relative to non-inactivation trials. Values above 100 ms were truncated for visualization. On coherent trials, inactivating visual or frontal cortex increased reaction time, with larger effect for frontal. On conflict trials, inactivation of visual cortex decreased reaction time while inactivation of frontal cortex caused an increase. *$p < 0.05$, **$p < 0.01$, *$p < 0.001$ (linear mixed-effects model).

(J) Change in fraction of rightward choices when contralateral visual cortex was inactivated on visual (yellow, 519 trials) or auditory (magenta, 1,205 trials) trials. Inactivation was a 25 ms, 25 mW laser pulse at different time points. Curves show average over mice smoothed with a 70 ms boxcar window. Shaded areas: 95% binomial confidence intervals. *** indicates intervals where fraction of rightward choices differs significantly from controls ($p < 0.001$, Fisher's exact test).

(K) As in (J), but for frontal inactivation (451 and 1,291 trials for auditory and visual conditions).

(L) As in (I), but for the change in the fraction of timeout trials. On coherent trials, inactivation of either visual or frontal cortex significantly increased timeouts. On conflict trials, only frontal inactivation changed the fraction of timeouts. ***$p < 0.001$ (linear mixed-effects model).

CellPress
OPEN ACCESS

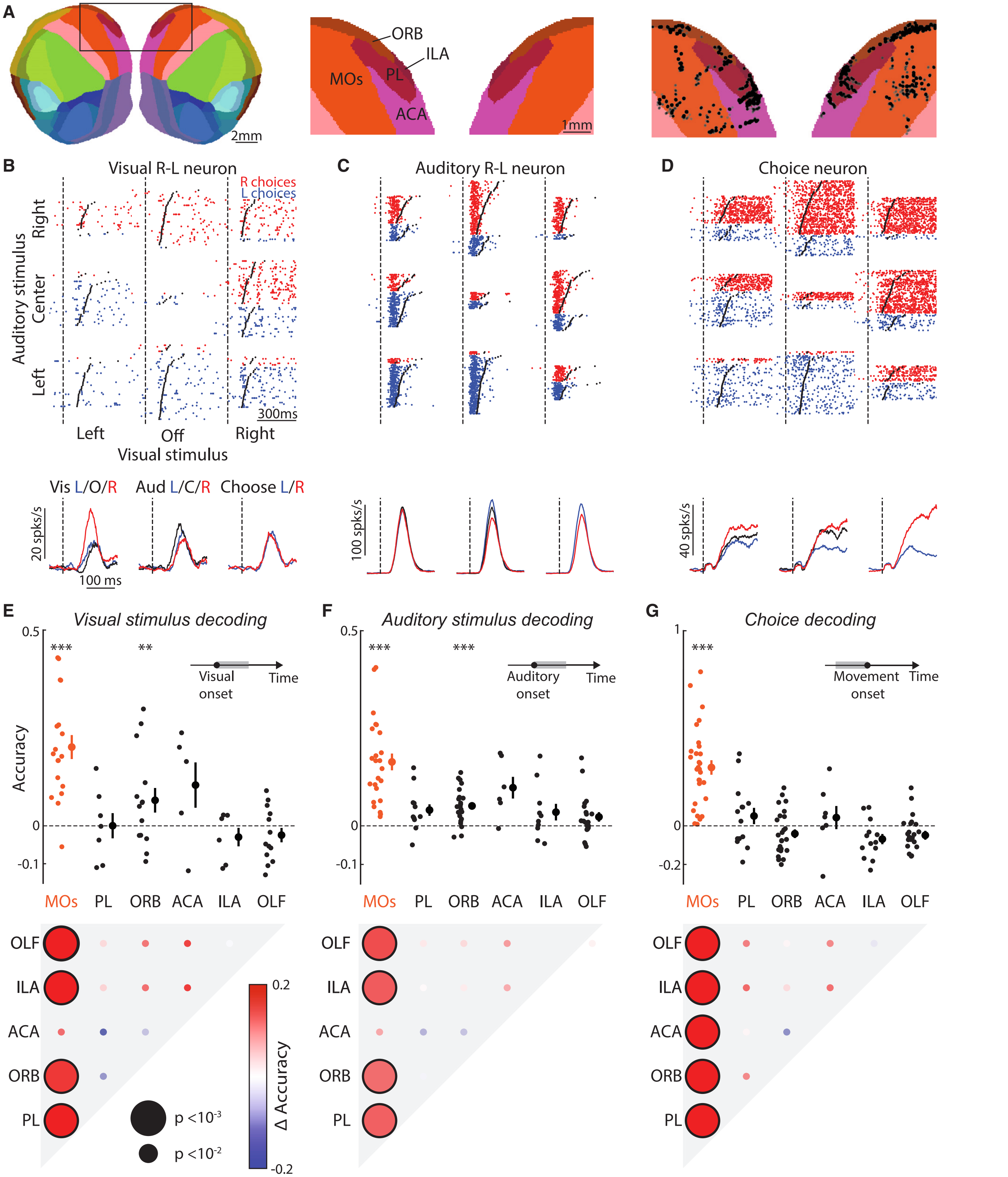

(legend on next page)

began between 70 ms prior and 50 ms after the appearance of a visual stimulus on the left ($p < 0.001$), but had no significant effect at any time after an auditory stimulus (Figure 2J); an impact of inactivation prior to stimulus onset likely results from continued suppression of neural activity following laser offset.[59] Frontal inactivation impacted behavior later: 70–110 ms after contralateral visual stimuli and 30–120 ms after contralateral auditory stimuli (Figure 2K). The earlier critical window for frontal inactivation on auditory trials is consistent with the faster reaction times on these trials (Figure 1B). However, in both cases, inactivation of frontal cortex had no significant effect >120 ms after stimulus onset, suggesting that after this time, frontal cortex plays a limited role in sensory integration. These short inactivation pulses had no significant effect when stimuli were ipsilateral to the inactivation or when the laser was targeted outside the brain.

Together, these results suggest that visual cortex's role in the task is to relay visual information to downstream structures including frontal cortex, which integrates it with auditory information from elsewhere to shape the mouse's choice, with this whole process occurring over ~120 ms.

Neurons in frontal area MOs encode stimuli and predict behavior

The results of frontal inactivation suggest that at least some neurons in frontal cortex may integrate evidence from both modalities. To test this hypothesis, we recorded acutely with Neuropixels probes during behavior (Figures 3A–3D). We recorded 14,656 neurons from frontal cortex across 88 probe insertions (56 sessions) from 6 mice (Figures 3A, S6A, and S6B) divided across the following areas: MOs, orbitofrontal (ORB), anterior cingulate (ACA), prelimbic (PL), infralimbic (ILA), and nearby olfactory areas (OLF). These regions exhibited a variety of neural responses, including neurons that were sensitive to visual and auditory location (Figures 3B and 3C) and to the animal's upcoming choice (Figure 3D).

Among these frontal regions, task information was represented most strongly in MOs. MOs was the only region able to predict the animal's upcoming choice before movement onset (Figures 3G and S6C) and encoded auditory and visual stimulus location significantly more strongly than the other regions (Figures 3E and 3F; $p < 0.01$, linear mixed-effects model; the difference with ACA did not reach significance). Activity in MOs began to predict the animal's choice ~100 ms before movement onset (Figure S6E) and was more accurate for neurons more anterior or lateral within MOs (Figure S6F). Furthermore, choice decoding from MOs activity was more accurate on sessions with higher behavioral performance ($p < 0.05$, linear mixed-effects model; Figure S6F), suggesting a link between MOs choice coding and behavioral engagement. Analysis of single cells yielded results consistent with population decoding: MOs neurons better discriminated stimulus location and choice than neurons of all other regions (Figures S6G–S6H; $p < 0.05$, linear mixed-effects model; differences with ACA and PL did not reach significance for visual location). These observations were robust to the correlation between stimuli and choices: even when controlling for this correlation, MOs still had the largest fraction of neurons with significant coding of stimulus location or pre-movement choice (Figures S6I–S6J). Once movements were underway, however, we could decode their direction from multiple regions, consistent with observations that ongoing movements are encoded throughout the brain[68,69] (Figure S6D).

Frontal area MOs integrates task variables additively

Given the additive effects of visual and auditory signals on behavior, we asked whether these signals also combine additively in MOs. To analyze MOs responses to combined audiovisual stimuli during behavior, we used an ANOVA-style decomposition into temporal kernels.[70] We focused on audiovisual trials of a single contrast so we could define binary variables $a_i, v_i, c_i = \pm 1$, encoding the laterality (left vs. right) of auditory stimuli, visual stimuli, and choices. The population firing rate vector $\boldsymbol{F}_i(t)$, on trial i at time t after stimulus onset, decomposed as the sum of 6 temporal kernels:

$$\boldsymbol{F}_i(t) = \boldsymbol{B}(t) + a_i\boldsymbol{A}(t) + v_i\boldsymbol{V}(t) + a_iv_i\boldsymbol{N}(t) + \boldsymbol{M}(t-\tau_i) + c_i\boldsymbol{D}(t-\tau_i) \quad \text{(Equation 5)}$$

Here, $\boldsymbol{B}$ is the mean stimulus response averaged across stimuli, $\boldsymbol{A}$ and $\boldsymbol{V}$ are the additive effects of auditory and visual stimulus location, and $\boldsymbol{N}$ is a potential non-additive interaction between them. Finally, $\boldsymbol{M}$ is a kernel for the mean effect of movement (regardless of direction and relative to the time τ_i of movement onset on trial i) and $\boldsymbol{D}$ is the differential effect of movement direction (right minus left). To test for additivity, we compared the

Figure 3. Neurons in frontal area MOs encode stimuli and predict behavior

(A) Recording locations for cells (black dots, right) overlaid on a flattened cortical map (using the Allen Common Coordinate Framework[67]), showing locations in secondary motor cortex (MOs, 3,041 neurons), orbitofrontal (ORB, 5,112), anterior cingulate (ACA, 727), prelimbic (PL, 1,332) and infralimbic (ILA, 1,254) areas.
(B) Top: spike rasters, separated by trial condition, from a neuron sensitive to visual spatial location (d' = 1.85). Red/blue rasters: trials with a rightward/leftward mouse choice. Dashed line and black points: stimulus onset and movement initiation. Bottom: peri-stimulus time histogram (PSTH) of the neural response, averaged across different visual (left), auditory (center), or choice (right) conditions. Trials are not balanced; choice and stimulus location are correlated.
(C) As in (B), for a neuron sensitive to auditory spatial location (d' = −0.81).
(D) As in (B), for a neuron sensitive to the animal's choice (d' = 2.61).
(E) Top: cross-validated accuracy (relative to a bias model, see STAR Methods) of a support vector machine decoder trained to predict visual stimulus location from population spiking activity time-averaged from 0 ms to 300 ms after stimulus onset. Accuracies 0 and 1 represent chance and optimal performance. Points: decoding accuracy from neurons in regions labeled in (A), or olfactory areas (OLF, 2,068 neurons), for one experimental session. Neurons were subsampled to equalize numbers across points. ***$p < 0.001$, **$p < 0.01$ (≥5 sessions from 2 to 5 mice for each region, one-sided t test). Bottom: inter-regional comparison of decoding accuracy (linear mixed-effects model). Black outlines: statistically significant difference. Dot size: significance level.
(F) As in (E), for decoding of auditory stimulus location (≥6 sessions, 3–6 mice).
(G) As in (E), for decoding choices from spiking activity 0–130 ms preceding movement (≥7 sessions, 3–6 mice).

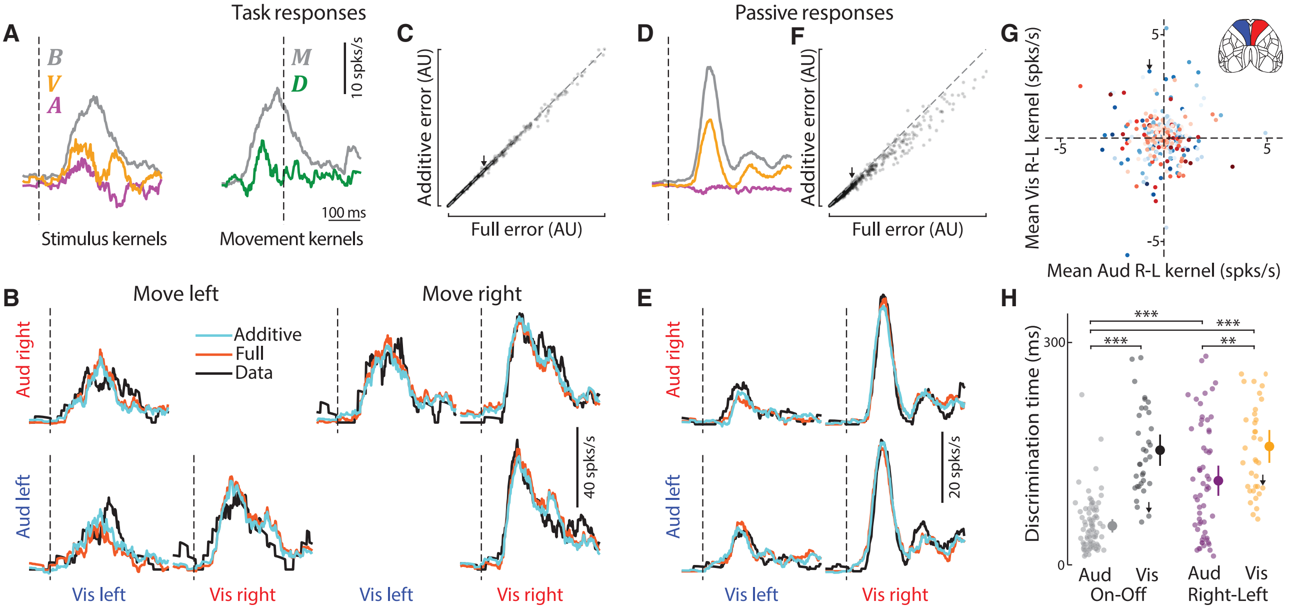

Figure 4. Frontal area MOs encodes task variables additively

(A) Kernels from fitting the additive neural model to an example neuron. Dashed lines: stimulus onset (left) or movement onset (right). *B*, mean stimulus response; *A* and *V*, additive effects of auditory and visual stimulus location; *M*, mean effect of movement (relative to τ_i, movement onset time on trial *i*); *D*, differential effect of movement direction (right minus left). The non-additive kernel *N* was set to 0.

(B) Cross-validated model fits to average neural activity in audiovisual conditions for the neuron from (A). Coherent trials with incorrect responses were too rare to include. Cyan and orange lines: predictions of additive ($N = 0$) and full models. Black line: test-set average responses. Dashed lines: stimulus onset.

(C) Prediction error (see STAR Methods) across all neurons for additive and full models. Arrow indicates example cell from (A and B). The additive model has a smaller error (p = 0.037, linear mixed-effects model, 2,183 cells, 5 mice). Top 1% of errors were excluded for visualization, but not analyses.

(D–F) As in (A)–(C), but for neural activity during passive stimulus presentation, using only sensory kernels. In (F), $p < 10^{-10}$ (2,509 cells, 5 mice, linear mixed-effects model).

(G) Encoding of visual vs. auditory stimulus preference (time-averaged kernel amplitude for *V* vs. *A*) for each cell. There was no significant correlation between *V* and *A*. p > 0.05 (2,509 cells, Pearson correlation test). Red/blue: cells recorded in right/left hemisphere. Color saturation: fraction of variance explained by sensory kernels.

(H) Discrimination time (see STAR Methods) relative to stimulus onset during passive conditions. Auditory Right-Left neurons (magenta, n = 59) discriminated stimulus location earlier than Visual Right-Left neurons (gold, n = 36). Auditory On-Off neurons (sensitive to presence, but not necessarily location, gray, n = 82) discriminated earliest, even compared to Visual On-Off neurons (n = 36, black). Points: individual neurons. Bars: standard error. **p < 0.01, ***p < 0.001 (Mann–Whitney U test).

cross-validated performance of this full model against an additive model where $\boldsymbol{N} = 0$.

The results were consistent with additive integration of visual and auditory signals. The additive model of MOs responses outperformed the full model with interactions between visual and auditory stimuli (Figures 4A–4C), as well as an alternative full model with interactions between stimuli and movement (Figure S7A). (Better performance of the additive model reflects over-fitting of the full model, whose parameters are a superset of the additive model's.) Similar results were seen during passive presentation of the task stimuli, when sensory responses could not be confounded by movement (Figures 4D–4F and S7T–S7V).

MOs neurons provided a mixed representation of visual and auditory stimulus locations but encoded the two modalities with different time courses. Similar to the mixed multisensory selectivity observed in the parietal cortex of rat[29] and primate,[6] the auditory and visual stimulus preferences of MOs neurons were neither correlated nor lateralized: cells in either hemisphere could represent the location of auditory or visual stimuli with a preference for either location and could represent the direction of the subsequent movement with a preference for either direction (Figures 4G, S7C–S7E, S7R, and S7S). We could not detect a significant correlation of kernel size with recording location in MOs, although there was a trend toward larger choice kernels in anterior and lateral regions (Figures S7F–S7Q). Neurons that responded to one modality, however, also tended to respond to the other, as evidenced by a weak correlation in the absolute sizes of the auditory and visual kernels (Figure S7B). Nevertheless, representations of auditory and visual stimuli had different time courses: neurons could distinguish the presence and location of auditory stimuli earlier than for visual stimuli (Figure 4H). This is consistent with the more rapid behavioral reactions to auditory stimuli (Figure 1B) and the earlier critical window for MOs inactivation on unisensory auditory than visual trials (Figures 2J and 2K). Indeed, the earliest times in which MOs encoded visual or auditory stimuli (Figure 4H) matched the times for which MOs inactivation impacted behavioral performance (Figures 2J and 2K). This delay between visual and auditory signals resembles the delay previously observed between visual and vestibular signals.[38]

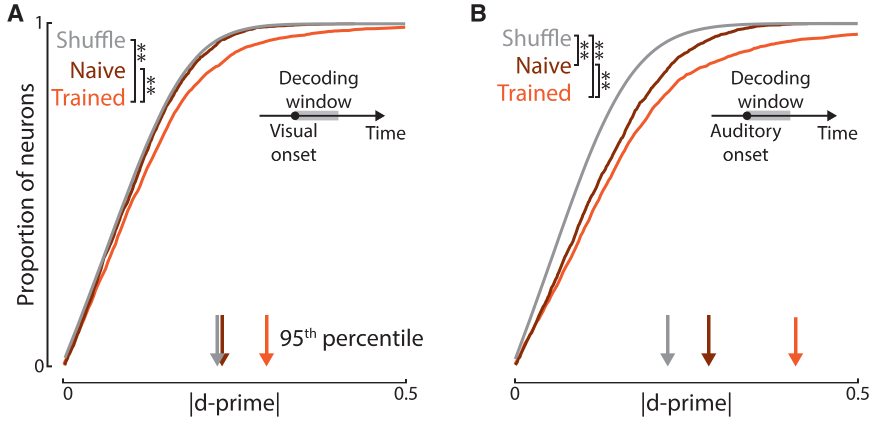

Figure 5. Audiovisual integration in MOs develops through learning

(A) Cumulative histogram of absolute visual discriminability index (d') scores for MOs neurons in naive mice (n = 2,700), trained mice (n = 2,956), and shuffled data. Training enriches the proportion of spatially sensitive neurons (**$p < 0.01$, Welch's t test). Naive mouse data was not significantly distinct from shuffled ($p > 0.05$, Welch's t test). Arrows: 95th percentile for each category.

(B) As in (A), but for auditory stimuli. Training enriches the proportion of spatially sensitive neurons, although naive mouse data was significantly distinct from shuffled data (**$p < 0.01$, Welch's t test, n = 2,698/2,946 neurons for naive/trained mice).

MOs encoded information about auditory onset (regardless of sound location) more strongly and earlier than information about visual onset or the location of either stimulus (Figures 4H and S7W). This may explain why mice exhibit auditory dominance in multisensory conflict trials in a detection task[18,19] but not in our localization task.

Multisensory signals develop in MOs after task training

Neural populations of MOs encoded auditory and visual location more strongly in task-proficient mice (Figures 5A and 5B). We recorded the responses of 2,702 MOs neurons to the task stimuli in 4 naive mice during passive conditions with no instructed movements and compared their activity to that previously characterized in trained mice. MOs encoding of visual stimulus location was significantly higher in trained mice than naive mice (**$p < 0.01$, Welch's t test). In naive mice, individual MOs neurons showed no coding of visual position: their d' index (absolute mean difference of firing rates between stimulus conditions divided by mean trial-to-trial SD) was not significantly different from a shuffled control (Figure 5A). In naive mice, MOs did encode auditory location ($p < 0.01$, Welch's t test), but this encoding grew stronger after task training (Figure 5B, $p < 0.01$, Welch's t test). We conclude that training enhances sensory responses in MOs, particularly visual responses.

An accumulator applied to MOs activity reproduced decisions

Given that the MOs population code resembled the animals' behavior in multiple ways, including the additive coding of visual and auditory stimuli and the earlier auditory responses, we next asked if the representation of multisensory task stimuli in MOs could explain the properties of the animals' choices. We considered an accumulator model that makes choices based on the stimulus representation in MOs (Figures 6A and 6B). To isolate the stimulus representation and avoid the confound of movement encoding in MOs, we used passive stimulus responses and generated surrogate population spike trains $\boldsymbol{x}(t)$ by selecting (from all recordings) MOs neurons encoding the location of at least one of the sensory stimuli. These spike trains were fed into an accumulator model[1,37,71–73]; they were scaled by a weight vector $\boldsymbol{w}$ and linearly integrated over time to produce a scalar decision variable $d(t)$:

$$d(t) = d(t-1) + \boldsymbol{w} \cdot \boldsymbol{x}(t) \quad \text{(Equation 6)}$$

The model chooses left or right when $d(t)$ crosses one of two decision boundaries placed at ± 1.

Although the model parameters were fit independently of mouse behavior, the model matched the average behavior of each mouse, as long as it was applied to MOs activity recorded in trained mice. Given an MOs representation $\boldsymbol{x}(t)$, we found the weight vector $\boldsymbol{w}$ that produced the fastest and most accurate choices possible (see STAR Methods). The model reproduced the different behavioral reaction times for different stimulus types: faster in auditory and coherent trials, and slower in visual and conflict trials (Figure 6D; cf. Figure 1B). Furthermore, as observed with mice, the model integrated multisensory stimuli additively (Figure 6E; cf. Figure 1G). In contrast, an accumulator model trained on MOs representations in naive mice failed to reproduce mouse behavior, with no significant difference in model performance between shuffle and test data ($p > 0.05$; Figures 6C and 6F). These results suggest that behavioral features of the responses, such as the different reaction times for auditory and visual stimuli and the additivity of visual and auditory evidence, reflect features in the MOs population code that appear only after the task has been learned.

The accumulator model even predicted the outcome of inactivation. Suppressing MOs neurons preferring left visual stimuli in the model reproduced the effects of inactivating right visual cortex (Figure 6G; cf. Figure 2G). However, the simple accumulator model could not reproduce the rightward bias observed with right MOs inactivation (Figure S7X; cf. Figure 2H) because MOs neurons preferring either stimulus position are found equally in both hemispheres (Figures S7C–S7E). To reproduce these effects, we made the additional assumption that the MOs neurons projecting to the downstream integrator from a given hemisphere were those preferring contralateral stimuli.[74] In practice, this means that weights from neurons in the left vs. right hemisphere must be positive vs. negative. This refined model predicted the lateralized effect of MOs inactivation (Figure 6H). These results support the hypothesis that MOs neurons learn to additively integrate evidence from visual and auditory cortices, producing a population representation that is causally and selectively sampled by a downstream circuit that makes decisions.

CellPress
OPEN ACCESS

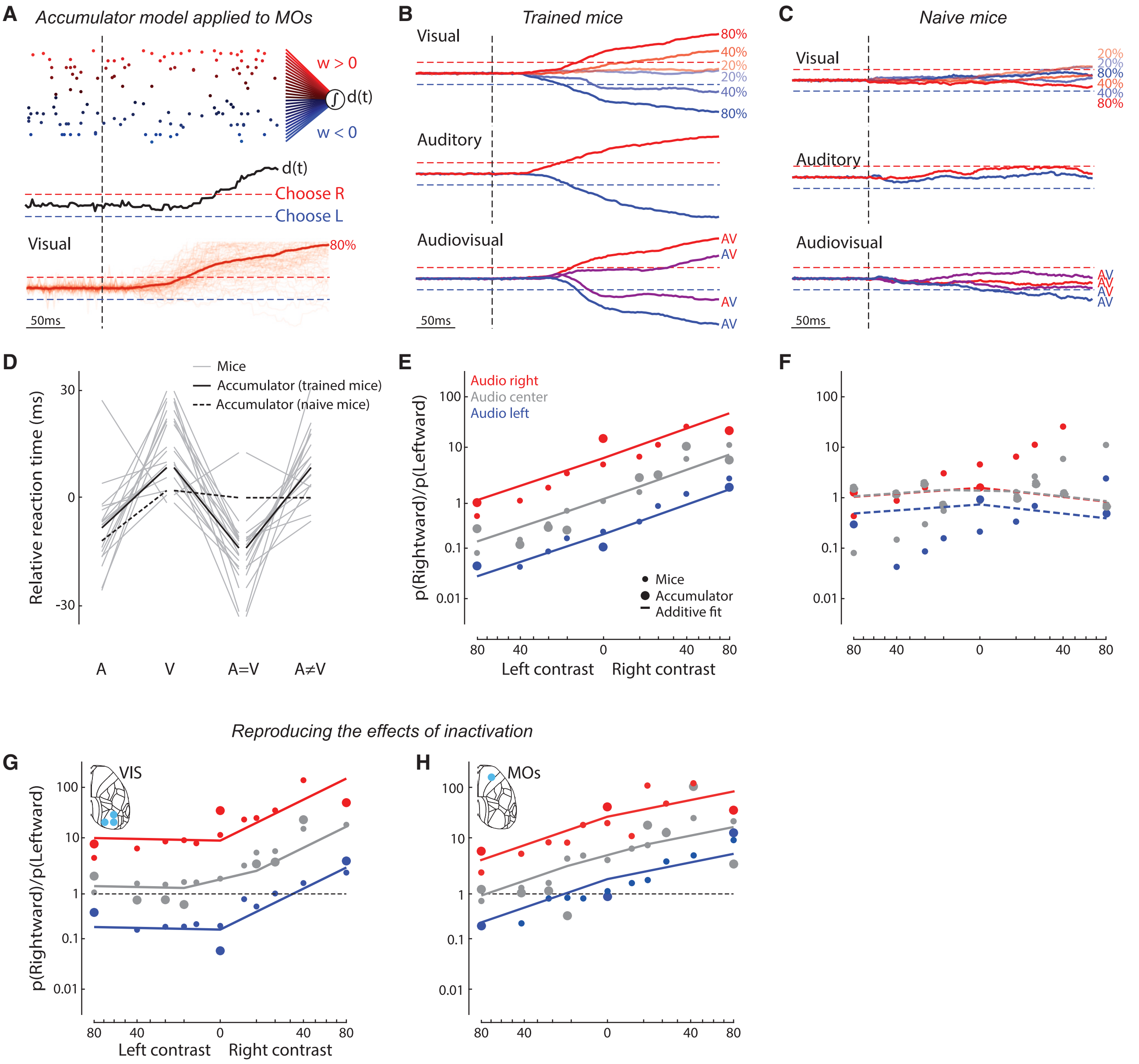

Figure 6. An accumulator applied to MOs activity in trained mice reproduced decisions

(A) Top: population spike train rasters for a single trial, colored according to the fitted weight for that neuron. Red and blue neurons push the decision variable, d_t, toward the rightward or leftward decision boundary. Vertical dashed line: stimulus onset. Population activity was created from passive recording sessions in MOs of trained mice. Middle: evolution of the decision variable over this trial. Red/blue dashed lines: rightward/leftward decision boundaries. Bottom: decision variable trajectory for individual unisensory visual trials with 80% rightward contrast (thin) and their mean (thick).

(B) Mean decision variable trajectory for visual-only (top), auditory-only (middle), and multisensory (bottom) stimulus conditions.

(C) As in (B), but for naive mice.

(D) Median reaction times for different stimulus types, relative to mean across stimulus types, for mouse behavior (gray, n = 17; cf. Figure 1B) and the accumulator model fit to MOs activity in trained and naive mice (solid and dashed black lines).

(E) Mean behavior of the accumulator with input spikes from trained mice (large circles). Small circles represent mouse performance (n = 17; cf. Figure 1G). Solid lines: fit of the additive model to the accumulator model output. The accumulator model fits mouse behavior better than shuffled data ($p < 0.01$, shuffle test, see STAR Methods).

(F) As in (E), but for accumulator with input spikes from naive mice. There is no significant difference between the accumulator model and shuffled data ($p > 0.05$).

(G) Simulation of right visual cortex inactivation, plotted as in (E). Activity of visual-left-preferring cells was reduced by 60%. Small circles: mean behavior from visual-cortex-inactivated mice (5 mice; cf. Figure 2G). The accumulator model fits mouse behavior better than shuffled data ($p < 0.01$).

(legend continued on next page)

DISCUSSION

We found that mice localize stimuli by integrating auditory and visual cues additively and that this additive integration relies on frontal area MOs. Inactivation of frontal cortex impaired audiovisual decisions, especially when the inactivation targeted MOs. Recordings across frontal cortex revealed that MOs has the strongest representations of task variables. Its representations of visual and auditory signals persisted even when mice were not performing the task, but emerged largely after training. MOs neurons combined visual and auditory location signals additively, and an accumulator model applied to MOs activity recorded in passive conditions in trained mice predicts the direction and timing of behavioral responses.

Taken together, our findings implicate MOs as a critical cortical region for integration of evidence from multiple modalities. This is consistent with a general role for rodent MOs in sensorimotor transformations: this frontal region has been linked to multiple functions,[75] including flexible sensory-motor mapping,[76,77] perceptual decision-making,[59,60,78–81] value-based action selection,[82] and exploration-exploitation trade-off in visual and auditory behaviors[58]; furthermore, homologous regions of frontal cortex can encode multisensory information in primates.[39]

Sensory representations in rodent MOs have been seen to evolve with learning in unisensory visual tasks,[83,84] consistent with our observations. Our results suggest that the circuits responsible for multisensory decisions resemble those for unisensory decisions: sensory information relevant for the decision is relayed to frontal cortex, where it is integrated and used to guide action. When mice are trained on a multisensory task, MOs learns to represent the multiple modalities, allowing the stimuli to control choices. The weak but significant MOs representation of auditory stimuli before task training might reflect an innate circuit for orienting towards localized sounds.

The effects of inactivation on responses and reaction times to both modalities were strongest when the laser was aimed at anterior MOs. These inactivations may affect wide regions, over 1 mm from the laser's location.[59,63,64] Nevertheless, if the critical region for multisensory processing were some surface area other than anterior MOs, one would see a stronger effect targeting the laser in that area. It is also possible that targeting MOs inactivates regions below it, such as ACA or ORB. However, electrode recordings revealed that these regions had no neural correlates of upcoming choice and weaker correlates of stimulus location (the difference with ACA did not reach significance). We therefore conclude that MOs is an important center for transforming visual and auditory stimuli into motor actions, operating either alone or in parallel with other circuits. It may be part of a distributed cortical and subcortical circuit for integrating sensory evidence, choosing an action plan, and planning and executing movements.

The circuit for audiovisual integration might include the border region between primary visual region (VISp) and primary auditory region (AUDp) (lateral sensory cortex), where inactivations affected both visual and auditory choices. However, our data cannot distinguish whether this reflects multisensory integration or simply lateral spread of the inactivation to both sensory cortices. If it is multisensory, it plays a different role from MOs: inactivating it had weaker effects (particularly on auditory stimuli, as might be expected if the effect arose from diffusion of light through the brain to underlying auditory cortex) and did not affect reaction time. Our data also cannot speak to the role in audiovisual integration of cortical areas below the surface (such as temporal association areas,[85] entorhinal area, or perirhinal area). However, we can conclude that the role of parietal cortex in this task is purely visual. This might appear to contradict previous work implicating parietal cortex in multisensory integration[4,6,7,18,29–36,86] or showing multisensory activity in primary sensory cortices.[41–50] However, our finding agrees with evidence that parietal neurons can encode multisensory stimuli without being causally involved in a task.[29,51,52,87]

We hypothesize that the causal role of visual and auditory cortices in this task is unimodal and that these cortices relay their unimodal signals to other regions (possibly via unimodal higher sensory areas[88]) where the two information streams are integrated.[86,87,89] We have confirmed this hypothesis for visual cortex but not for auditory cortex. Doing so would require better access to lateralized areas.

An additive integration strategy is optimal when the probability distributions of visual and auditory signals are conditionally independent given the stimulus location,[1] but it may be a useful heuristic[90] in a broader set of circumstances. In fact, in our task independence holds only approximately (see STAR Methods, Figure S8). Nevertheless, additive integration is a simple computation[2] that does not require learning detailed statistics of the sensory world and performs close to the optimum in many situations.

The additive model we observe in mice derives from Bayesian integration, the predominantly accepted integration strategy in humans and other animals.[3–13] However, there is a distinction between the model and some previous work. Typically, previous studies fit psychometric curves based on a cumulative Gaussian function,[3,13] which necessitates using lapse rates.[58] Our approach instead starts with a conditional independence assumption, which implies that psychometric curves are a logistic function applied to a sum of evidence from the two modalities (see STAR Methods, Figure S8). Our model does not speak to the shape or linearity of these evidence functions. We found empirically that power functions of contrast approximate the data well, but this was not a necessary assumption (see STAR Methods, Figure S8).

Our finding of additive integration might appear to contradict observations from an audiovisual detection task, which suggested that mice were auditory dominant.[18,19] However, the discrepancy might arise from differences in the neural representation of stimulus onsets vs. locations. Our task required localization, and the relevant auditory and visual signals combined additively in MOs, with temporal differences that explain the

(H) Simulation of right MOs inactivation, plotted as in (E). Activities of neurons in left and right hemispheres were constrained to have positive and negative weights, and right-hemisphere activity was reduced by 60% before fitting. Small circles: mean behavior from MOs-inactivated mice (5 mice, small circles; cf. Figure 2H). The accumulator model fits mouse behavior better than shuffled data ($p < 0.01$).

CellPress
OPEN ACCESS

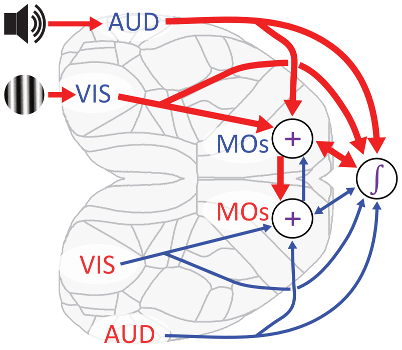

Figure 7. Diagram of hypothesized audiovisual integration pathway through cortex

Our data suggest that visual and auditory unisensory information are conveyed via visual (VIS) and auditory (AUD) sensory cortices to MOs, where a bilateral representation results from interhemispheric connections. A downstream integrator, distributed over multiple brain regions, possibly including MOs itself, accumulates MOs activity, with a biased sampling of neurons responding to contralateral stimuli. An appropriate action is then determined by an integration to bound mechanism. Alternative pathways from visual and auditory cortices appear to be able to compensate for the absence of MOs activity (e.g., during bilateral inactivation).

mice's earlier reactions to auditory stimuli. However, we also saw that neural signals encoding auditory onset were stronger and substantially earlier than neural signals encoding either visual onset or stimulus location from either modality. These strong and early auditory onset signals might dominate behavior in a detection task.[18] In other words, mice might integrate audiovisual signals additively when tasked with localizing a source but be dominated by auditory cues when tasked with detecting the source's presence.

In summary, our data suggest that MOs neurons learn to additively integrate evidence from visual and auditory stimuli, producing a population representation that persists even outside the task and is suitable in the task for guiding a downstream circuit that makes decisions by integration-to-bound. This evidence may be conveyed to MOs via sensory cortices and then fed to downstream circuits that accumulate and threshold activity to select an appropriate action (Figure 7). Based on results in a unisensory task,[95] we suspect the downstream integrator is a loop that includes MOs itself, together with basal ganglia and midbrain. As bilateral MOs inactivation slowed decision-making, but did not otherwise change behavior, we hypothesize that redundant circuitry can compensate for MOs when it is silenced.

The sensory code we observed in MOs has some apparently paradoxical features, but these would not prevent its efficient use by a downstream accumulator. First, a neuron's preference for visual location showed no apparent relation to its preference for auditory location, consistent with reports from multisensory neural populations in primates[6,37] and rats.[29] Such "mixed selectivity" might allow downstream circuits to quickly learn to extract relevant feature combinations.[91–94] Neurons encoding incoherent stimulus locations would not prevent a downstream decision circuit from learning to respond correctly; they could be ignored in the current task, but they would provide flexibility should task demands change. Second, although an approximately equal number of MOs neurons in each hemisphere preferred left and right stimuli of either modality, inactivation of MOs caused a lateralized effect on behavior. This apparent contradiction could be resolved if a specific subset of cortical neurons showed lateral bias[74] or if the downstream decision circuit weighted MOs neurons in a biased manner. Indeed, midbrain neurons encoding choices in a similar task are highly lateralized,[95] and the subcortical circuits connecting MOs to midbrain stay largely within each hemisphere. Indeed, when we constrained the accumulator model so that MOs neurons only contribute to contralateral choices, we reproduced the lateralized effects of MOs inactivation. Whether this downstream bias exists, and whether it depends on specific neural subtypes, is a question for future studies.

STAR★METHODS

Detailed methods are provided in the online version of this paper and include the following:

- KEY RESOURCES TABLE
- RESOURCE AVAILABILITY
  - Lead contact
  - Materials availability
  - Data and code availability
- EXPERIMENTAL MODEL AND STUDY PARTICIPANT DETAILS
  - Mice
- METHOD DETAILS
  - Terminology
  - Surgery
  - Audiovisual behavioral task
  - Optogenetic inactivation
  - Neuropixels recordings
  - Passive stimulus presentation recordings
- QUANTIFICATION AND STATISTICAL ANALYSIS
  - Statistics
  - Behavioral quantification
  - Video motion energy analysis
  - Wheel movement during active vs. passive conditions
  - Psychometric modeling
  - Quantifying effects of optogenetic inactivation on choice
  - Quantifying effects of optogenetic inactivation on model parameters
  - The effect of inactivation on reaction time, fraction of timeout/slow trials, and rightward choices
  - Estimating firing rate
  - Decoding stimuli and choices from population activity
  - Quantifying ramping of choice-related activity in MOs
  - Combined-conditions choice/stimulus probability analysis
  - Modeling neural activity
  - Lateralization of stimulus and movement activity
  - Quantifying stimulus and movement activity as a function of location within MOs
  - Quantifying single-neuron discrimination time
  - Quantifying single-neuron discriminability index

- Accumulator model
- Optimally combining independent visual and auditory signals

SUPPLEMENTAL INFORMATION

Supplemental information can be found online at https://doi.org/10.1016/j.neuron.2023.05.008.

ACKNOWLEDGMENTS

We thank Michael Krumin, Peter Zatka-Haas, Andrew J. Peters, Max Hunter, Flóra Takács, James Chadwick, Paul Johnson, and Ian Macartney for assistance with the experimental setup; Charu Reddy, Laura Funnell, Dylan Rich, Siddharth Kackar, and Hamish Forrest for help with mouse husbandry, training, experimental assistance, and spike sorting; Charu Reddy, Laura Funnell, and Flóra Takács for surgical assistance and optimization; Laura Funnell, Rakesh Raghupathy, David Orme, and Magdalena Robacha for histology processing; and Samuel Picard, Célian Bimbard, and Maxwell Shinn for reading earlier versions of the manuscript. This work was supported by the Wellcome Trust (Sir Henry Wellcome Postdoctoral Fellowship 110120 to P.C. and grants 205093 and 204915 to M.C. and K.D.H.), the European Union's Horizon 2020 research and innovation program (European Research Council grant 694401 to K.D.H. and Marie Sklodowska-Curie fellowship 705391 to P.C.), and the Biotechnology and Biological Sciences Research Council (Responsive mode grant BB/T016639/1 to M.C. and P.C.). T.P.H.S. is supported by the Sainsbury Wellcome Center PhD program. M.C. holds the GlaxoSmithKline/Fight for Sight Chair in Visual Neuroscience.

AUTHOR CONTRIBUTIONS

P.C., M.C., and K.D.H. conceived of and designed the study. P.C. designed the task. P.C. and M.J.W. collected the behavioral data. P.C. collected the neural data. P.C. and T.P.H.S. analyzed data. P.C., K.D.H., and M.C. wrote the manuscript with input from T.P.H.S., and P.C. and K.D.H. wrote the initial draft.

DECLARATION OF INTERESTS

M.C. is a member of *Neuron*'s advisory board.

Received: May 18, 2022
Revised: December 2, 2022
Accepted: May 10, 2023
Published: June 8, 2023

REFERENCES

1. Gold, J.I., and Shadlen, M.N. (2002). Banburismus and the Brain Decoding the Relationship between Sensory Stimuli, Decisions, and Reward. Neuron *36*, 299–308. https://doi.org/10.1016/s0896-6273(02)00971-6.
2. Ma, W.J., Beck, J.M., Latham, P.E., and Pouget, A. (2006). Bayesian inference with probabilistic population codes. Nat. Neurosci. *9*, 1432–1438. https://doi.org/10.1038/nn1790.
3. Ernst, M.O., and Banks, M.S. (2002). Humans integrate visual and haptic information in a statistically optimal fashion. Nature *415*, 429–433. https://doi.org/10.1038/415429a.
4. Angelaki, D.E., Gu, Y., and DeAngelis, G.C. (2009). Multisensory integration: psychophysics, neurophysiology, and computation. Curr. Opin. Neurobiol. *19*, 452–458. https://doi.org/10.1016/j.conb.2009.06.008.
5. Chandrasekaran, C. (2017). Computational principles and models of multisensory integration. Curr. Opin. Neurobiol. *43*, 25–34. https://doi.org/10.1016/j.conb.2016.11.002.
6. Gu, Y., Angelaki, D.E., and DeAngelis, G.C. (2008). Neural correlates of multisensory cue integration in macaque MSTd. Nat. Neurosci. *11*, 1201–1210. https://doi.org/10.1038/nn.2191.
7. Fetsch, C.R., Pouget, A., DeAngelis, G.C., and Angelaki, D.E. (2011). Neural correlates of reliability-based cue weighting during multisensory integration. Nat. Neurosci. *15*, 146–154. https://doi.org/10.1038/nn.2983.
8. Alais, D., and Burr, D. (2004). The Ventriloquist Effect Results from Near-Optimal Bimodal Integration. Curr. Biol. *14*, 257–262. https://doi.org/10.1016/j.cub.2004.01.029.
9. Witten, I.B., and Knudsen, E.I. (2005). Why Seeing Is Believing: Merging Auditory and Visual Worlds. Neuron *48*, 489–496. https://doi.org/10.1016/j.neuron.2005.10.020.
10. Körding, K.P., and Wolpert, D.M. (2004). Bayesian integration in sensorimotor learning. Nature *427*, 244–247. https://doi.org/10.1038/nature02169.
11. Franklin, D.W., and Wolpert, D.M. (2011). Computational Mechanisms of Sensorimotor Control. Neuron *72*, 425–442. https://doi.org/10.1016/j.neuron.2011.10.006.
12. Raposo, D., Sheppard, J.P., Schrater, P.R., and Churchland, A.K. (2012). Multisensory Decision-Making in Rats and Humans. J. Neurosci. *32*, 3726–3735. https://doi.org/10.1523/jneurosci.4998-11.2012.
13. Sheppard, J.P., Raposo, D., and Churchland, A.K. (2013). Dynamic weighting of multisensory stimuli shapes decision-making in rats and humans. J. Vis. *13*, 4. https://doi.org/10.1167/13.6.4.
14. Colavita, F.B. (1974). Human sensory dominance. Percept. Psychophys. *16*, 409–412. https://doi.org/10.3758/bf03203962.
15. Sinnett, S., Spence, C., and Soto-Faraco, S. (2007). Visual dominance and attention: The Colavita effect revisited. Percept. Psychophys. *69*, 673–686. https://doi.org/10.3758/bf03193770.
16. Battaglia, P.W., Jacobs, R.A., and Aslin, R.N. (2003). Bayesian integration of visual and auditory signals for spatial localization. J Opt Soc Am *20*, 1391–1397. https://doi.org/10.1364/josaa.20.001391.
17. Fetsch, C.R., Turner, A.H., DeAngelis, G.C., and Angelaki, D.E. (2009). Dynamic Reweighting of Visual and Vestibular Cues during Self-Motion Perception. J. Neurosci. *29*, 15601–15612. https://doi.org/10.1523/jneurosci.2574-09.2009.
18. Song, Y.-H., Kim, J.-H., Jeong, H.-W., Choi, I., Jeong, D., Kim, K., and Lee, S.-H. (2017). A Neural Circuit for Auditory Dominance over Visual Perception. Neuron *93*, 940–954.e6. https://doi.org/10.1016/j.neuron.2017.01.006.
19. Lohuis, M.N.O., Marchesi, P., Olcese, U., and Pennartz, C. (2022). Triple dissociation of visual, auditory and motor processing in primary visual cortex. Preprint at bioRxiv. https://doi.org/10.1101/2022.06.29.498156.
20. Gharaei, S., Arabzadeh, E., and Solomon, S.G. (2018). Integration of visual and whisker signals in rat superior colliculus. Sci. Rep. *8*, 16445. https://doi.org/10.1038/s41598-018-34661-8.
21. Costa, M., Piché, M., Lepore, F., and Guillemot, J.-P. (2016). Age-related audiovisual interactions in the superior colliculus of the rat. Neuroscience *320*, 19–29. https://doi.org/10.1016/j.neuroscience.2016.01.058.
22. King, A.J., and Palmer, A.R. (1985). Integration of visual and auditory information in bimodal neurones in the guinea-pig superior colliculus. Exp. Brain Res. *60*, 492–500. https://doi.org/10.1007/bf00236934.
23. Stein, B.E., and Stanford, T.R. (2008). Multisensory integration: current issues from the perspective of the single neuron. Nat. Rev. Neurosci. *9*, 255–266. https://doi.org/10.1038/nrn2331.
24. Meredith, M.A., and Stein, B.E. (1986). Visual, auditory, and somatosensory convergence on cells in superior colliculus results in multisensory integration. J. Neurophysiol. *56*, 640–662. https://doi.org/10.1152/jn.1986.56.3.640.
25. Lohse, M., Dahmen, J.C., Bajo, V.M., and King, A.J. (2021). Subcortical Circuits Mediate Communication Between Primary Sensory Cortical Areas in Mice. Nature Comms *12*, 3916. https://doi.org/10.1038/s41467-021-24200-x.

CellPress
OPEN ACCESS

26. Bieler, M., Xu, X., Marquardt, A., and Hanganu-Opatz, I.L. (2018). Multisensory integration in rodent tactile but not visual thalamus. Sci. Rep. *8*, 15684. https://doi.org/10.1038/s41598-018-33815-y.

27. Chou, X.L., Fang, Q., Yan, L., Zhong, W., Peng, B., Li, H., Wei, J., Tao, H.W., and Zhang, L.I. (2020). Contextual and cross-modality modulation of auditory cortical processing through pulvinar mediated suppression. Elife *9*, e54157. https://doi.org/10.7554/elife.54157.

28. Komura, Y., Tamura, R., Uwano, T., Nishijo, H., and Ono, T. (2005). Auditory thalamus integrates visual inputs into behavioral gains. Nat. Neurosci. *8*, 1203–1209. https://doi.org/10.1038/nn1528.

29. Raposo, D., Kaufman, M.T., and Churchland, A.K. (2014). A category-free neural population supports evolving demands during decision-making. Nat. Neurosci. *17*, 1784–1792. https://doi.org/10.1038/nn.3865.

30. Lippert, M.T., Takagaki, K., Kayser, C., and Ohl, F.W. (2013). Asymmetric multisensory interactions of visual and somatosensory responses in a region of the rat parietal cortex. PLoS One *8*, e63631. https://doi.org/10.1371/journal.pone.0063631.

31. Nikbakht, N., Tafreshiha, A., Zoccolan, D., and Diamond, M.E. (2018). Supralinear and Supramodal Integration of Visual and Tactile Signals in Rats: Psychophysics and Neuronal Mechanisms. Neuron *97*, 626–639.e8. https://doi.org/10.1016/j.neuron.2018.01.003.

32. Ohshiro, T., Angelaki, D.E., and DeAngelis, G.C. (2011). A normalization model of multisensory integration. Nat. Neurosci. *14*, 775–782. https://doi.org/10.1038/nn.2815.

33. Khandhadia, A.P., Murphy, A.P., Romanski, L.M., Bizley, J.K., and Leopold, D.A. (2021). Audiovisual integration in macaque face patch neurons. Curr. Biol. *31*, 1826–1835.e3. https://doi.org/10.1016/j.cub.2021.01.102.

34. Avillac, M., Ben Hamed, S., and Duhamel, J.-R. (2007). Multisensory Integration in the Ventral Intraparietal Area of the Macaque Monkey. J. Neurosci. *27*, 1922–1932. https://doi.org/10.1523/jneurosci.2646-06.2007.

35. Seilheimer, R.L., Rosenberg, A., and Angelaki, D.E. (2014). Models and processes of multisensory cue combination. Curr. Opin. Neurobiol. *25*, 38–46. https://doi.org/10.1016/j.conb.2013.11.008.

36. Avillac, M., Denève, S., Olivier, E., Pouget, A., and Duhamel, J.-R. (2005). Reference frames for representing visual and tactile locations in parietal cortex. Nat. Neurosci. *8*, 941–949. https://doi.org/10.1038/nn1480.

37. Hou, H., Zheng, Q., Zhao, Y., Pouget, A., and Gu, Y. (2019). Neural Correlates of Optimal Multisensory Decision Making under Time-Varying Reliabilities with an Invariant Linear Probabilistic Population Code. Neuron *104*, 1010–1021.e10. https://doi.org/10.1016/j.neuron.2019.08.038.

38. Zheng, Q., Zhou, L., and Gu, Y. (2021). Temporal synchrony effects of optic flow and vestibular inputs on multisensory heading perception. Cell Rep. *37*, 109999. https://doi.org/10.1016/j.celrep.2021.109999.

39. Gu, Y., Cheng, Z., Yang, L., DeAngelis, G.C., and Angelaki, D.E. (2016). Multisensory Convergence of Visual and Vestibular Heading Cues in the Pursuit Area of the Frontal Eye Field. Cereb. Cortex *26*, 3785–3801. https://doi.org/10.1093/cercor/bhv183.

40. Bimbard, C., Sit, T.P.H., Lebedeva, A., Reddy, C.B., Harris, K.D., and Carandini, M. (2023). Behavioral origin of sound-evoked activity in mouse visual cortex. Nat. Neurosci. *26*, 251–258. https://doi.org/10.1038/s41593-022-01227-x.

41. Iurilli, G., Ghezzi, D., Olcese, U., Lassi, G., Nazzaro, C., Tonini, R., Tucci, V., Benfenati, F., and Medini, P. (2012). Sound-Driven Synaptic Inhibition in Primary Visual Cortex. Neuron *73*, 814–828. https://doi.org/10.1016/j.neuron.2011.12.026.

42. Meijer, G.T., Montijn, J.S., Pennartz, C.M.A., and Lansink, C.S. (2017). Audiovisual Modulation in Mouse Primary Visual Cortex Depends on Cross-Modal Stimulus Configuration and Congruency. J. Neurosci. *37*, 8783–8796. https://doi.org/10.1523/JNEUROSCI.0468-17.2017.

43. Ibrahim, L.A., Mesik, L., Ji, X.Y., Fang, Q., Li, H.F., Li, Y.T., Zingg, B., Zhang, L.I., and Tao, H.W. (2016). Cross-Modality Sharpening of Visual Cortical Processing through Layer-1-Mediated Inhibition and Disinhibition. Neuron *89*, 1031–1045. https://doi.org/10.1016/j.neuron.2016.01.027.

44. Bizley, J.K., and King, A.J. (2009). Visual influences on ferret auditory cortex. Hear. Res. *258*, 55–63. https://doi.org/10.1016/j.heares.2009.06.017.

45. Bizley, J.K., and King, A.J. (2008). Visual–auditory spatial processing in auditory cortical neurons. Brain Res. *1242*, 24–36. https://doi.org/10.1016/j.brainres.2008.02.087.

46. Atilgan, H., Town, S.M., Wood, K.C., Jones, G.P., Maddox, R.K., Lee, A.K.C., and Bizley, J.K. (2018). Integration of Visual Information in Auditory Cortex Promotes Auditory Scene Analysis through Multisensory Binding. Neuron *97*, 640–655.e4. https://doi.org/10.1016/j.neuron.2017.12.034.

47. Ghazanfar, A.A., and Schroeder, C.E. (2006). Is neocortex essentially multisensory? Trends Cogn. Sci. *10*, 278–285. https://doi.org/10.1016/j.tics.2006.04.008.

48. Driver, J., and Noesselt, T. (2008). Multisensory Interplay Reveals Crossmodal Influences on 'Sensory-Specific' Brain Regions, Neural Responses, and Judgments. Neuron *57*, 11–23. https://doi.org/10.1016/j.neuron.2007.12.013.

49. Meredith, M.A., and Allman, B.L. (2015). Single-unit analysis of somatosensory processing in the core auditory cortex of hearing ferrets. Eur. J. Neurosci. *41*, 686–698. https://doi.org/10.1111/ejn.12828.

50. Kayser, C., Petkov, C.I., and Logothetis, N.K. (2008). Visual Modulation of Neurons in Auditory Cortex. Cereb. Cortex *18*, 1560–1574. https://doi.org/10.1093/cercor/bhm187.

51. Gu, Y., DeAngelis, G.C., and Angelaki, D.E. (2012). Causal Links between Dorsal Medial Superior Temporal Area Neurons and Multisensory Heading Perception. J. Neurosci. *32*, 2299–2313. https://doi.org/10.1523/jneurosci.5154-11.2012.

52. Licata, A.M., Kaufman, M.T., Raposo, D., Ryan, M.B., Sheppard, J.P., and Churchland, A.K. (2017). Posterior Parietal Cortex Guides Visual Decisions in Rats. J. Neurosci. *37*, 4954–4966. https://doi.org/10.1523/jneurosci.0105-17.2017.

53. Burgess, C.P., Lak, A., Steinmetz, N.A., Zatka-Haas, P., Bai Reddy, C., Jacobs, E.A.K., Linden, J.F., Paton, J.J., Ranson, A., Schröder, S., et al. (2017). High-Yield Methods for Accurate Two-Alternative Visual Psychophysics in Head-Fixed Mice. Cell Rep. *20*, 2513–2524. https://doi.org/10.1016/j.celrep.2017.08.047.

54. Meijer, G.T., Pie, J.L., Dolman, T.L., Pennartz, C.M.A., and Lansink, C.S. (2018). Audiovisual Integration Enhances Stimulus Detection Performance in Mice. Front. Behav. Neurosci. *12*, 231. https://doi.org/10.3389/fnbeh.2018.00231.

55. Hammond-Kenny, A., Bajo, V.M., King, A.J., and Nodal, F.R. (2017). Behavioural benefits of multisensory processing in ferrets. Eur. J. Neurosci. *45*, 278–289. https://doi.org/10.1111/ejn.13440.

56. Raab, D.H. (1962). Division of psychology: Statistical facilitation of simple reaction times. Trans. N. Y. Acad. Sci. *24*, 574–590. https://doi.org/10.1111/j.2164-0947.1962.tb01433.x.

57. Stein, B.E., Meredith, M.A., Huneycutt, W.S., and McDade, L. (1989). Behavioral Indices of Multisensory Integration: Orientation to Visual Cues is Affected by Auditory Stimuli. J. Cogn. Neurosci. *1*, 12–24. https://doi.org/10.1162/jocn.1989.1.1.12.

58. Pisupati, S., Chartarifsky-Lynn, L., Khanal, A., and Churchland, A.K. (2021). Lapses in perceptual decisions reflect exploration. Elife *10*, e55490. https://doi.org/10.7554/elife.55490.

59. Zatka-Haas, P., Steinmetz, N.A., Carandini, M., and Harris, K.D. (2021). Sensory coding and the causal impact of mouse cortex in a visual decision. Elife *10*, e63163. https://doi.org/10.7554/elife.63163.

60. Guo, Z.V., Li, N., Huber, D., Ophir, E., Gutnisky, D., Ting, J.T., Feng, G., and Svoboda, K. (2014). Flow of cortical activity underlying a tactile decision in mice. Neuron *81*, 179–194. https://doi.org/10.1016/j.neuron.2013.10.020.

61. Cardin, J.A., Carlén, M., Meletis, K., Knoblich, U., Zhang, F., Deisseroth, K., Tsai, L.-H., and Moore, C.I. (2009). Driving fast-spiking cells induces gamma rhythm and controls sensory responses. Nature *459*, 663–667. https://doi.org/10.1038/nature08002.

62. Olsen, S.R., Bortone, D.S., Adesnik, H., and Scanziani, M. (2012). Gain control by layer six in cortical circuits of vision. Nature *483*, 47–52. https://doi.org/10.1038/nature10835.

63. Li, N., Chen, S., Guo, Z.V., Chen, H., Huo, Y., Inagaki, H.K., Chen, G., Davis, C., Hansel, D., Guo, C., and Svoboda, K. (2019). Spatiotemporal constraints on optogenetic inactivation in cortical circuits. Elife *8*, e48622. https://doi.org/10.7554/elife.48622.

64. Hao, Y., Thomas, A.M., and Li, N. (2021). Fully autonomous mouse behavioral and optogenetic experiments in home-cage. Elife *10*, e66112. https://doi.org/10.7554/elife.66112.

65. Glickfeld, L.L., Histed, M.H., and Maunsell, J.H.R. (2013). Mouse Primary Visual Cortex Is Used to Detect Both Orientation and Contrast Changes. J. Neurosci. *33*, 19416–19422. https://doi.org/10.1523/jneurosci.3560-13.2013.

66. Jenkins, W.M., and Merzenich, M.M. (1984). Role of cat primary auditory cortex for sound-localization behavior. J. Neurophysiol. *52*, 819–847. https://doi.org/10.1152/jn.1984.52.5.819.

67. Wang, Q., Ding, S.-L., Li, Y., Royall, J., Feng, D., Lesnar, P., Graddis, N., Naeemi, M., Facer, B., Ho, A., et al. (2020). The Allen Mouse Brain Common Coordinate Framework: A 3D Reference Atlas. Cell *181*, 936–953.e20. https://doi.org/10.1016/j.cell.2020.04.007.

68. Stringer, C., Pachitariu, M., Steinmetz, N., Reddy, C.B., Carandini, M., and Harris, K.D. (2019). Spontaneous behaviors drive multidimensional, brainwide activity. Science *364*, 255. https://doi.org/10.1126/science.aav7893.

69. Musall, S., Kaufman, M.T., Juavinett, A.L., Gluf, S., and Churchland, A.K. (2019). Single-trial neural dynamics are dominated by richly varied movements. Nat. Neurosci. *22*, 1677–1686. https://doi.org/10.1038/s41593-019-0502-4.

70. Park, I.M., Meister, M.L.R., Huk, A.C., and Pillow, J.W. (2014). Encoding and decoding in parietal cortex during sensorimotor decision-making. Nat. Neurosci. *17*, 1395–1403. https://doi.org/10.1038/nn.3800.

71. Ratcliff, R. (1978). A theory of memory retrieval. Psychol. Rev. *85*, 59–108. https://doi.org/10.1037/0033-295x.85.2.59.

72. Gold, J.I., and Shadlen, M.N. (2007). The Neural Basis of Decision Making. Annu. Rev. Neurosci. *30*, 535–574. https://doi.org/10.1146/annurev.neuro.29.051605.113038.

73. Drugowitsch, J., DeAngelis, G.C., Klier, E.M., Angelaki, D.E., and Pouget, A. (2014). Optimal multisensory decision-making in a reaction-time task. Elife *3*, e03005. https://doi.org/10.7554/elife.03005.

74. Li, N., Chen, T.-W., Guo, Z.V., Gerfen, C.R., and Svoboda, K. (2015). A motor cortex circuit for motor planning and movement. Nature *519*, 51–56. https://doi.org/10.1038/nature14178.

75. Ebbesen, C.L., Insanally, M.N., Kopec, C.D., Murakami, M., Saiki, A., and Erlich, J.C. (2018). More than Just a “Motor”: Recent Surprises from the Frontal Cortex. J. Neurosci. *38*, 9402–9413. https://doi.org/10.1523/jneurosci.1671-18.2018.

76. Barthas, F., and Kwan, A.C. (2017). Secondary Motor Cortex: Where ‘Sensory’ Meets ‘Motor’ in the Rodent Frontal Cortex. Trends Neurosci. *40*, 181–193. https://doi.org/10.1016/j.tins.2016.11.006.

77. Duan, C.A., Erlich, J.C., and Brody, C.D. (2015). Requirement of Prefrontal and Midbrain Regions for Rapid Executive Control of Behavior in the Rat. Neuron *86*, 1491–1503. https://doi.org/10.1016/j.neuron.2015.05.042.

78. Hanks, T.D., Kopec, C.D., Brunton, B.W., Duan, C.A., Erlich, J.C., and Brody, C.D. (2015). Distinct relationships of parietal and prefrontal cortices to evidence accumulation. Nature *520*, 220–223. https://doi.org/10.1038/nature14066.

79. Erlich, J.C., Brunton, B.W., Duan, C.A., Hanks, T.D., and Brody, C.D. (2015). Distinct effects of prefrontal and parietal cortex inactivations on an accumulation of evidence task in the rat. Elife *4*, e05457. https://doi.org/10.7554/elife.05457.

80. Erlich, J.C., Bialek, M., and Brody, C.D. (2011). A Cortical Substrate for Memory-Guided Orienting in the Rat. Neuron *72*, 330–343. https://doi.org/10.1016/j.neuron.2011.07.010.

81. Insanally, M.N., Carcea, I., Field, R.E., Rodgers, C.C., DePasquale, B., Rajan, K., DeWeese, M.R., Albanna, B.F., and Froemke, R.C. (2019). Spike-timing-dependent ensemble encoding by non-classically responsive cortical neurons. Elife *8*, e42409. https://doi.org/10.7554/elife.42409.

82. Sul, J.H., Jo, S., Lee, D., and Jung, M.W. (2011). Role of rodent secondary motor cortex in value-based action selection. Nat. Neurosci. *14*, 1202–1208. https://doi.org/10.1038/nn.2881.

83. Orsolic, I., Rio, M., Mrsic-Flogel, T., and Znamenskiy, P. (2021). Mesoscale cortical dynamics reflect the interaction of sensory evidence and temporal expectation during perceptual decision-making. Neuron *109*, 1861–1875. https://doi.org/10.1016/j.neuron.2021.03.031.

84. Peters, A.J., Marica, A.-M., Fabre, J.M.J., Harris, K.D., and Carandini, M. (2022). Visuomotor learning promotes visually evoked activity in the medial prefrontal cortex. Cell Rep. *41*, 111487. https://doi.org/10.1016/j.celrep.2022.111487.

85. Dahl, C.D., Logothetis, N.K., and Kayser, C. (2009). Spatial organization of multisensory responses in temporal association cortex. J. Neurosci. *29*, 11924–11932. https://doi.org/10.1523/jneurosci.3437-09.2009.

86. Rohe, T., and Noppeney, U. (2016). Distinct Computational Principles Govern Multisensory Integration in Primary Sensory and Association Cortices. Curr. Biol. *26*, 509–514. https://doi.org/10.1016/j.cub.2015.12.056.

87. Cao, Y., Summerfield, C., Park, H., Giordano, B.L., and Kayser, C. (2019). Causal Inference in the Multisensory Brain. Neuron *102*, 1076–1087.e8. https://doi.org/10.1016/j.neuron.2019.03.043.

88. Oh, S.W., Harris, J.A., Ng, L., Winslow, B., Cain, N., Mihalas, S., Wang, Q., Lau, C., Kuan, L., Henry, A.M., et al. (2014). A mesoscale connectome of the mouse brain. Nature *508*, 207–214. https://doi.org/10.1038/nature13186.

89. Jones, E.G., and Powell, T.P. (1970). An Anatomical Study of Converging Sensory Pathways Within the Cerebral Cortex of the Monkey. Brain *93*, 793–820. https://doi.org/10.1093/brain/93.4.793.

90. Gardner, J.L. (2019). Optimality and heuristics in perceptual neuroscience. Nat. Neurosci. *22*, 514–523. https://doi.org/10.1038/s41593-019-0340-4.

91. Maass, W., Natschläger, T., and Markram, H. (2002). Real-Time Computing Without Stable States: A New Framework for Neural Computation Based on Perturbations. Neural Comput. *14*, 2531–2560. https://doi.org/10.1162/089976602760407955.

92. Caron, S.J.C., Ruta, V., Abbott, L.F., and Axel, R. (2013). Random convergence of olfactory inputs in the Drosophila mushroom body. Nature *497*, 113–117. https://doi.org/10.1038/nature12063.

93. Rigotti, M., Barak, O., Warden, M.R., Wang, X.-J., Daw, N.D., Miller, E.K., and Fusi, S. (2013). The importance of mixed selectivity in complex cognitive tasks. Nature *497*, 585–590. https://doi.org/10.1038/nature12160.

94. Fusi, S., Miller, E.K., and Rigotti, M. (2016). Why neurons mix: high dimensionality for higher cognition. Curr. Opin. Neurobiol. *37*, 66–74. https://doi.org/10.1016/j.conb.2016.01.010.

95. Steinmetz, N.A., Zatka-Haas, P., Carandini, M., and Harris, K.D. (2019). Distributed coding of choice, action and engagement across the mouse brain. Nature *576*, 266–273. https://doi.org/10.1038/s41586-019-1787-x.

CellPress
OPEN ACCESS

96. Bhagat, J., Wells, M.J., Harris, K.D., Carandini, M., and Burgess, C.P. (2020). Rigbox: An Open-Source Toolbox for Probing Neurons and Behavior. Eneuro *7*. ENEURO.0406-19.2020. https://doi.org/10.1523/eneuro.0406-19.2020.

97. Pachitariu, M., Steinmetz, N.A., Kadir, S.N., and Carandini, M. (2016). Kilosort: realtime spike-sorting for extracellular electrophysiology with hundreds of channels. Preprint at bioRxiv. https://doi.org/10.1101/061481.

98. Steinmetz, N.A., Buetfering, C., Lecoq, J., Lee, C.R., Peters, A.J., Jacobs, E.A.K., Coen, P., Ollerenshaw, D.R., Valley, M.T., de Vries, S.E.J., et al. (2017). Aberrant Cortical Activity in Multiple GCaMP6-Expressing Transgenic Mouse Lines. Eneuro *4*. ENEURO.0207-17.2017. https://doi.org/10.1523/eneuro.0207-17.2017.

99. Jun, J.J., Steinmetz, N.A., Siegle, J.H., Denman, D.J., Bauza, M., Barbarits, B., Lee, A.K., Anastassiou, C.A., Andrei, A., Aydın, Ç., et al. (2017). Fully integrated silicon probes for high-density recording of neural activity. Nature *551*, 232–236. https://doi.org/10.1038/nature24636.

100. Siegle, J.H., López, A.C., Patel, Y.A., Abramov, K., Ohayon, S., and Voigts, J. (2017). Open Ephys: an open-source, plugin-based platform for multichannel electrophysiology. J. Neural. Eng. *14*, 045003. https://doi.org/10.1088/1741-2552/aa5eea.

101. Rossant, C., Kadir, S.N., Goodman, D.F.M., Schulman, J., Hunter, M.L.D., Saleem, A.B., Grosmark, A., Belluscio, M., Denfield, G.H., Ecker, A.S., et al. (2016). Spike sorting for large, dense electrode arrays. Nat. Neurosci. *19*, 634–641. https://doi.org/10.1038/nn.4268.

102. Peters, A.J., Fabre, J.M.J., Steinmetz, N.A., Harris, K.D., and Carandini, M. (2021). Striatal activity topographically reflects cortical activity. Nature *591*, 420–425. https://doi.org/10.1038/s41586-020-03166-8.

103. Steinmetz, N.A., Aydin, C., Lebedeva, A., Okun, M., Pachitariu, M., Bauza, M., Beau, M., Bhagat, J., Böhm, C., Broux, M., et al. (2021). Neuropixels 2.0: A miniaturized high-density probe for stable, long-term brain recordings. Science *372*, eabf4588. https://doi.org/10.1126/science.abf4588.

104. Kingma, D.P., and Ba, J. (2014). Adam: A Method for Stochastic Optimization. Preprint at arxiv. https://doi.org/10.48550/arXiv.1412.6980.

105. Frostig, R., Johnson, M.J., and Leary, C. (2018). Compiling Machine Learning Programs via High-Level Tracing (1st SysML Conference).

STAR★METHODS

KEY RESOURCES TABLE

REAGENT or RESOURCE	SOURCE	IDENTIFIER
**Deposited data**		
Mouse Common Coordinate Framework	Allen Institute	https://doi.org/10.1016/j.cell.2020.04.007
Sorted spikes, behavioral data	This paper	https://github.com/pipcoen/2023_CoenSit
**Experimental models: Organisms/strains**		
Mouse: C57BL/6J	Jackson Labs	RRID: IMSR_JAX:000664
Mouse: Ai32	Jackson Labs	RRID: IMSR_JAX:012569
Mouse: PV-Cre	Jackson Labs	RRID: IMSR_JAX:008069
**Software and algorithms**		
MATLAB version 2022b	The MathWorks, USA	www.mathworks.com
Rigbox/Signals	Bhagat et al., 2020[96]	https://github.com/cortex-lab/Rigbox
kilosort2	Pachitariu et al., 2016[97]	https://github.com/MouseLand/Kilosort/tree/v2.0
Phy	Cyrille Rossant	https://github.com/cortex-lab/phy
Motion analysis	Stringer et al., 2019[68]	https://github.com/MouseLand/facemap/tree/v1.0.0
Python version 3.7.11	Python Software Foundation	https://www.python.org
Python and MATLAB code to analyze data	This paper	https://github.com/pipcoen/2023_CoenSit, https://doi.org/10.5281/zenodo.7892397
**Other**		
Video displays	Adafruit	LP097QX1
Fresnel lenses	Wuxi Bohai Optics, China	BHPA220-2-5
Diffusing film	Window Film Company	Frostbite
Neuropixels probes	imec, Belgium	www.neuropixels.org

RESOURCE AVAILABILITY

Lead contact

Further information and requests for resources should be directed to the lead contact, Philip Coen (p.coen@ucl.ac.uk).

Materials availability

This study did not generate new unique reagents.

Data and code availability

- The processed data analyzed in the current study are online (see https://github.com/pipcoen/2023_CoenSit for link). Raw data are available from the lead contact on reasonable request.
- The code used in the current study is available online (https://github.com/pipcoen/2023_CoenSit, https://doi.org/10.5281/zenodo.7892397).
- Any additional information required to reanalyze the data reported in this paper is available from the lead contact on reasonable request.

EXPERIMENTAL MODEL AND STUDY PARTICIPANT DETAILS

Mice

Experimental procedures were conducted according to the UK Animals Scientific Procedures Act (1986) and under personal and project licenses released by the Home Office following appropriate ethics review.

Experiments were performed on 3 male and 15 female mice, aged between 9 and 21 weeks at time of surgery. The sex of the mice used did not influence the results. For all experiments, we used either transgenic mice expressing ChR2 in Parvalbumin-positive

inhibitory interneurons (Ai32 [Jax #012569, RRID:IMSR_JAX: 012569] x PV-Cre [Jax #008069, RRID:IMSR_JAX: 008069]) or wild type C57BL/6J [Jackson Labs, RRID:IMSR_JAX:000664]. 17 mice contribute to behavioral data (Figure 1), 5 mice contribute to optogenetic inactivation data (Figure 2), and 6/4 mice contribute to electrophysiological recordings in trained/naive mice (Figures 3, 4, 5, and 6). Behavioral data (Figure 1) comprised both sessions without any optogenetic inactivation and non-inactivation trials within optogenetic experiments. Mice were either single-housed or co-housed in individually ventilated cages at the Biological Services Unit in University College London.

METHOD DETAILS

Terminology

Here, we define some terms used throughout the methods and manuscript. A "stimulus condition" refers to a particular combination of auditory and visual stimuli; for example, a visual stimulus of 40% contrast on the left and an auditory stimulus presented on the right. A "stimulus type" refers to a category that may comprise several stimulus conditions. We define five different stimulus types: unisensory auditory, unisensory visual, coherent, conflict, and neutral. "Unisensory auditory" trials are when an auditory stimulus is presented on the left or right, and contrast is zero (gray screen). "Unisensory visual" trials are when a stimulus of any contrast greater than zero is presented on the left or right, and the auditory stimulus is presented in the center (during behavior) or is absent (during passive conditions). "Coherent" trials are when a visual stimulus with non-zero contrast is presented on the same side as an auditory stimulus. "Conflict" trials are when a visual stimulus with non-zero contrast is presented on a different side from an auditory stimulus. "Neutral" trials are when the visual contrast is zero and the auditory stimulus is presented in the center. We refer to a single experimental recording (whether purely behavior, or combined with optogenetic inactivation or electrophysiology) as a "session." Sessions can vary in duration and number of trials. Throughout the manuscript, "t test" indicates a two-sided t test unless otherwise specified. When referring to inactivation we use the term "site" to refer to a single target location (of which there were 52 in total) on dorsal cortex and "region" to refer to a collection of sites (3 sites in each case) in visual, lateral, somatosensory, or frontal cortex.

Surgery

A brief (around 1 h) initial surgery was performed under isoflurane (1–3% in O2) anesthesia to implant a steel headplate (approximately 25 × 3 × 0.5 mm, 1 g) and, in most cases, a 3D-printed recording chamber. The chamber comprised two pieces of opaque polylactic acid which combined to expose an area approximately 4 mm anterior to 5 mm posterior to bregma, and 5 mm left to 5 mm right, narrowing near the eyes. The implantation method largely followed established methods[60] and has been previously described.[98] In brief, the dorsal surface of the skull was cleared of skin and periosteum. The lower part of the chamber was attached to the skull with cyanoacrylate (VetBond; World Precision Instruments) and the gaps between chamber skull were filled with L-type radiopaque polymer (Super-Bond C&B, Sun Medical). A thin layer of cyanoacrylate was applied to the skull inside the cone and allowed to dry. Thin layers of UV-curing optical glue (Norland Optical Adhesives #81, Norland Products) were applied inside the cone and cured until the exposed skull was covered. The head plate was attached to the skull over the interparietal bone with Super-Bond polymer. The upper part of the cone was then affixed to the headplate and lower cone with a further application of polymer. After recovery, mice were treated with carprofen for three days, then acclimated to handling and head-fixation before training.

Audiovisual behavioral task

The two-alternative forced choice task design was an extension of a previously described visual task.[53] It was programmed in Signals, part of the Rigbox MATLAB package.[96] Mice sat on a plastic apparatus with their forepaws on a rigid, rubber Lego wheel affixed to a rotary encoder (Kubler 05.2400.1122.0360). A plastic tube for delivery of water rewards was placed near the subject's mouth.

Visual stimuli were presented using three computer screens (Adafruit, LP097QX1), arranged at right angles to cover ± 135° azimuth and ± 45° elevation, where 0° is directly in front of the subject. Each screen was roughly 11 cm from the mouse's eyes at its nearest point and refreshed at 60 Hz. Intensity values were linearized[53] with a photodiode (PDA25K2, Thor labs). The screens were fitted with Fresnel lenses (Wuxi Bohai Optics, BHPA220-2-5) to ameliorate reductions in luminance and contrast at larger viewing angles, and these lenses were coated with scattering window film ('frostbite', The Window Film Company) to reduce reflections. Visual stimuli were flashing vertical Gabors presented with a 9° Gaussian window, spatial frequency 1/15 cycles per degree, vertical position 0° (i.e. level with the mouse) and phase randomly selected on each trial. Stimuli flashed at a constant rate of 8Hz, with each presentation lasting for ~ 50 ms (with some jitter due to screen refresh times).

Auditory stimuli were presented using an array of 7 speakers (102-1299-ND, Digikey), arranged below the screens at 30° azimuthal intervals from −90° to +90° (where −90°/+90° is directly to the left/right of the subject). Speakers were driven with an internal sound card (STRIX SOAR, ASUS) and custom 7-channel amplifier (http://maxhunter.me/portfolio/7champ/). The frequency response of each speaker was individually estimated *in situ* with white noise playback recorded with a calibrated microphone (GRAS 40BF 1/4″ Ext. Polarized Free-field Microphone). For each speaker, a compensating filter was generated to flatten the frequency response using the Signal Processing Toolbox in MATLAB. Throughout all sessions, we presented white noise at ~ 50 dbSPL to equalize background noise between different training and experimental rigs.

Auditory stimuli were 50 ms pulses of filtered pink noise (8–16kHz, 75–80 dbSPL), with 16ms sinusoidal onset/offset ramps. To ensure mice did not entrain to any residual difference in the frequency response of the speakers, auditory stimuli were further

modulated on each trial by a filter selected randomly from 100 pre-generated options, which randomly amplified and suppressed different frequency components within the 8–16kHz range. As with visual stimuli, sound pulses were presented at a rate of 8Hz. On multisensory trials, the modulation of visual and auditory stimuli was synchronized, but software limitations and hardware jitter resulted in visual stimuli preceding auditory stimuli by 10 ± 12 ms (mean ± SD).

A trial was initiated after the subject held the wheel still for a short quiescent period (duration uniformly distributed between 0.1 and 0.25 s on each trial; Figure 1A). Mice were randomly presented with different combinations of visual and auditory stimuli (Figure S1A). Visual stimuli varied in azimuthal position (−60° or +60°) and contrast (0%, 10%, 20%, 40%, and 80%, and also 6% in a subset of mice). On unisensory auditory trials, contrast was zero (gray screen). Auditory stimuli varied only in azimuthal position: −60°, 0°, or +60°; on unisensory visual trials, auditory stimuli were positioned at 0°. A small number of "neutral trials" had zero visual contrast and an auditory stimulus at 0°. The ratio of unisensory visual/unisensory auditory/multisensory coherent/multisensory conflict/neutral trials varied between sessions but was ~ 10/10/5/5/1, and stimulus side was selected randomly on each trial. When a mouse was trained with 5 auditory azimuth locations (Figures S1K–S1L), the additional azimuths were −30° and +30°. A central auditory cue was chosen, rather than an absence of auditory stimuli, to avoid the auditory stimulus acting as a "trial onset" cue. However, for experiments with bilateral inactivation (Figures S5Q–S5X), this central auditory stimulus was removed to ensure that the effects of inactivating posterior parietal cortex on visual trials could not be attributed to a change in perception of this auditory cue.

After stimulus onset there was a 500 ms open-loop period, during which the subject could turn the wheel without penalty, but stimuli were locked in place and rewards could not be earned. This period was included to disambiguate sensory responses from wheel movement—as stimulus and wheel movement are perfectly correlated during the closed loop period. The mice nevertheless typically responded during this open-loop period (Figure S1F). At the end of the open-loop period, an auditory Go cue was delivered through all speakers (10 kHz pure tone for 0.1 s) and a closed-loop period began in which the stimulus position (visual, auditory, or both) became coupled to movements of the wheel. Wheel turns in which the top surface of the wheel was moved to the subject's right led to rightward movements of stimuli on the speaker array and/or screen, that is, a stimulus on the subject's left moved toward the central screen. For visual or auditory stimuli, the position updated at the screen refresh rate (60Hz) or the rate of stimulus presentation (8Hz). In trials, where auditory stimuli were presented at 0°, the auditory stimulus did not move throughout the trial. A left or right turn was registered when the wheel was turned by an amount sufficient to move the stimulus by 60° in either azimuthal direction (~ 30° of wheel rotation, although this varied across mice/sessions); if this had not occurred within 1 s of the auditory Go cue, the trial was recorded as a "timeout." On unisensory visual, unisensory auditory, and multisensory coherent trials, the subject was rewarded for moving the stimulus to the center. If these trials ended with an incorrect choice, or a timeout, then the same stimulus conditions were repeated up to a maximum of 9 times. In neutral and conflicting multisensory trials, left and right turns were rewarded with 50% probability (Figure S1A), and trials were only repeated in the event of a timeout, not an unrewarded choice. An incorrect choice or timeout resulted in an extra 2 s delay before the next trial for all stimulus conditions. After a trial finished (i.e. after either reward delivery or the end of the 2 s delay), an inter-trial interval of 1.5–2.5 s (uniform distribution) occurred before the software began to wait for the next quiescent period. Behavioral sessions were terminated at experimenter discretion once the mouse stopped performing the task (typically 1 h).

Mice were trained in stages (Figure S1B). First, they were trained to ~ 70% performance with only coherent trials; then auditory, visual, and neutral/conflict trials were progressively introduced based on experimenter discretion. Using this training protocol, ~ 80% of mice learned the task, and those that did learn reached the final stage in <30 sessions (Figure S1C).

Optogenetic inactivation

For optogenetic inactivation experiments (Figures 2, S4, and S5) we inactivated several cortical sites through the skull using a blue laser,[59–62] in transgenic mice expressing ChR2 in Parvalbumin-expressing inhibitory interneurons (Ai32 x PV-Cre). Unilateral inactivation was achieved using a pair of mirrors mounted on galvo motors (GVSM002-EC/M, Thor labs) to orient the laser (L462P1400MM, Thor labs) to different points on the skull. On every trial, custom code drove the galvo motors to target one of 52 different coordinates distributed across the cortex (Figure 2A), along with 2 control targets outside of the brain (Figure S4C). A 3D-printed isolation cone prevented laser light from reaching the screens and influencing behavior. Inactivation coordinates were defined stereotaxically from bregma and were calibrated on each session. Anterior-posterior (AP) positions were distributed across 0, ± 1, ± 2, ± 3, and −4 mm. Medial-lateral (ML) positions were distributed across ± 0.6, ± 1.8, ± 3.0, and ± 4.2 mm. On 75% of randomly interleaved trials, the laser (40 Hz sine wave, 462 nm, 3 mW) illuminated a pseudorandom location from stimulus onset until the end of the response window 1.5 s later (both open and closed loop periods, irrespective of mouse reaction time). The laser was not used on trial repetitions due to incorrect choices or timeouts. Pseudorandom illumination meant that a single cortical site was inactivated on only 1.4% of trials per session. This discouraged adaptation effects but required combining data across sessions for analyses. The galvo-mirrors were repositioned on every trial, irrespective of whether the laser was used, so auditory noise from the galvos did not predict inactivation. For bilateral optogenetic inactivation (Figures S5Q–S5X), the same strategy was used, but the galvo motors flipped between two locations at 40 Hz, effectively providing 20 Hz stimulation at each location. The laser power was reduced to zero when the galvo motors moved between locations. This resulted in a reduced laser power of ~ 2 mW.

To investigate the effects of inactivation at different time points (Figures 2J and 2K) in separate experiments, the laser was switched on for 25 ms (DC) at random times relative to stimulus onset (−125 to +175 ms drawn from a uniform distribution). Inactivation was randomly targeted to visual areas (VISp; −4 mm AP, ±2 mm ML) or secondary motor area (MOs; +2 mm AP, ±0.5 mm ML) on 25% of trials.

Neuropixels recordings

Recordings in behaving mice were made using Neuropixels (Phase3A;[99]) electrode arrays, which have 384 selectable recording sites out of 960 sites on a 1 cm shank. Probes were mounted to a custom holder (3D-printed polylactic acid piece) affixed to a steel rod held by a micromanipulator (uMP-4, Sensapex Inc.). Probes had a soldered external reference connected to ground which was subsequently connected to an Ag/AgCl wire positioned on the skull. On the first day of recording mice were briefly anesthetized with isoflurane while one or two craniotomies were made with a biopsy punch. After at least 3 h of recovery, mice were head-fixed in the usual position. The craniotomies, as well as the ground wire, were covered with a saline bath. One or two probes were advanced through the dura, then lowered to their final position at approximately 10 μm/s.

Electrophysiological data were recorded with Open Ephys.[100] Raw data within the action potential band (1-pole high-pass filtered over 300 Hz) was denoised by common mode rejection (that is, subtracting the median across all channels), and spike-sorted using Kilosort[97] version 2.0 (www.github.com/MouseLand/Kilosort2). Units were manually curated using Phy to remove noise and multi-unit activity.[101] Each cluster of events (‘unit’) detected by a particular template was inspected, and if the spikes assigned to the unit resembled noise (zero or near-zero amplitude; non-physiological waveform shape or pattern of activity across channels), the unit was discarded. Units containing low-amplitude spikes, spikes with inconsistent waveform shapes, and/or refractory period contamination were labeled ‘multi-unit activity’ and not included for further analysis.

To localize probe tracks histologically, probes were repeatedly dipped into a centrifuge tube containing DiI before insertion (ThermoFisher Vybrant V22888 or V22885). When probes were inserted along the same trajectory for multiple sessions (Figure S6A), they were coated with DiI on the first day, and subsequent recordings were estimated to have the same trajectory within the brain (although depth was independently estimated, Figure S6B). After experiments were concluded, mice were perfused with 4% paraformaldehyde. The brain was extracted and fixed for 24 h at 4°C in paraformaldehyde before being transferred to 30% sucrose in PBS at 4°C. The brain was then mounted on a microtome in dry ice and sectioned at 80 μm slice thickness. Sections were washed in PBS, mounted on glass adhesion slides, and stained with DAPI (Vector Laboratories, H-1500). Images were taken at 4× magnification for each section using a Zeiss AxioScan, in two colors: blue for DAPI and red for DiI. Probe trajectories were reconstructed from slice images (Figure S6A) using publicly available custom code (http://github.com/petersaj/AP_histology[102]). For each penetration, the point along the probe where it entered the brain was manually estimated using changes in the local field potential (LFP) signal (Figure S6B). Recordings were made in both left (47 penetrations) and right (41 penetrations) hemispheres. The position of each recorded unit within the brain was estimated from its depth along the probe. For visualization, the recorded cells were mapped onto a flattened cortex using custom code (Figure 3A). Given the small size the frontal pole, neurons in this region could not be confidently separated from MOs, and so were considered part of MOs for the purpose of this manuscript (14% of MOs cells; excluding these cells did not significantly impact results).

For recordings from naive mice (Figures 5, 6C, 6D, and 6F), data were acquired with 4-shank Neuropixels 2.0 probes, which have 384 selectable recording sites out of 5,000 sites on 4 1 cm shanks.[103] We recorded from the 96 sites closest to the tip of each shank. Electrophysiological data for these experiments were recorded with SpikeGLX (https://billkarsh.github.io/SpikeGLX/). The same procedures were followed as above for mouse surgery and manual curation of units. Changes in LFP signal were used to detect the point at which the probe entered the brain, and only cells within 1.25mm of the brain surface (i.e. within MOs) were included in analyses.

Passive stimulus presentation recordings

Mice were presented with task stimuli under passive conditions after each behavioral recording session. Although the wheel remained in place, stimuli were presented in open-loop (entirely uncoupled from wheel movement) and mice did not receive rewards. Unisensory auditory, unisensory visual, coherent, and conflict trials were presented to mice. However, on unisensory visual trials, the auditory amplitude was set to zero (rather than positioned at 0° as in the task) to ensure visual sensory responses could be isolated. Due to time constraints, only one coherent and conflicting stimulus combination were presented (80% contrast in both cases), and the trial interval was reduced (randomly selected from 0.5 to 1 s). Stimulus conditions were randomly interleaved, and each condition was repeated ∼ 50 times.

QUANTIFICATION AND STATISTICAL ANALYSIS

Statistics

Statistical tests used in each analysis can be found in the corresponding figure legend, and in the STAR Methods. Where relevant, the definition of center, dispersion, and precision measures (e.g. MAD vs. SD) are described in the text or figure legend. Sample sizes were not estimated prior to data collection. Statistical tests were selected according to the typified distribution for each data type, but we did not perform additional analyses to test the statistical assumptions of each test. Blinding of the experimenter was not applicable to these analyses. Where data were excluded, the reasoning is described in the corresponding section. Statistical

tests used, the value of n, and what n represents in each analysis can be found in the corresponding figure legend or in the STAR Methods.

Behavioral quantification

With the exception of specific analyses of timeout trials (Figures 2L and S5I–S5P), timeouts and repeats following incorrect choices were excluded. To remove extended periods of mouse inattention at the start and end of experimental sessions, we excluded trials before/after the first/last three consecutive choices without a timeout. The 6% contrast level was included in analyses of inactivation experiments (Figure 2) as all mice contributing to these analyses were presented with 6% contrast levels, but not all behavioral and electrophysiology sessions included 6% contrast.

On 91.5% of trials (142853/156118), subjects responded to the stimulus onset by turning the wheel within the 500 ms open-loop period (Figure S1F). For data analysis purposes, we therefore calculated mouse choice and reaction time from any wheel movements after stimulus onset (Figures S1D and S1E), even though during the task, rewards would only be delivered after the open-loop period had ended. These choices were defined by the first time point at which the movement exceeded $\sim 30^\circ$ of wheel rotation (the exact number varied across sessions/mice, Figure S1D), the same threshold required for reward delivery during the closed-loop period. This matched the outcome calculated during the closed loop period on 94.9% of trials (148203/156118). The reaction time was defined as the last time prior to the choice threshold at which velocity crossed 0 after at least 50 ms at 0 or opposite to the choice direction, and then exceeded 20% of the choice threshold per second for at least 50 ms (Figure S1E). On 5.1% of trials (8380/164498), no such timepoint existed or movement was non-zero within 10 ms of stimulus onset; these trials were excluded. On 38% of trials (59498/156118), mice made sub-threshold movements prior to their calculated reaction time. To eliminate the possibility that these earlier movements were responsible for the neural decoding of choice (Figure 3G) we repeated this analysis using only trials without any movement prior to the calculated reaction time (Figure S6C), which did not change the results.

When calculating performance for each stimulus type for a single contrast (Figures 1E, S1N, and S1P), the value for each mouse was calculated within each session before taking the mean across sessions. We then took the mean across symmetric presentations of each stimulus condition (e.g. unisensory auditory left and right trials). In the case of reaction time (Figures S1H–S1J), we calculated the median for each session before taking the mean across sessions and symmetric presentations. For relative reaction time (Figures 1B, S1O, S1Q, and 6D) we also subtracted the mean across all stimulus types for each mouse. For both performance and reaction time, differences between stimulus types were quantified with a paired t test (n = 17 mice). Using this analysis, we established that reaction times were faster on unisensory auditory trials than unisensory visual trials (Figure 1B). To confirm that the earlier movements on unisensory auditory trials were genuine choices rather than reflexive movements unrelated to the stimulus location, we predicted whether stimuli were presented on the right or left in unisensory auditory and unisensory visual trials from the wheel velocity at each timepoint after stimulus onset. Trial data was subsampled for each session (to equalize the number of stimuli appearing on the left and right) and split into test and training data (2-fold cross validation). Mean prediction accuracy was calculated by first taking the mean across sessions, then across mice. Consistent with our conclusions from calculated reaction times, auditory location could be decoded earlier than visual location (Figure S1G). This conclusively demonstrates that mice were able to identify the location of an auditory stimulus earlier than a visual stimulus.

Video motion energy analysis

Because neural activity across the brain is related to bodily motion,[68,69] we asked if mice still respond to stimuli in the passive condition. We filmed the mouse at 30 frames per second (DMK 23U618, The Imaging Source). We quantified the motion energy on each trial by averaging the absolute temporal difference in the pixel intensity values, across all pixels in a region of interest including the face and paws, and across a time period 0 to 400 ms after stimulus onset, which typically included the mouse response during behavior (Figure S1F). This analysis established that mice exhibit minimal movement in response to task stimuli during passive conditions (Figure S7T).

Wheel movement during active vs. passive conditions

To ask whether mice might still move the wheel in response to stimuli during passive stimulus presentation (Figures S7U–S7V) we calculated the absolute difference in wheel position between stimulus onset time and 0.5 s post-stimulus onset for five mice, and then took the mean across mice. We compared this value to a shuffled distribution generated from the same trials, using the same method, but with the stimulus onset time randomized within each trial (this process was repeated 1000 times). The wheel position at 0.5 s after stimulus onset was considered significant if the unshuffled value was in the top 5% of the values in the shuffled distribution.

Psychometric modeling

The model we use throughout the text, the (parametric) additive model, is given by the equation

$$log\left(\frac{P(R)}{P(L)}\right) = b + \left(v_R V_R^{\ \gamma} - v_L V_L^{\ \gamma}\right) + \left(a_R A_R - a_L A_L\right)$$

Model parameters were fit by maximizing the likelihood of observed behavioral data using MATLAB's *fmincon* function to implement the *interior-point* algorithm to find 6 fit parameters: v_R , v_L a_R and a_L representing sensitivities to right and left visual and auditory stimuli, b representing bias, and the contrast gain parameter γ. When fitting for individual mice (Figures 1C, 1F, S1K, and S3A–S3O), models were fit to data combined across sessions. When the model was fit to combined data from multiple mice (Figures 1D, 1G, 2G–2H, S2, and S4E–S4H), trials were subsampled to equalize numbers across mice before fitting the model. This subsampling process was repeated 10 times, and plots reflect the mean model parameters, and fraction of rightward choices, across repeats. For visualization, if the log-odds were not defined for a given stimulus condition (because a mouse, or mice, made only rightward or leftward choices) the log odds were regularized by adding one trial in each direction. This was only necessary for the coherent stimulus condition at 10% contrast in Figure 2H.

We compared our additive model to a range of other models (Figure S2), all fit the same way. The "auditory-only" model (Figure S2A) was given by:

$$log\left(\frac{P(R)}{P(L)}\right) = b + (a_R A_R - a_L A_L)$$

And the "visual-only" model (Figure S2B) was given by:

$$log\left(\frac{P(R)}{P(L)}\right) = b + (v_R {V_R}^{\gamma} - v_L {V_L}^{\gamma})$$

For the "auditory dominance" model (Figure S2C), we set the visual weight to zero whenever auditory and visual stimuli were in conflict:

$$log\left(\frac{P(R)}{P(L)}\right) = b + (1 - T_{con})(v_R {V_R}^{\gamma} - v_L {V_L}^{\gamma}) + (1 - a_{con}T_{con})(a_R A_R - a_L A_L)$$

Here, T_{con} is a binary variable, equal to 1 or 0 to indicate whether each trial is a multisensory conflict trial, and a_{con} is an additional fit parameter. We tested this model both with $a_{con} = 0$ (Figure S2C) and with a_{con} allowed to take any value (Figure S2D).

As a more general test for any evidence of visual or auditory dominance during audiovisual trials, we fit a "sensory bias" model (Figure S2E) with additional auditory and visual weights on coherent and conflict trials:

$$log\left(\frac{P(R)}{P(L)}\right) = b + (1 - v_{con}T_{con})(1 - v_{coh}T_{coh})(v_R {V_R}^{\gamma} - v_L {V_L}^{\gamma}) + (1 - a_{con}T_{con})(1 - a_{coh}T_{coh})(a_R A_R - a_L A_L)$$

Here, $a_{con}, v_{con}, a_{coh}$, and v_{coh} are fit parameters and T_{coh} is a binary variable, equal to 1 or 0 to indicate whether each trial is a multisensory coherent trial. Our 6-parameter additive model is a special case of this 10-parameter sensory bias model, when the 4 multisensory parameters are zero.

The 11-parameter "additive unconstrained" model (Figure S2F) is similar to the usual additive model, but can fit any function of contrast, not just a power function:

$$log\left(\frac{P(R)}{P(L)}\right) = b + v_i V_i + a_j A_j$$

Here V_i and A_j are binary variables indicating the presence of contrast i and auditory location j on a given trial. The parameters v_i and a_j represent the visual and auditory sensitivities to contrast auditory location j, and are constrained to be 0 for zero contrast visual and central auditory stimuli.

Finally, to determine whether a generic non-additive model of multisensory integration could improve model fit, we tested a 27-parameter "full model" which had a weight for each combination of auditory and visual stimuli (Figures 1H and S3P).

$$log\left(\frac{P(R)}{P(L)}\right) = w_{ij} V_i A_j$$

We evaluated the fit of each model by its log_2-likelihood ratio relative to a bias-only model $log(p(R|A,V)/p(L|A,V)) = b$ using 5-fold cross-validation. After normalizing by the number of trials, this yields a quantity in bits per trial: the number of bits two parties would save in communicating the mouse's choice, if the stimulus is known to both. We compared all models to the additive parametric model (Figures 1H and S2). Across 17 mice, the additive model was not significantly worse than the full model, either when trained on all trial types (Figure 1H), or when trained only on unisensory and neutral trials but tested on all trials including multisensory combinations (Figure S3P), suggesting that mice use the same behavioral strategy on all multisensory trials.

When fitting the additive model to data where different regions of dorsal cortex were inactivated, three target locations were combined to represent each region. For visual cortex (Figures 2G, 2I, 2L, S4H, and S5A–S5P), these were (−4,1.8), (−4,3) and (−3,3), where coordinates indicate (AP, ML) distances from bregma in mm. For frontal cortex (2,0.6), (2,1.8) and (3,0.6) (Figures 2H, 2I, 2L, S4G–S4H, and S5A–S5P); for lateral areas proximal to auditory cortex (−4,4.2), (−2,4.2), (−2,4.2) (Figures S4E, S4H, and S5A–S5P); for areas proximal to somatosensory cortex (1,3), (0,3), (0,4.2) (Figures S4F and S4H). When fitting these models, the

contrast gain parameter was fixed at the value obtained when fitting to non-inactivation trials. During bilateral inactivation (Figures S5Q–S5X), coordinates were as follows: for frontal cortex (2, ± 0.5), (2, ± 1.5) and (3, ± 0.5); for visual cortex (−4, ± 1.5), (−4, ± 2.5) and (−3, ± 2.5); for parietal cortex (−2, ± 1.5), (−2, ± 2.5), and (−2, ± 3.5).

For a mouse presented with 5 auditory conditions (Figures S1K–S1L), the additive model contained two additional auditory parameters, such that each non-zero auditory azimuth had a distinct weight:

$$log\left(\frac{P(R)}{P(L)}\right) = b + \left(v_R V_R{}^{\gamma} - v_L V_L{}^{\gamma}\right) + \left(a_{R_{60}} A_{R_{60}} + a_{R_{30}} A_{R_{30}} - a_{L_{60}} A_{L_{60}} - a_{L_{30}} A_{L_{30}}\right)$$

Here, R_{60}, R_{30}, L_{60}, and L_{30} indicate whether the auditory stimulus was presented at 30° or 60° on the left or right.

Quantifying effects of optogenetic inactivation on choice

To quantify the change in the fraction of rightward choices when a particular cortical location was inactivated, we used a shuffle test (Figures 2B–2E, S4A, and S4B). Data were initially combined across 5 mice and segregated by stimulus type (unisensory visual, unisensory auditory, multisensory coherent, or multisensory conflict). For each type, data were further segregated into non-inactivation trials (laser off) and inactivation trials (laser on) grouped by the targeted area of dorsal cortex. For trials where the stimulus was presented on the right, we reversed the laterality of the stimulus and inactivation location such that all stimuli were effectively presented on the left (visual stimulus in the case of conflict trials). Data were randomly subsampled (from a total of ~ 80,000 trials) to equalize the number of trials contributed by each mouse to non-inactivation and inactivation trials at each targeted location. We then calculated the difference in the fraction of rightward choices for each targeted location compared with non-inactivation trials. This process was repeated 25,000 times with different subsampling to produce a mean change in fraction of rightward choices for each inactivated location on dorsal cortex.

For each of the 25,000 iterations, we proceeded to generate 10 independent shuffles, where the labels for targeted location and trial identity (inactivation or non-inactivation) where randomly reassigned. We thus generated a null distribution for each targeted location, comprising 250,000 datapoints from independent shuffles. For each targeted location, the position of the unshuffled result within this null distribution gave the significance value for that location (e.g. top/bottom 0.05% for $p < 0.001$, top/bottom 0.005% for $p < 0.0001$).

When assessing the symmetry of inactivation effects across hemispheres (Figure S4A) the process was as described above, but without reversing the laterality of any trials. To confirm results were similar across mice (Figure S4B), we repeated this process for individual mice. In this case, the number of shuffled iterations remained at 250,000 but no subsampling was required (because there was no need to equalize across mice).

To test how pulsed inactivation at different times affected choices (Figures 2J and 2K), data were combined across 7 mice. Trials where stimuli appeared on the right were reversed such that an increase in the fraction of rightward choices corresponded to an increase in the fraction of ipsilateral choices. Experimental sessions with fewer than 75 inactivation trials were excluded to ensure that each session contributed to both the fraction of inactivation and control trials. Laser onsets were binned using a sliding 70 ms boxcar window, and the time between stimulus onset and inactivation was defined as the center of this window. In each 70 ms time window, we calculated the change in fraction of rightward choices compared with non-inactivation trials, and the significance of this difference was established with a Fisher's exact test. Each timepoint was defined as significant if it, or both its neighboring timepoints, passed the significance criterion of $p < 0.001$.

Quantifying effects of optogenetic inactivation on model parameters

To quantify the changes in parameters of the additive model (Figures 2F and S4D) the analysis closely mirrored the steps described above, but trial types were not segregated by stimulus type. This increased statistical power compared with analyses of separate stimulus types (above), allowing for the detection of more subtle changes in mouse behavior. The additive model was reparametrized such that stimuli were defined as being ipsilateral or contralateral to the site of inactivation, effectively combining data across hemispheres:

$$log\left(\frac{P(I)}{P(C)}\right) = b + \left(v_i V_i{}^{\gamma} - v_c V_c{}^{\gamma}\right) + \left(a_i A_i - a_c A_c\right)$$

Here, V_c and V_i are contralateral and ipsilateral contrasts, and A_c and A_i are contralateral and ipsilateral auditory azimuths. v_i , v_c a_i and a_c represent sensitivities to contralateral and ipsilateral visual and auditory stimuli, while b represents the bias, and γ the contrast gain parameter. The unshuffled dataset comprised 2,500 different subsamples, and in each iteration, we fit the additive model to the non-inactivation data and to the inactivation data for each targeted location. This gave the mean change in each model parameter at each location on dorsal cortex. We compared this value to a null distribution (generated as described above, total of 25,000 independent shuffles) to establish the significance of each change. Since we observed no change in the contrast gain parameter, γ (Figure S4D), in our final analysis we fixed this value according to the non-inactivation trials and only quantified changes in the remaining 5 parameters (Figure 2F).

To determine whether inactivating these regions caused a significant change in model parameters compared with non-inactivation trials, we evaluated the log likelihood ratio between a model trained and evaluated on inactivation trials and a model trained on non-

CellPress
OPEN ACCESS

inactivation trials and then evaluated to inactivation trials for individual mice. We then determined if the log likelihood ratio was significantly different from zero across the 5 mice using a t test (Figures 2G, 2H, S4E, and S4F).

To test whether inactivation of the four different regions (frontal, visual, lateral sensory, and somatosensory cortices) had significantly different effects we used a shuffle test to evaluate data combined across all mice (Figure S4H). For each pair of regions, as well as non-inactivation trials, we calculated inter-region log likelihood (where the model was fit to trials from one region and then evaluated on another region) and a within-region log likelihood (where the model was trained and evaluated on data from one inactivated region). We repeated this process in 100 different subsamples, equalizing the number of trials from each mouse, and the number of trials in the train and test sets, and took the mean log likelihood ratio between the inter-region and within-region results. We then generated a null distribution by repeating this process, but with the label of the inactivation site shuffled before splitting the data to perform the inter-region and within-region comparison (total of 1,000 independent shuffles). For each pairwise regional comparison, we compared the mean unshuffled log likelihood ratio to the null distribution and found that every inter-region log likelihood was significantly lower than the within-region log likelihood ($p < 0.05$, Bonferroni-corrected).

The effect of inactivation on reaction time, fraction of timeout/slow trials, and rightward choices

We used a linear mixed effects model (LME) to determine the effect of inactivating visual, lateral, or frontal cortices on mouse reaction time for each stimulus type (auditory, visual, coherent and conflicting) when stimuli were contralateral or ipsilateral to the site of inactivation (Figures 2I and S5A–S5H). For each mouse, we computed the median reaction time over trials of all sessions for each combination of stimulus condition and inactivation region. We fit the following LME model to this data using MATLAB's *fitlme* function:

$$\textit{Reaction time} \sim \textit{Inactivation} + \textit{VisualContrast} + (1|\textit{MouseID})$$

Here, *Reaction time* is the response variable, *Inactivation* (binary) and *VisualContrast* (categorical) were fixed effect terms, and *MouseID* was a random effect on the intercept. We separate LMEs for each stimulus type and region of inactivation. In each case, we assessed the sign and significance of the *Inactivation* term to assess the impact of inactivation on mouse reaction time (Figures 2I and S5A–S5H). To make direct inter-region comparisons, we modified the LME model:

$$\Delta \textit{Reaction time} \sim \textit{InactivationRegion} + \textit{VisualContrast} + (1|\textit{MouseID})$$

Here, Δ*Reaction time* is the difference in reaction time between the inactivated trials and non-inactivation trials for each stimulus condition (for each stimulus condition within a stimulus type). *InactivationRegion* is a binary fixed effect term identifying which brain region (of the two being compared) was inactivated (for example, frontal and visual, Figure 2I). As above, we assessed the sign and significance of the *InactivationRegion* term to determine whether the inactivation region had a significant effect on the change in reaction time (Figures 2I and S5A–S5H).

Statistical analyses of timeout trials were performed in the same way as the two previous LMEs, but *Reaction time* was replaced with *Fraction of timeouts* (the fraction of responses greater than 1.5 s) and Δ*Reaction time* was replaced with Δ*Fraction of timeouts* (Figures 2L and S5I–S5P).

For statistical analyses of the effect of bilateral inactivation on reaction time (Figures S5Q–S5X), slow trials were defined as all trials with reaction times greater than 300 ms. We used this binarization, rather than raw reaction time, because we did not have enough bilateral inactivation trials to accurately estimate the reaction time. Analyses were performed as described above, but *Reaction time* was replaced with *Fraction of slow trials* and Δ*Reaction time* was replaced with Δ*Fraction of slow trials* (Figures S5Q–S5T).

For statistical analyses of the effect of bilateral inactivation on the fraction of rightward choices, trials with stimuli on the left and right were combined after reversing the choice direction for trials with stimuli on the right (the visual stimulus in the case of conflict trials). Analyses were performed as described above, but *Reaction time* was replaced with *Fraction of rightward choices* and Δ*Reaction time* was replaced with Δ*Fraction of rightward choices* (Figures S5U–S5X).

Estimating firing rate

Unless otherwise specified, firing rates were calculated on each trial by binning in 2 ms windows and smoothing with a half-Gaussian filter with standard deviation of 60 ms. PSTHs were calculated by averaging this rate across trials.

Decoding stimuli and choices from population activity

To decode stimuli and choices from neural activity (Figures 3E–3G, S6C, and S6D), we trained a linear support vector machine (SVM) decoder on the firing rate vector time-averaged over a window 0–300 ms after stimulus onset (Figures 3E and 3F), 0–130 ms before movement onset (Figures 3G and S6C), or 150–300 ms after movement onset (Figure S6D). SVMs were trained separately for each Neuropixels behavioral recording. To ensure that differences in decoding accuracy between brain areas and between experiment sessions could not be attributed to differences in the number of neurons recorded, we repeatedly (5 repeats) selected a 30-neuron subset for decoding analysis and took the mean accuracy (5-fold cross-validated) across these repeats. Sessions with fewer than 25 trials of each decoded condition (e.g. left and right stimulus locations), and brain regions with less than 30 neurons recorded in that session, were excluded. In the case of decoding visual location (Figure 3E), only trials with high-contrast (40% and 80%) stimuli were included. In each session, decoding accuracy was quantified as the fraction of test-set trials classified

correctly, relative to the same number for a model with no access to the spike trains (whose optimal behavior is to always predict the most common stimulus on the training set):

$$Accuracy = \frac{Neural\ decoding\ accuracy - Baseline\ accuracy}{1 - Baseline\ accuracy}$$

To compare the decoding accuracy between brain regions, we first performed a one-way ANOVA, which showed a significance difference (visual location: F = 26.1, $p < 10^{-20}$, auditory location: F = 77.7, $p < 10^{-67}$, and upcoming choice: F = 21.0, $p < 10^{-13}$). To compare pairwise differences, we fit a linear mixed effects model:

$$Accuracy \sim Brain\ region + (1|MouseID)$$

Here, *Accuracy* is defined as above, *Brain region* is a categorical fixed effect and *MouseID* was a random effect on the intercept, to take account of the potential confound of differences in decoding accuracies across mice (Figures 3E–3G).

We used the same definition of decoding accuracy 0–130 ms before movement onset to investigate the relationship between choice decoding and behavioral performance—defined as the percentage of correct choices—in individual experimental sessions (Figure S6F, right panel). We then used a linear mixed effects model to test for a significant effect of behavioral performance on decoding accuracy whilst controlling for differences across mice:

$$Accuracy \sim performance + (1 + performance|Mouse\ ID)$$

Where *peformance* is a continuous fixed effect and we allow for a random effect on both the slope and the intercept. Using the same form of linear mixed effects model, we tested for a continuous fixed effect of the mean anterior-posterior (Figure S6F, left panel) and medial-lateral (Figure S6F, middle panel) recording location. Mean location was calculated from the subsampled neurons used for decoding on each probe in each session.

Quantifying ramping of choice-related activity in MOs

To quantify the population dynamics of the choice-related activity in MOs (Figure S6E), we first computed the mean population vector corresponding to leftward choices $\overrightarrow{\mu_L}$ and rightward choices $\overrightarrow{\mu_R}$ by taking the mean activity across trial and from 0 to 100 ms after movement onset. Then, for each time bin of neural activity during each trial, $\overrightarrow{x(t)}$, we obtained the cosine similarity of the population vector with the difference between the rightwards and leftward choice population vectors:

$$S_c\left(\overrightarrow{x(t)}, \overrightarrow{\mu_R} - \overrightarrow{\mu_L}\right) = \frac{\overrightarrow{x(t)} \cdot (\overrightarrow{\mu_R} - \overrightarrow{\mu_L})}{\|\overrightarrow{x(t)}\| \, \|\overrightarrow{\mu_R} - \overrightarrow{\mu_L}\|}$$

To cross validate the results for each stimulus condition, we held out the within-condition data and computed the choice vectors from all other stimulus conditions. Before computing these vectors, we balanced the number of trials with leftward and rightward choices to prevent stimulus-related activity from biasing the projection onto the choice axis.

Combined-conditions choice/stimulus probability analysis

To quantify the selectivity of a cell for a choice while controlling for effects of stimulus (Figure S6J), we used the combined-conditions choice probability (ccCP,[95]). This is based on an extension of the Mann-Whitney U statistic, defined as the fraction of pairs of trials of identical stimulus conditions but different choices, for which the firing rate on the right choice trial exceeds the firing rate on the left choice trial. The significance of this test statistic was evaluated by shuffling using a p value of 0.01, meaning that the observed value has to be either below the 0.5 percentile or above the 99.5 percentile of a null distribution generated from 1000 shuffles of the choice labels for each stimulus condition in order to be deemed significant. For ccCP, we compared the firing rate averaged over 0–130 ms before movement onset between trials where the mouse made a leftward or rightward choice trials (Figure S6J).

To test for selectivity to one stimulus while controlling for the other stimulus and choice (Figure S6I), we used an analogous method, referred to as the combined conditions stimulus probability (ccSP). For visual ccSP, we compared the firing rate time-averaged over a 0–300 ms window after stimulus onset, between trials where the visual stimulus was on the left and trials where the visual stimulus was on the right, including only trials with high (40% or 80%) contrast (Figure S6I, left). For auditory ccSP, we compared the firing rate averaged over a time window 0–300 ms after stimulus onset between auditory-left and auditory-right trials (Figure S6I, right).

Modeling neural activity

To predict firing rate time courses from task events (Figures 4A–4C), we used an ANOVA-style decomposition. For this analysis, we pooled multisensory coherent and conflict trials of contrast 40% and 80% (using a single visual contrast did not impact results), resulting in four possible stimulus conditions: one for each combination of auditory and visual location. We defined binary variables $a_i, v_i, c_i = \pm 1$ encoding whether auditory stimuli, visual stimuli, and choices are to the left or right on trial i. We can decompose $\boldsymbol{F}_i(t)$, the firing rate vector on trial i at time t after stimulus onset, as:

$$\boldsymbol{F}_i(t) = \boldsymbol{B}(t) + a_i\boldsymbol{A}(t) + v_i\boldsymbol{V}(t) + a_iv_i\boldsymbol{N}(t) + \boldsymbol{M}(t - \tau_i) + c_i\boldsymbol{D}(t - \tau_i)$$

CellPress
OPEN ACCESS

This model decomposes the response into a sum of 6 temporal kernels. ***B*** represents the grand mean stimulus response; ***A*** and ***V*** represent the additive main effects of auditory and visual stimulus location, and ***N*** represents a non-additive interaction between them. To account for the effects of movement, ***M*** is a kernel representing the mean effect of movement (relative to τ_i, the time of movement onset on trial *i*) and ***D*** represents the effect of movement direction. $\boldsymbol{B}, \boldsymbol{A}, \boldsymbol{V}, \boldsymbol{N}$ were allowed to be non-zero for $-50 \leq t \leq 400$ ms. $\boldsymbol{M}, \boldsymbol{D}$ can be non-zero for $-200 \leq t - \tau_i \leq 700$ ms. Only trials with $\tau_i < 300$ ms were included. The model was fit using ridge regression with a regularization strength of $\alpha = 10$, which we found to give optimal prediction accuracy. We fit this model to each neuron in MOs with a non-zero firing rate during behavior (n = 2183 neurons), using a training set consisting of half the trials (randomly selected). The error, *E*, of this fit was measured as:

$$E = \frac{1}{N_i}\frac{1}{N_t}\sum_i\sum_t (y_{it} - \hat{y}_{it})^2$$

Here, $\hat{y}_{it}$ and y_{it} are model prediction and test-set recorded firing rate on trial *i* and timepoint *t*, N_i is the number of neurons, and N_t is the number of time bins, spanning 0 to 400 ms relative to stimulus onset. *E* is thus the cross-validated mean-squared error between the predicted and the actual smoothed firing rate over this time window. To test for an additive code, we then repeated this process for an additive neural model where $\boldsymbol{N} = 0$ (Figure 4C).

To investigate whether there was an interaction between stimulus condition and choice-related response, we also fit a model with 8 movement-aligned kernels, i.e. a movement and a direction kernel for each combination of the four possible audiovisual stimuli:

$$\boldsymbol{F}_i(t) = \boldsymbol{B}(t) + a_i\boldsymbol{A}(t) + v_i\boldsymbol{V}(t) + a_iv_i\boldsymbol{N}(t) + \boldsymbol{M}_{a_i,v_i}(t - \tau_i) + c_i\boldsymbol{D}_{a_i,v_i}(t - \tau_i)$$

We compared this full model to the additive neural model (two movement kernels and $\boldsymbol{N} = 0$) using the method described above (Figure S7A).

To model neural activity during passive stimulus presentation (Figures 4D–4G), we used a reduced model without movement-aligned kernels:

$$\boldsymbol{F}_i(t) = \boldsymbol{B}(t) + a_i\boldsymbol{A}(t) + v_i\boldsymbol{V}(t) + a_iv_i\boldsymbol{N}(t)$$

Here, only multisensory coherent and conflict trials of a single (80%) contrast were included (due to time constraints, this was the only contrast presented on multisensory trials in passive conditions). To test for an additive code, we repeated the process described above (on 2,509 cells with non-zero firing rates, Figure 4F). No regularization was used for this analysis of passive data as it did not improve fits.

To compare the fit of linear and non-linear models of neural firing (Figures 4C, 4F, and S7A), we used a linear mixed effects method to determine the main effects of the prediction model, accounting for systematic differences in model fit across mice and across experiments within each mouse. This was done using the *fitlme* in MATLAB with the following formula:

$$error \sim 1 + model + (1 + model|subject) + (1 + model|subject : session) + (1|subject : session : neruon)$$

The error term *E* is modeled with an intercept, a fixed effect of the model type being used (e.g. either the additive or full model), random effects for the intercept and model type grouped by subjects, random effects for the intercept and model type grouped by session nested within subjects, and random effects for the intercept grouped by neurons nested within sessions within subjects. For all statistical tests we report the p value of the main effect of the model type on the observed error values.

To examine the distribution of auditory and visual spatial sensitivity across neurons we used neural recordings from passive stimulus presentation (Figures 4G and S7B). We selected neurons where the additive neural model ($\boldsymbol{N} = 0$) explained a minimum of 2% variance. For each neuron, we averaged the amplitude of the ***A*** and ***V*** kernels over a time window from 0 to 300 ms after stimulus onset (the kernels were fit using all trials). To test for a significant correlation between the signed magnitude of these time-averaged ***A*** and ***V*** kernels, we used the linear mixed effects model described above, but with *time-averaged **V** kernel* and *time-averaged **A** kernel* substituted for *error* and *model* (Figure 4G). To test for a relationship between the absolute values of the two kernels, we repeated this procedure but using the absolute, rather than the signed, time-averaged kernels (Figure S7B).

Lateralization of stimulus and movement activity

To investigate whether there is lateralization in the spatial preference of auditory neurons, we examined time-averaged value of the ***A*** kernels (0 ms–300 ms after stimulus onset) after fitting the additive model ($\boldsymbol{N} = 0$) under passive conditions. We selected neurons for which the additive model performed better than a model with visual kernel alone, and compared the mean value of the ***A*** kernel for neurons recorded in each hemisphere (Figure S7D). We repeat the same procedure for the visual kernel weights to examine lateralization of visual spatial preference (Figure S7C).

To investigate the lateralization of movement-related responses, we repeated this procedure, but for the additive model ($\boldsymbol{N} = 0$) during behavior. We then included all for which the directional movement kernel, ***D*** improved cross-validated fits. Mean kernel values of selected neurons were calculated using a time window −200 to 400 ms relative to movement onset (Figure S7E).

Statistical analysis to determine the lateralization of sensory and movement responses were performed with linear mixed effects model as described above, but with *time-averaged kernel* and *hemisphere* substituted for *error* and *model*.

Quantifying stimulus and movement activity as a function of location within MOs

To investigate whether there was an over-representation of stimulus or choice related activity in a specific subregion of MOs, we selected probes with at least 30 recorded neurons in MOs. For each probe, we then computed the mean across neurons for the absolute time-averaged value of the stimulus and movement kernels (described above), medial-lateral position, and anterior-posterior position. We then calculated the Pearson's correlation between each position and the kernel values across all probes (Figures S7F, S7H, S7J, S7L, S7N, and S7P). When considering depth relative to the brain surface, we computed the mean absolute kernel sizes for each 0.09 mm bin of depth values (Figures S7G, S7I, S7K, S7M, S7O, and S7Q). We additionally controlled for higher firing rates in deeper regions of cortex, by dividing the firing rate of each neuron by the baseline firing rate 0–700 ms before stimulus onset.

Quantifying single-neuron discrimination time

To identify when visual and auditory information began to be encoded in MOs (Figure 4H), we analyzed responses to passive unisensory stimuli. We first used shuffle tests to select neurons sensitive to the presence (On-Off) and/or the location (Right-Left) of auditory and visual stimuli. To identify On-Off neurons, we calculated two PSTHs, one for sounds in each location, in a window 0 to 300 ms after stimulus onset, and computed the difference between the maximum of this PSTH and the mean firing rate 300 to 0 ms before stimulus onset. We compared this value to a null distribution obtained from 1000 shuffles of the pre/post-stimulus windows independently for each trial. A neuron was defined as significantly responding to a stimulus if the maximum difference in unshuffled data was in the first or 99th percentile of the null distribution for either left or right stimuli. For Right-Left neurons, the same method was used, but using the maximum difference between the PSTHs for left and right auditory or visual presentations 0 to 300 ms after stimulus onset, and shuffling the left/right trial labels. This method identified 72 auditory (3%) and 68 visual (3%) Right-Left neurons.

For identified On-Off neurons, we calculated the discrimination time by separately comparing the pre- and post-stimulus firing rate in a sliding window of 50 ms with step size 5 ms, defining significance using a Mann-Whitney U test at $p < 0.01$, and requiring three consecutive significant time windows to qualify as the discrimination time. We excluded discrimination times that occurred more than 300 ms after stimulus onset as they are unlikely to be stimulus-related activity. This analysis was done separately for left and right stimuli, taking the earliest statistically significant time window in either stimulus condition. For identified spatially selective (Right-Left) neurons, we defined the discrimination time as the earliest time after stimulus onset where there is a significant difference in the response to left and right stimuli (Figure 4H). This method identified discrimination times for 82 and 36 auditory and visual On-Off neurons, and 59 and 36 auditory and visual Right-Left neurons. For each neuron we also calculated the 5-fold cross-validated decoding accuracy, relative to a baseline model (which always predicts the most-frequent stimulus-condition in the training set, as in Figures 3E and 3F), from the time-averaged firing rate in a window 0 to 100ms after the discrimination time using a linear SVM decoder (Figure S7W).

Quantifying single-neuron discriminability index

To quantify single-neuron selectivity for sensory location and upcoming choice, we calculated the discriminability index (d' or d-prime) between different trial conditions (Figures 5, S6G, and S6H). The discriminability index is defined as:

$$d' = \frac{\mu_1 - \mu_2}{\frac{1}{2}(\sigma_1 + \sigma_2)}$$

Here μ_1 and μ_2 are the mean firing rate of the neuron 0–300 ms after stimulus onset for quantifying stimulus responses, or 0–130 ms before movement onset for quantifying choice coding, and σ_1 and σ_2 are the standard deviation of the firing rate across the respective trial conditions.

To compare single neuron discriminability indices across brain regions, we first performed a one-way ANOVA on the mean of the absolute value of the discriminability index across neurons of each recorded session for each brain region, which showed a significant difference between brain regions (visual location: $F = 5.57$, $p < 10^{-3}$, auditory location: $F = 5.67$, $p < 10^{-3}$, and upcoming choice: $F = 11.6$, $p < 10^{-9}$). To compare differences between individual brain regions, we fit a linear mixed effects model:

$$|d'| \sim \textit{Brain region} + (1|\textit{MouseID})$$

Here, $|d'|$ is the absolute mean discrimination index across neurons, *Brain region* is a categorical fixed effect and *MouseID* is a random effect on the intercept (Figures S6G–S6H). To compare single neuron discriminability indices between naive, trained mice and values obtained by shuffling the condition labels, we performed Welch's t test on the mean absolute discriminability index for each experimental session (Figure 5).

Accumulator model

To investigate whether the structure of the sensory code in MOs can explain mouse behavior, we fed this code into an accumulator model (Figure 6, similar to a drift diffusion model[71]). Since stimulus responses were sparse in MOs (140 auditory or visual location-selective neurons total from all experiments, as defined by the criteria of the previous section, i.e. 6%), we combined neural activity across all mice and experiments. To do so, we first obtained the PSTH for each stimulus condition, from −100 ms to 300 ms relative to stimulus onset. We then simulated 360 "trials" per stimulus condition by generating surrogate spike trains from a Poisson process

with intensity given by these PSTHs. The stimulus conditions include unisensory visual trials with contrasts of 10, 20, 40, and 80%, unisensory auditory trials, and coherent and conflict audiovisual trials where the contrast is at 80%. This process yielded a time-dependent rate vector $\boldsymbol{x}(t)$ for each trial, where t is time relative to stimulus onset.

The output of the accumulator model was a decision variable $d(t)$, produced by linearly accumulating neural activity:

$$d(t) = d(t-1) + \boldsymbol{x}(t) \cdot \boldsymbol{w}$$

Here, $\boldsymbol{w}$ is a set of time-independent weights that were learned by the model to optimize the speed and accuracy of its responses but were not fit to mouse behavior. The choice of the model is defined by the sign of the decision variable when it crosses one of the thresholds: +1 or −1 for a rightward or leftward choice, and the reaction time of the model is the time of this threshold-crossing relative to stimulus onset (Figure 6D).

To learn the weight vector $\boldsymbol{w}$, we define a target decision variable y for each trial, set to 1 or −1 for rightward or leftward stimuli on unisensory and coherent trials. On conflict trials, where there is no correct response, the target decision variable is randomly set to 1 or −1 with equal probability.

The weights were learned by minimizing a loss function that compares the target decision variable with the model's output decision variable for each trial:

$$\mathcal{L}(d(t)) = \sum_{t<0} d(t)^2 + \sum_{t \geq 0} \max(0, 1 - y\, d(t))$$

We used a mean-squared error loss before the stimulus onset ($t < 0$), to ensure that the model does not make a decision before the stimulus onset. After the stimulus onset ($t \geq 0$) we use a hinge-loss error, which is zero when the decision variable is above the threshold for the correct choice and penalizes incorrect decisions and decision values below the decision threshold. The loss function was minimized with respect to the weights of the model via gradient descent using the ADAM optimizer[104] with a learning rate of 0.01, and the gradient was obtained via automatic differentiation using the JAX library.[105] The model was trained on 70% of the trials for 300 epochs, and its behavior was evaluated on the remaining 30% of the trials (Figures 6E–6H). To simulate the inactivation of the visual cortex in the right hemisphere, we took the same learned model, but instead provided input where the activity of neurons that were previously identified as visual-left preferring neurons were decreased by 60% (Figure 6G). During training, the decision boundaries were set to +1 and −1. To account for the choice bias that was observed in mice, we performed grid search on the decision boundary values after model training in order to minimize the mean-squared error between the choice probability observed in the mice and in the model averaged across all stimuli conditions (Figure 6E). Decision boundaries were only fit on trials without simulated inactivation.

To simulate the inactivation of right MOs, we reduced the activity of right-hemisphere neurons by 60%. This manipulation did not recapitulate the lateralized effect of MOs inactivation (Figure S7X), because MOs neurons preferring either direction of stimulus are found equally in both hemispheres. To ask whether intra-hemispheric connections onto a downstream lateralized decision circuit could explain the lateralized effects of MOs inactivation, we trained another accumulator model with weights from neurons in the left and right hemisphere constrained to be positive and negative: those in the left hemisphere were constrained to have only zero or positive weights, and those in the right hemisphere were constrained to have only zero or negative weights. Because spatial neurons were bilaterally distributed, this constraint simulates a selective subsampling or connectional bias by downstream neurons, such that in each hemisphere, only the activity that contributes to a contralateral decision is being utilized. This model was able to predict the lateralized effect of MOs inactivation (Figure 6H). To test whether this weight-constrained model recapitulated the lateralized effect of MOs inactivation better than the original accumulator model, we repeated the sampling and fitting procedure (as described earlier) 100 times for each model and performed a two-sample unpaired t test on the mean-squared error between the model's prediction of the log-odds and the observed log-odds from mouse behavior ($p < 0.01$).

To test whether sensory code in the MOs of naive mice can produce the same behavior through the accumulator model (Figures 6C, 6D, and 6F), we first subsampled the neurons recorded in the MOs of naive mice so that the total number of neurons matched the number recorded in trained mice. To select the auditory and visual spatial neurons to be used in the accumulator model, we used the same shuffling procedure as above (see STAR Methods section on quantifying single neuron discrimination time). However, we adjusted the threshold which defines statistical significance so that the number of neurons selected from naive mice to feed into the accumulator model matched that used for trained mice. Once neurons were selected, we fit the accumulator model with the same procedure used for trained mice (Figure 6E). This subsampling procedure was repeated 5 times, and the mean result across repeated subsamples was used for visualization (Figures 6C, 6D, and 6F). To test whether the accumulator model fits (Figures 6E–6H) were better than expected by chance, we compared the mean-squared error between the model's prediction of the log odds, $\log(p(R)/p(L))$, with a null distribution obtained by fitting the same model after shuffling the stimulus conditions of each trial 100 times.

Optimally combining independent visual and auditory signals

Here we show that optimally combining information from two inputs with independent noise requires the log odds be an additive function of the two inputs. This is a classical result of probability theory, whose significance to neuroscience has been discussed in several prior works (e.g.[1,2]). We provide a proof for the specific case of a binary left/right choice based on auditory and visual information.

Let $S \in \{L, R\}$ represent the (left/right) location of the stimulus. A prior estimate for this location is captured by a prior probability distribution $p(S)$. The two sensory inputs A and V follow conditional probability distributions $p(V|S)$ and $p(A|S)$. We assume them to be conditionally independent:

$$p(V,A|S) = p(V|S)p(A|S).$$

By Bayes' theorem, the probability of the stimulus location given the inputs is:

$$p(S|V,A) = \frac{p(V,A|S)p(S)}{p(V,A)} = \frac{p(V|S)p(A|S)p(S)}{p(V,A)}$$

Write $p(R) = p(S = R)$ and $p(L) = p(S = L)$. Then, the log odds is given by:

$$\log\left(\frac{p(R|V,A)}{p(L|V,A)}\right) = \log\left(\frac{p(V|R)p(A|R)p(R)/p(V,A)}{p(V|L)p(A|L)p(L)/p(V,A)}\right)$$

$$= \log\left(\frac{p(V|R)}{p(V|L)}\frac{p(A|R)}{p(A|L)}\frac{p(R)}{p(L)}\right)$$

$$= \log\left(\frac{p(V\mid R)}{p(V\mid L)}\right) + \log\left(\frac{p(A\mid R)}{p(A\mid L)}\right) + \log\left(\frac{p(R)}{p(L)}\right)$$

The first term depends only on V, the second only on A, while the third is a constant. Thus, the log odds is an additive function:

$$\log\left(\frac{p(R\mid V,A)}{p(L\mid V,A)}\right) = f(V) + g(A) + b$$

For a binary choice $p(L \mid V,A) = 1 - p(R \mid V,A)$, so we can use the fact that the inverse function of $y = \log\left(\frac{x}{1-x}\right)$ is the logistic function $x = \sigma(y) = \frac{1}{1+e^{-y}}$, to obtain the formula

$$p(R\mid V,A) = \sigma(f(V) + g(A) + b)$$

Thus, the assumption of independent noise in two sensory modalities implies that an optimal estimate of stimulus location uses the logistic function applied to an additive combination of the two modalities. While some psychophysical models instead use cumulative Gaussian models, the cumulative Gaussian function does not arise naturally in the same way.

In our task, the assumption that $p(V|S)$ and $p(A|S)$ are conditionally independent holds only approximately. The fact that mice combine evidence additively thus indicates they are following a heuristic strategy.[14] To demonstrate this, we compare the actual values of $p(V,A|S)$ with those expected under the assumption of independence (Figure S8). To compute $p(V,A|S)$ we use Bayes' theorem: $p(V,A|S) = p(S|V,A)p(V,A)/p(S)$. We define the stimulus location S to be the direction of wheel turn that will lead to reward on a particular trial. Thus, for unisensory or coherent multisensory stimuli on the right $p(R|V,A) = 1$, for conflict stimuli $p(R|V,A) = 0.5$, and for unisensory or coherent multisensory stimuli on the left, $p(R|V,A) = 0$. The prior probability $p(R) = 0.5$. Thus $p(V,A|R)$ is the fraction of trials where (V,A) was presented if V and A are in conflict; twice this if V and A are unisensory or coherent right; and 0 if V and A are unisensory or coherent left. These probabilities (Figure S8A) are distinct from a conditional independence model $p(V|R)p(A|R)$ obtained by multiplying the marginals of this distribution (Figure S8B).

Neuron, Volume *111*

Supplemental information

Mouse frontal cortex mediates additive multisensory decisions

Philip Coen, Timothy P.H. Sit, Miles J. Wells, Matteo Carandini, and Kenneth D. Harris

Neuron, Volume *111*

Supplemental information

Mouse frontal cortex mediates additive multisensory decisions

Philip Coen, Timothy P.H. Sit, Miles J. Wells, Matteo Carandini, and Kenneth D. Harris

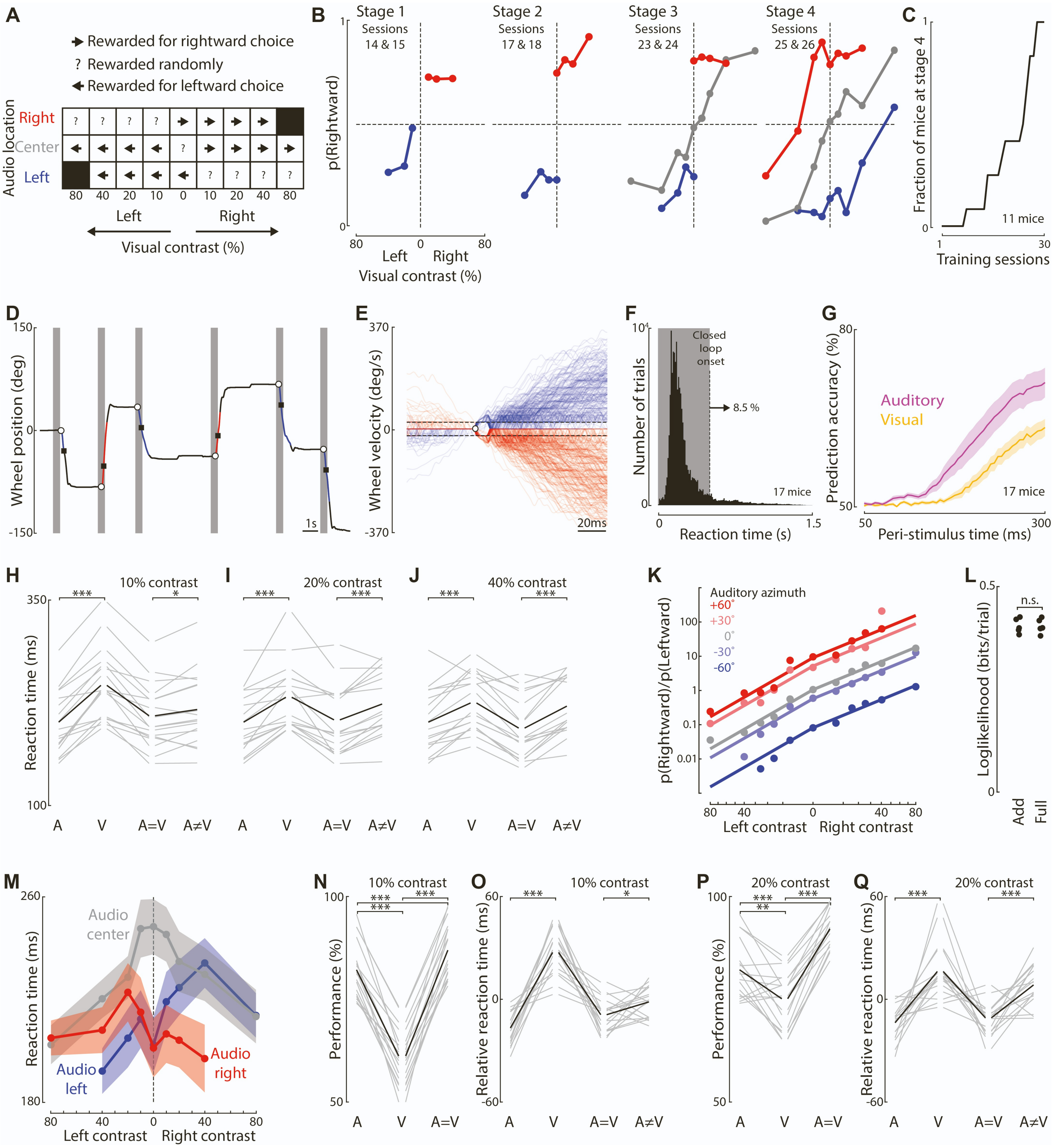

Figure S1 Training and behavioral classification, related to Figure 1

***(A)** Matrix of stimulus conditions and their corresponding reward criteria. **(B)** Training progression for one mouse. Mice first reach proficiency with audiovisual coherent trials (Stage 1), before we introduce unisensory auditory (Stage 2), unisensory visual (stage 3) and conflict (Stage 4) stimulus types. Plots for stages 1, 2, and 3, were made from the final two sessions of these stages. For stage 4, data is taken from the mouse's first two sessions at that stage. **(C)** Number of sessions required to train mice. In our final training pipeline, ~80% of mice learned the task. Those that did learn required less than 30 sessions (median: 24.5). **(D)** Sample of wheel position trace. Grey regions indicate the 500ms open-loop period, beginning with stimulus onset. Open circles and squares indicate the movement onset and decision threshold (see STAR Methods). **(E)** Zoom in to wheel velocity traces around the time of movement onset (open circle), for the same behavioral session as (D). Red and blue lines indicate leftward and rightward choices. Dashed lines represent the velocity thresholds for movement onset (see STAR Methods). **(F)** Histogram of reaction times across 17 mice (~156K trials). Shaded region indicates the 500ms open-loop period, during which 91.5% of movements were initiated. **(G)** Accuracy of predicting whether a stimulus was presented on the left or right side from wheel velocity at each time point relative to stimulus onset, using a threshold obtained by minimizing an SVM loss function, for unisensory visual (yellow) or unisensory auditory (magenta) trials. Shaded areas indicate the standard error across 17 mice. Earlier predictions on auditory trials confirm that mice can decode the location of auditory stimuli earlier than visual stimuli (i.e. earlier auditory reaction times do not reflect guesses). **(H)** Comparing median reaction times for each stimulus type for each mouse, for trials with 10% visual contrast. Grey and black lines indicate individual mice and the mean across mice. ***: p*

< 0.001, $*$: $p < 0.05$ (17 mice, paired t-test); only comparisons between auditory and visual unisensory, and between conflict and coherent are shown. ***(I)*** *As in (H), but for trials with 20% visual contrast.* ***(J)*** *As in (H), but for trials with 40% visual contrast.* ***(K)*** *Fit of the additive model to a mouse presented with 5 different auditory locations and 11 different contrast levels. Two additional parameters were used to fit the additional auditory stimuli (see STAR Methods).* ***(L)*** *5-fold cross-validated estimates of the loglikelihood for the additive and full (a parameter for each stimulus combination) behavioral models relative to a bias-only model. n.s. $p > 0.05$* ***(M)*** *The mean (across 17 mice) of the median reaction times for each stimulus condition ($\sim$156K trials). Shading: standard error across mice.* ***(N)*** *Mouse performance (% rewarded trials) for unisensory auditory, unisensory visual, and coherent multisensory stimulus conditions (correct performance on conflict trials is undefined). This panel shows only trials with 10% visual contrast. Grey and black lines indicate individual mice and the mean across mice. $***$: $p < 0.001$ (17 mice, paired t-test).* ***(O)*** *As in (H), but for reaction times relative to the mouse's mean reaction time across all stimulus types.* ***(P)*** *As in (N), but for trials with 20% visual contrast. $***$: $p < 0.001$ (17 mice, paired t-test). Data with 40% contrast is shown in Figure 1E.* ***(Q)*** *As in (I), but with reaction times relative to the mouse's mean reaction time across all stimulus types. Data with 40% contrast is shown in Figure 1B.*

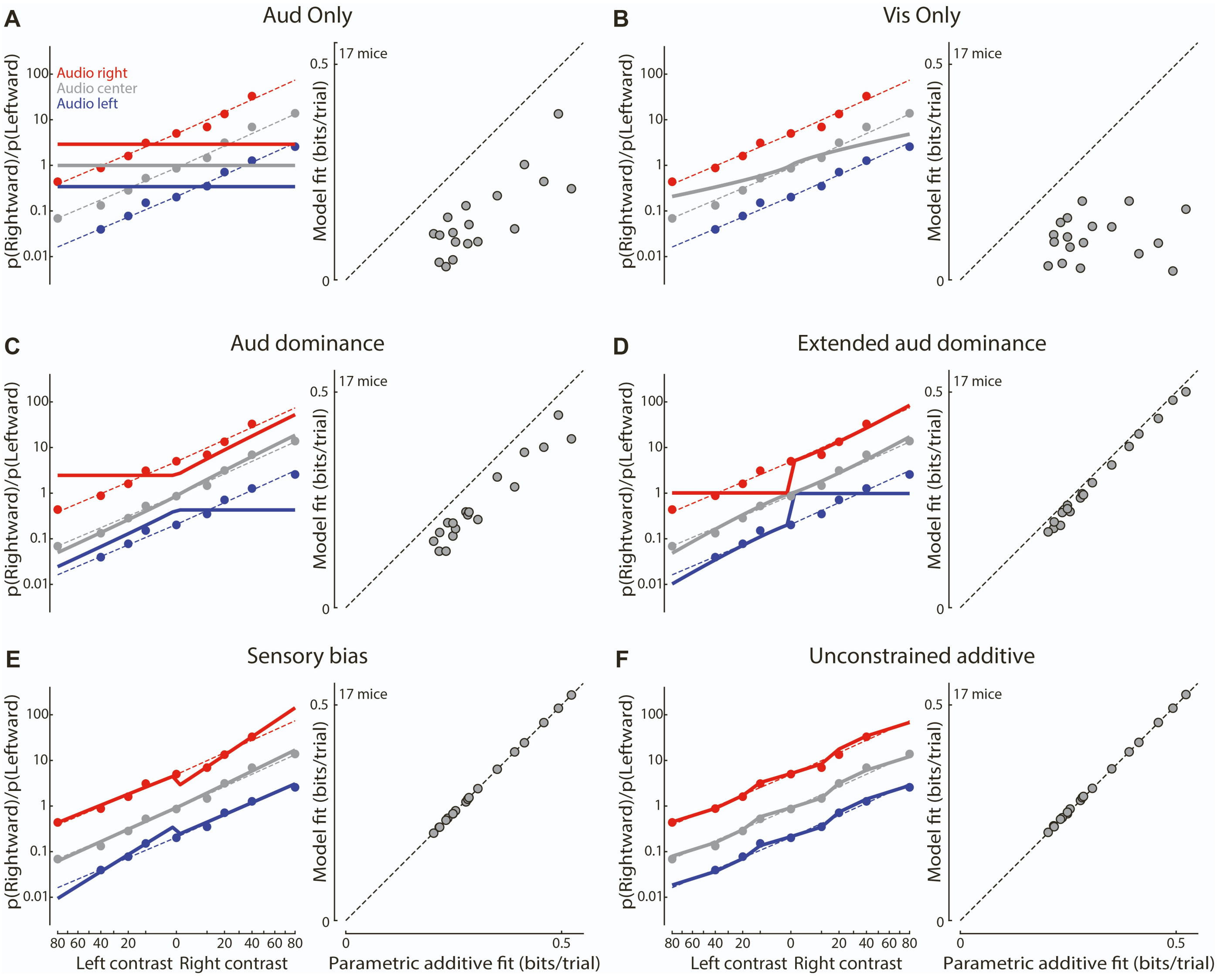

Figure S2 No evidence for sensory bias in behavior, related to Figure 1

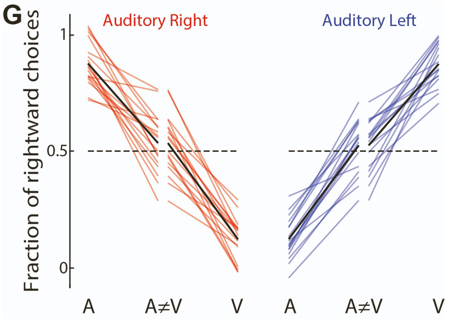

__(A) Left__: Fit of an auditory-only model where both visual sensitivities (v_R and v_L) are set to 0. Data plotted as the odds of choosing right vs. left (in log coordinates, Y-axis), as a function of visual contrast raised to the power γ. Closed circles: data combined across 17 mice. Red, grey, and blue curves: combined fit across all mice (17 mice ~156K trials). Dashed lines: fit of the additive model to the same data. The x-axis is visual contrast raised to the power γ from the additive model. __Right__: Log_2-likelihood ratio for the auditory-only model versus the parametric additive model (both models assessed by 5-fold cross-validation relative to a bias-only model). The parametric additive model is a significantly better fit ($p < 0.001$, paired t-test). __(B)__ As in (A), but for a visual-only model where both auditory sensitivities (a_R and a_L) are set to zero. The parametric additive model is a significantly better fit ($p < 0.001$). __(C)__ As in (A), but for a modified model that incorporates auditory dominance. Here, both visual sensitivities (v_R and v_L) are set to zero on audiovisual conflict trials. The parametric additive model is a significantly better fit ($p < 0.001$). __(D)__ As in (A) but for an extended auditory dominance model with an additional parameter to allow for a change in auditory sensitivity on conflict trials (see STAR Methods). The parametric additive model is a significantly better fit ($p < 0.001$). __(E)__ As in (A), but for a general sensory bias model with 4 additional parameters to allow for a change in visual or auditory sensitivity on conflict or coherent trials. The additive model is a subset of this model (see STAR Methods). There is no significant difference in fit quality between this model and the parametric additive model ($p > 0.05$). __(F)__ As in (A), but for the unconstrained additive model with a parameter for each auditory and visual stimulus condition. The superiority of the additive unconstrained model can be seen for example at visual contrast of +10, where the probability of a rightward choice is slightly below the parametric prediction for all three auditory conditions. The unconstrained additive model is a significantly better fit than the parametric additive model ($p < 0.01$). __(G)__ Fraction of rightward choices for auditory, visual, and conflict stimulus types at the "matched" visual contrast for each mouse. Red or blue indicate stimulus conditions where the auditory stimulus was on the right or left. The "matched" contrast is the contrast that produces ~(1-X) fraction of rightward choices, where X is the fraction of rightward choices in the paired auditory stimulus condition. For both left and right auditory stimuli, the fraction of rightward choices on conflict trials is not significantly different from 0.5 ($p > 0.05$, t-test, $n = 17$ mice).

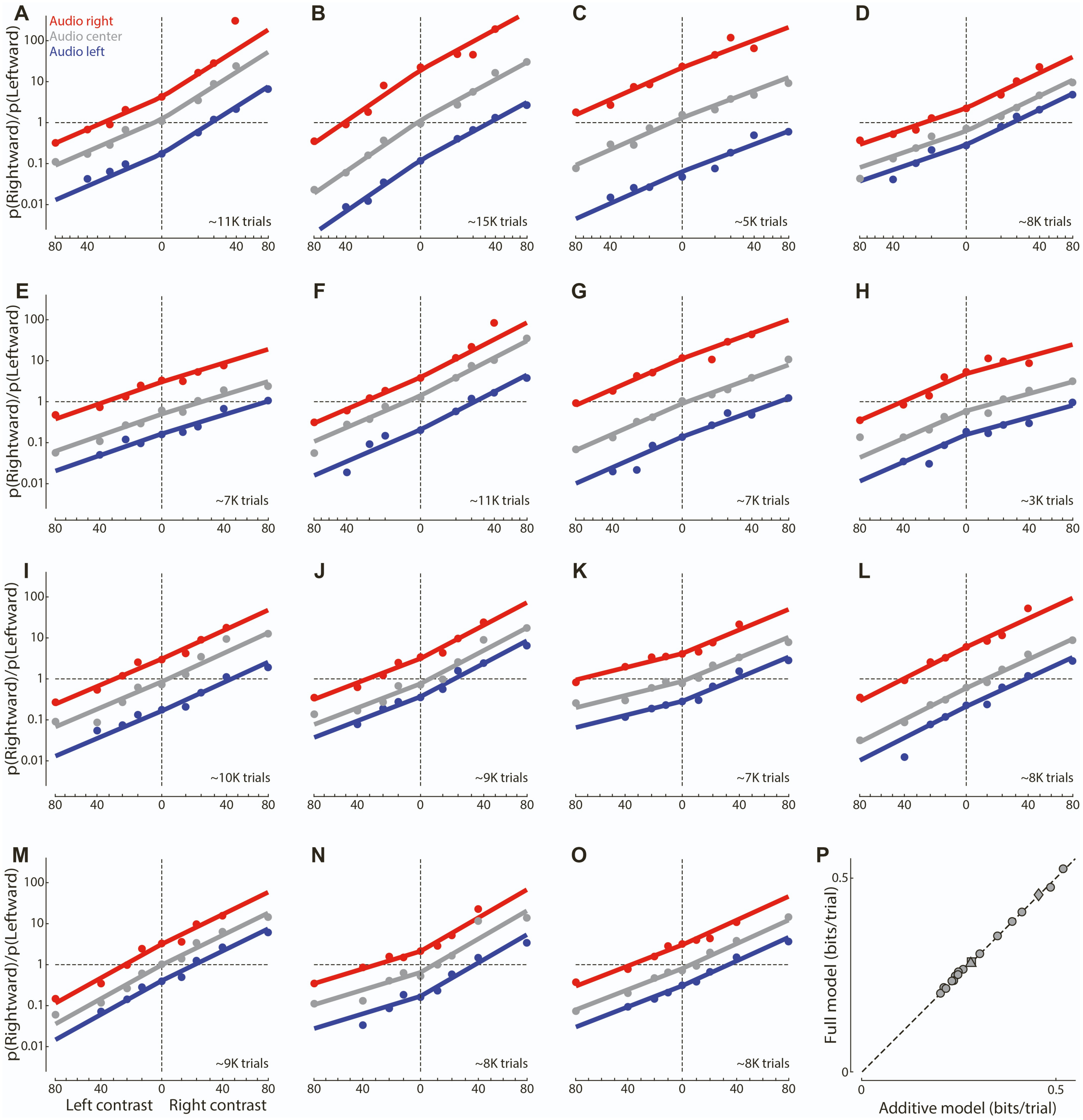

Figure S3 Additive model performance for each mouse, related to Figure 1

***(A-O)** Fits of the additive model to all 15 mice additional to those shown in Figure 1C, with different auditory and visual proficiencies, plotted as in Figure 1F. **(P)** Cross-validated loglikelihood ratio (relative to a bias-only model) for the full model versus the additive model trained on only unisensory stimulus conditions, plotted as in Figure 1H. There is no significant difference between models. p > 0.05 (17 mice, paired t-test).*

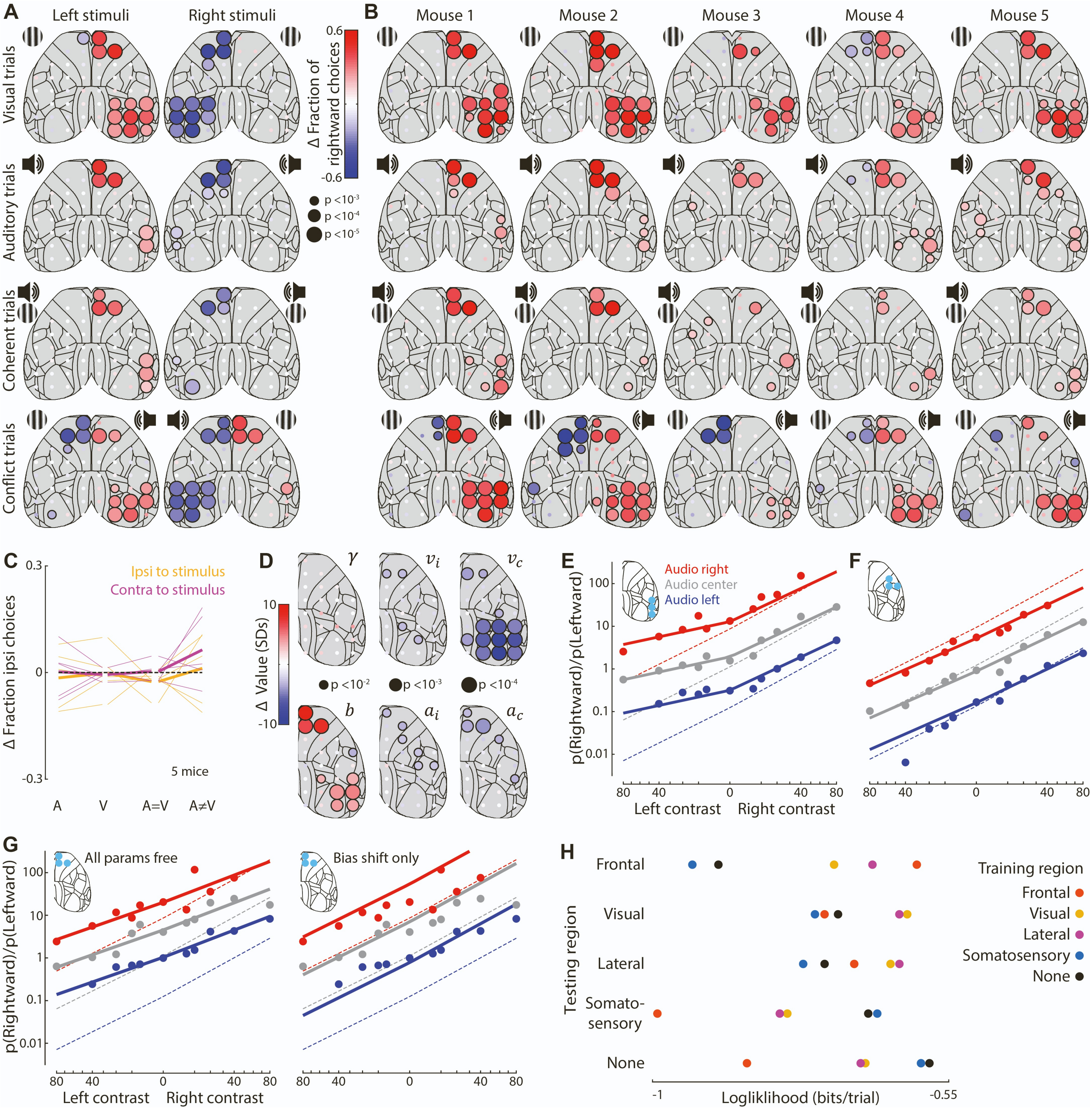

Figure S4 Further analysis of optogenetic inactivation effects on choice, model, and brain regions, related to Figure 2

***(A)** Change in the fraction of rightward choices for 52 stimulation sites for unisensory visual, unisensory auditory, coherent, and conflict stimulus conditions. The position of the grating and loudspeaker symbol indicate the locations of the visual and auditory stimuli in each case; Left and right columns represent mirrored cases of the same stimulus condition. Dot color indicates change in the fraction of rightward choices and dot size represents statistical significance (5 mice, shuffle test, see STAR Methods). **(B)** As in (A), but each column represents an individual mouse, and mirrored trials were combined, reversing if necessary, so that the stimulus (visual in conflict trials) was always on the left. Dot color indicates change in the fraction of rightward choices. **(C)** Change in the fraction of ipsilateral choices on trials when inactivation target was outside the brain (on the dental cement and/or skin of the mouse). No significant effect was observed for any stimulus condition for ipsilateral (gold) or contralateral (magenta) inactivation sites. $p > 0.05$ (paired t-test). **(D)** As in main text Figure 2F, but also allowing the contrast gain saturation parameter γ to be fit. No significant changes in the contrast gain parameter (γ) were observed (5 mice, shuffle test, see STAR Methods). **(E)** Fit of additive model to trials in which any of 3 sites in lateral sensory cortex were inactivated (5 mice, 6600 trials), plotted as in Figure 2G-H. Trials with inactivation of left lateral sensory cortex were included in the average after reflection. The deviation in model parameters was significant (paired t-test, $p < 0.05$). **(F)** As in (E), but for trials when somatosensory cortex was inactivated (5 mice, 6689 trials). The deviation in model parameters was significant ($p < 0.05$). **(G)** Fit of the additive model to right frontal cortex inactivation trials (5 mice, 5612 trials), with all parameters free (left, as in Figure 2H, γ was fixed) or with only the bias parameter free to change from its non-inactivation value (right). This "Bias shift only" approach resulted in a worse fit (paired t-test, $p < 0.05$), due to its inability to model changes in sensory sensitivity. **(H)** Loglikelihood for trials when each testing region was inactivated, evaluated using model parameters from a fit to trials in which the training region was inactivated. In each case, loglikelihood is highest when testing and training regions are the same. Every inter-region loglikelihood is significantly worse ($p < 0.05$, shuffle test, Bonferroni-corrected, see STAR Methods).*

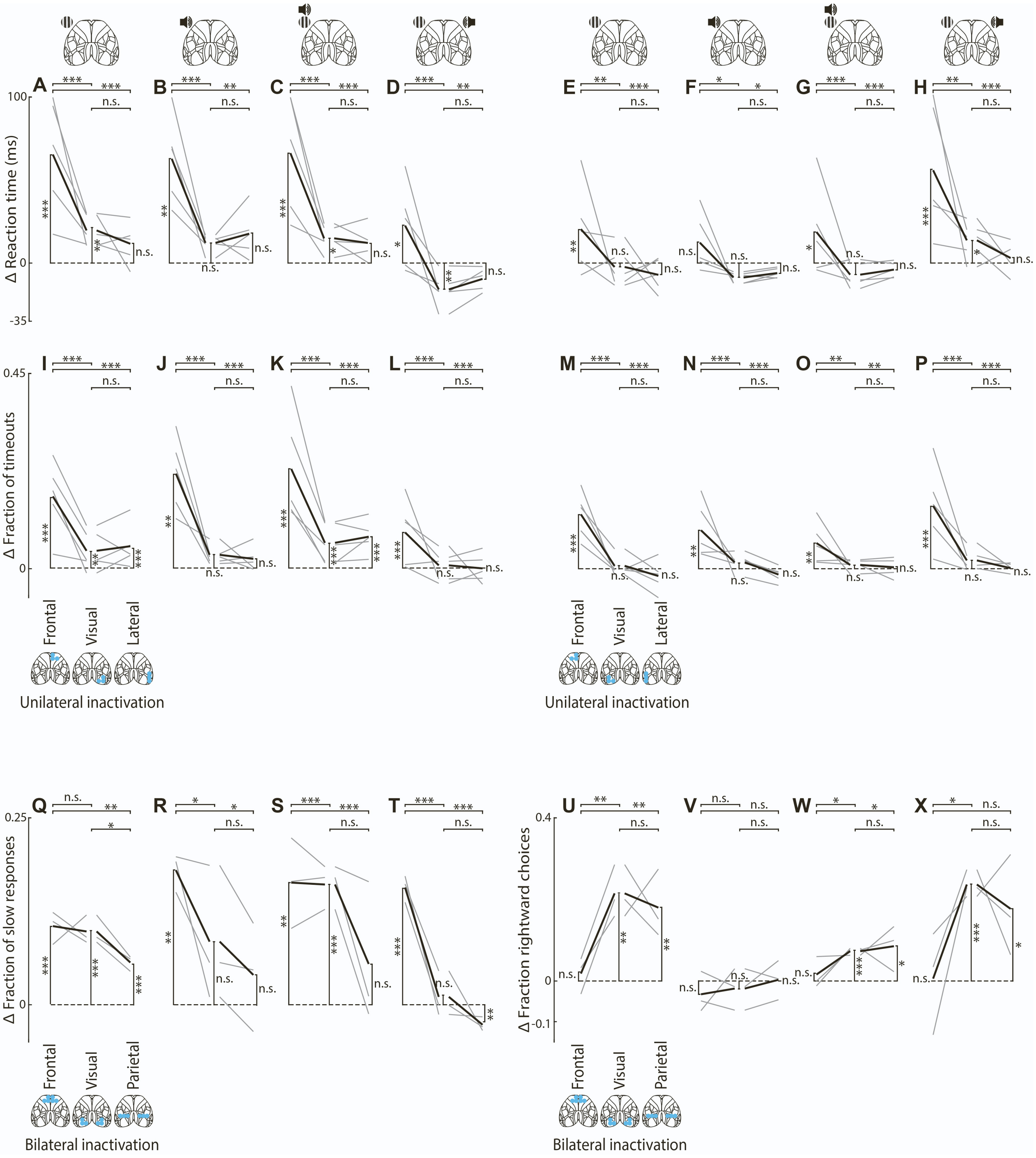

Figure S5 Further effects of uni- and bilateral inactivation on behavior, related to Figure 2

***(A)** Change in reaction times when frontal, visual, and lateral sensory cortex were inactivated (left, center and right) on visual trials, relative to non-inactivated trials with the same (contrast-matched) stimuli. Inactivated regions could be on the left or right, but were always contralateral to the visual stimulus. Grey and black lines indicate individual mice (n = 5) and the mean across mice. The values for each mouse were calculated by first computing the median reaction time for each contrast, then taking the mean of across contrasts. Values above 100 ms were truncated to 100 ms for visualization but not analyses. n.s: p > 0.05, ∗: p < 0.05, ∗∗: p < 0.01, ∗∗∗: p < 0.001 (linear mixed effects model). **(B)** As in (A) but for auditory trials. **(C)** As in (A) but for coherent trials. **(D)** As in (A), but for conflict trials where the visual stimulus was contralateral to the inactivated region. **(E-H)** As in (A-D) but for trials of reversed laterality such that the stimulus (visual in the case of conflict trials) was ipsilateral to the inactivated region. **(I-P)** As in (A-H) but for the change in the fraction of timeout trials (no response within 1.5s) rather than reaction times. **(Q-T)** As in (A-D) but for bilateral inactivation of frontal, visual, and parietal cortices (n = 3 mice). Here, a "slow response" is defined as any response time longer than 300 ms. We used this binarized metric to increase statistical power in view of the limited number of trials in the bilateral, compared to the unilateral, inactivation experiments. **(U-X)** As in (Q-T) but for the change in the fraction of rightward choices.*

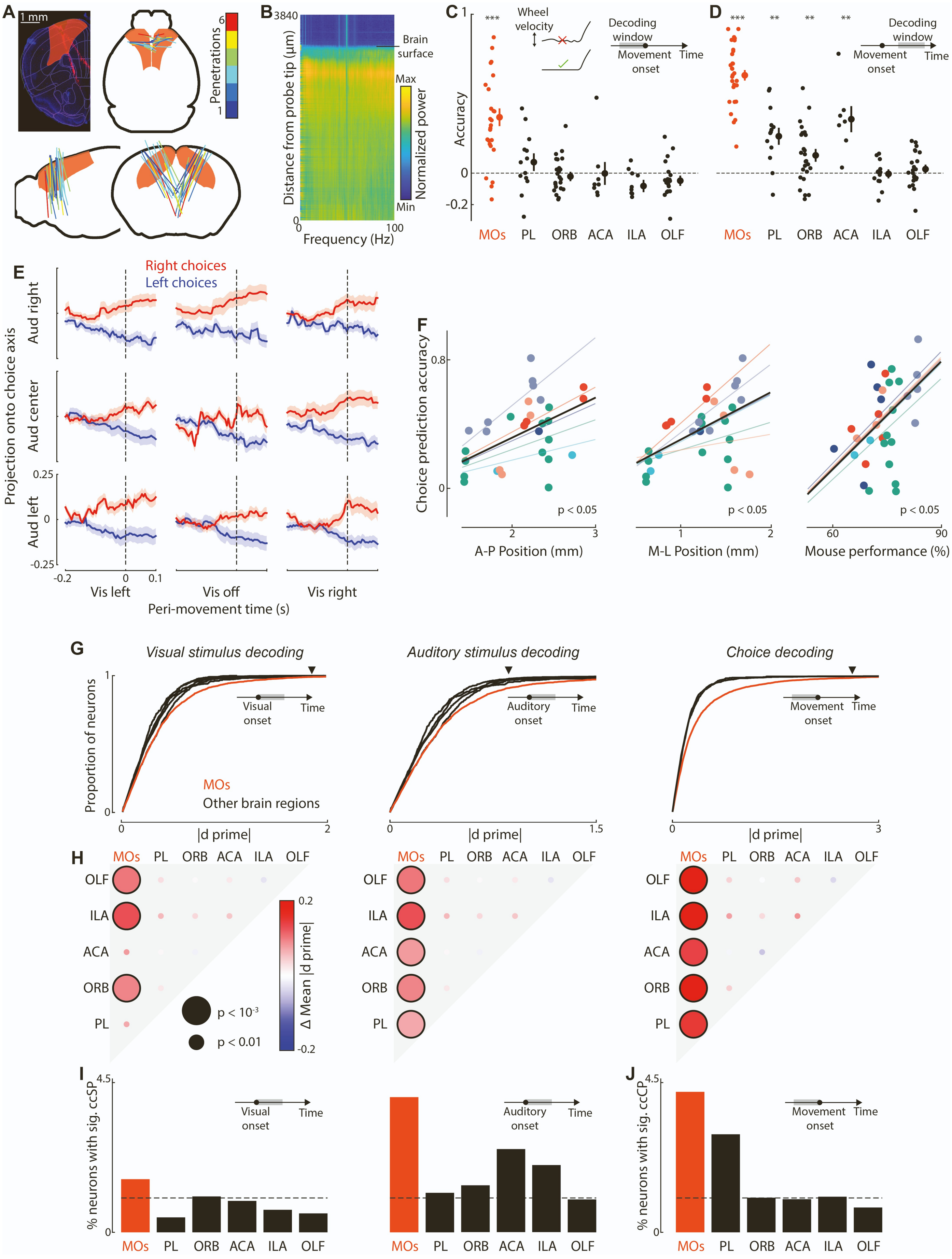

A
1 mm
Penetrations
6
1
B
3840
Distance from probe tip (μm)
Brain surface
Max
Normalized power
Min
0
0
100
Frequency (Hz)
C
1
Accuracy
0
-0.2
***
Wheel velocity
Decoding window
Movement onset
Time
MOs PL ORB ACA ILA OLF
D
*** ** ** **
Decoding window
Movement onset
Time
MOs PL ORB ACA ILA OLF
E
Right choices
Left choices
Projection onto choice axis
Aud right
Aud center
Aud left
0.25
0
-0.25
-0.2 0 0.1
Vis left
Vis off
Vis right
Peri-movement time (s)
F
Choice prediction accuracy
0.8
0
p < 0.05
2 3
A-P Position (mm)
1 2
M-L Position (mm)
60 90
Mouse performance (%)
G
Visual stimulus decoding
Auditory stimulus decoding
Choice decoding
Proportion of neurons
1
0
MOs
Other brain regions
|d prime|
0 2
0 1.5
0 3
Visual onset
Auditory onset
Movement onset
Time
H
MOs PL ORB ACA ILA OLF
OLF
ILA
ACA
ORB
PL
p < 10-3
p < 0.01
0.2
Δ Mean |d prime|
-0.2
I
4.5
% neurons with sig. ccSP
0
Visual onset
Auditory onset
Time
MOs PL ORB ACA ILA OLF
J
4.5
% neurons with sig. ccCP
0
Movement onset
Time
MOs PL ORB ACA ILA OLF

Figure S6 Electrophysiology methods and controls, related to Figure 3

(A) *Histological reconstruction of electrode tracks. Top left: Example coronal section (DAPI-staining, blue) at 1.86mm anterior to bregma with tracks from 2 insertions. Slices were registered to the Allen Atlas (Wang et al., 2020) (overlaid, with MOs highlighted in orange) using custom software (see STAR Methods). Right and bottom: reconstructed trajectories of all recordings analyzed, projected onto coronal, sagittal, and horizontal planes. Color indicates the number of penetrations along a given trajectory (one DiI-labelled penetration, same angles and point of insertion).* ***(B)*** *Normalized power spectrum (log(power), z-scored across probe depth) versus depth along probe for a single penetration. The point where the probe enters the brain can be identified by a sudden increase in power.* ***(C)*** *Cross-validated accuracy (relative to a bias model, see STAR Methods) of an SVM decoder trained to predict rightward or leftward choices based on population spiking activity time-averaged over a time window 0 ms to 130 ms preceding movement. Trials where mice made sub-threshold movements prior to movement onset were excluded (illustrated in inset). Each point represents the decoding accuracy from neurons in one brain region (Secondary motor (MOs), orbitofrontal (ORB), anterior cingulate (ACA), prelimbic (PL), infralimbic (ILA), or Olfactory (OLF)), from a single experimental session. ∗∗∗: $p < 0.001$ (≥ 5 sessions from 2-5 mice for each region, one-sided t-test).* ***(D)*** *As in (C), but with a time window of 150 ms to 300 ms after movement onset (see STAR Methods). Trials where mice made sub-threshold movements were not excluded. ∗∗: $p < 0.01$, ∗∗∗: $p < 0.001$.* ***(E)*** *For each stimulus condition, the projection of the population vector at time relative to movement onset to the choice axis (see STAR Methods), with red indicating rightward choice trials and blue indicating leftward choice trials. Solid lines are the mean projection across trials, with the shaded area indicating +/-1 SEM.* ***(F)*** *The accuracy of decoders trained to decode the upcoming choice of the animal 0 – 130 ms before movement onset as a function of either probe position relative to bregma along the anterior-posterior (A-P) axis (left), medial-lateral (M-L) axis (center), or mouse performance (percentage of correct choices, right). Each dot indicates an experiment session and dots of the same color represent sessions from the same mouse. Faint lines show the line of best fit using a linear mixed effects model with a random effect of mouse identity (intercept and slope), and the solid black line shows the mean of the lines of best fit. We observed a significant effect of A-P/M-L probe location, and mouse performance ($p < 0.05$, $n = 5$ mice, linear mixed effects model). There was no significant effect of probe location on stimulus decoding during passive stimulus presentation (data not shown).* ***(G)*** *Cumulative values of the discriminability indices (absolute d-prime, see STAR Methods*) across sessions. *Lines represent MOs (orange) and all other recorded brain regions (black). Arrows indicate the absolute d-prime values for the example neurons in (Figure 3B-D).* ***(H)*** *Significance for inter-region comparison of d-prime values from (G). d-prime values in MOs were significantly greater than all other regions for encoding auditory location and upcoming choice, but not significantly greater than ILA or ACA when considering visual location (Linear mixed effects model, see STAR Methods).* ***(I)*** *Proportion of neurons sensitive to visual (left) or auditory (right) stimulus location, estimated with combined conditions stimulus probability analysis (ccSP; see STAR Methods), after controlling for the other stimulus and for choice, using neural activity time-averaged over a window 0 to 300 ms after stimulus onset. In both cases, MOs has the highest proportion of significant neurons (more than 700 neurons were recorded in each area). Dashed line indicates the percentage of neurons expected by chance.* ***(J)*** *As in (I), but using combined conditions choice probability (ccCP; see STAR Methods) to estimate the proportion of neurons sensitive to the upcoming choice, after controlling for both stimuli, using neural activity time-averaged over a window 0 to 130 ms before movement onset.*

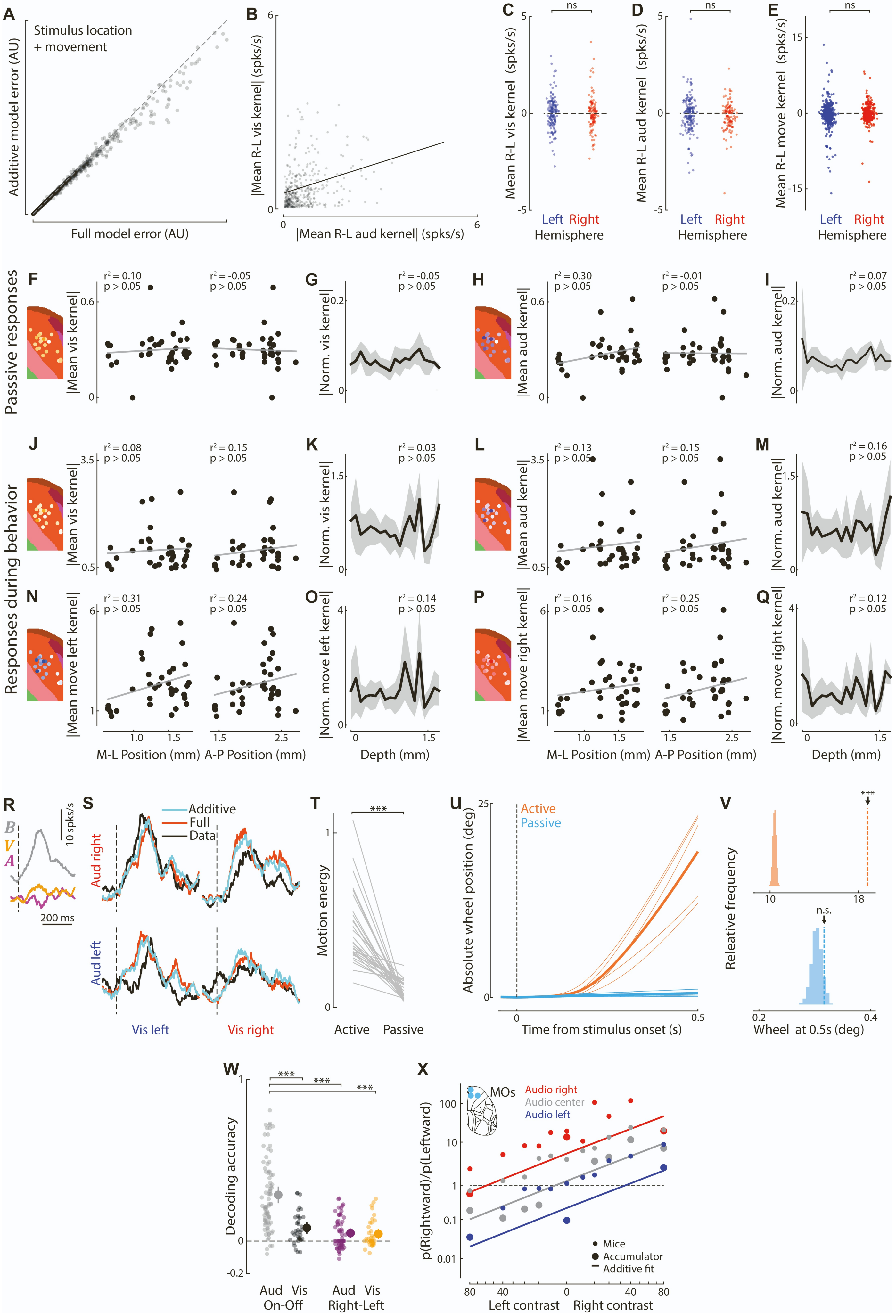
A
Stimulus location
+ movement
Additive model error (AU)
Full model error (AU)
B
|Mean R-L vis kernel| (spks/s)
|Mean R-L aud kernel| (spks/s)
C
Mean R-L vis kernel (spks/s)
D
Mean R-L aud kernel (spks/s)
E
Mean R-L move kernel (spks/s)
ns
Left Right
Hemisphere
Passive responses
Responses during behavior
F G H I J K L M N O P Q
|Mean vis kernel|
|Norm. vis kernel|
|Mean aud kernel|
|Norm. aud kernel|
|Mean move left kernel|
|Norm. move left kernel|
|Mean move right kernel|
|Norm. move right kernel|
M-L Position (mm)
A-P Position (mm)
Depth (mm)
R
S
T
U
V
B
V
A
10 spks/s
200 ms
Additive
Full
Data
Aud right
Aud left
Vis left
Vis right
Motion energy
Active
Passive
Absolute wheel position (deg)
Time from stimulus onset (s)
Relative frequency
Wheel at 0.5s (deg)
n.s.
W
X
Decoding accuracy
Aud Vis
On-Off
Aud Vis
Right-Left
MOs
Audio right
Audio center
Audio left
p(Rightward)/p(Leftward)
Mice
Accumulator
Additive fit
Left contrast
Right contrast

Figure S7 Additional analysis for neural recordings, related to Figure 4 and Figure 6

(A) *Prediction error for each neuron for the additive and full sensory-movement models (see STAR Methods). The full model has significantly higher error. (p < 0.01, 2183 cells, linear mixed-effects model). For visualization purposes, the largest 1% of errors were excluded from the plot, but not from statistical analysis.* ***(B)*** *Absolute value of time-averaged visual R-L kernel (V), versus absolute value of time-averaged auditory R-L kernel (A), after fitting the additive model under passive conditions for significant neurons. A time window from 0 to 300 ms after stimulus onset was used in both cases. Absolute responses are correlated, suggesting that if a neuron is predictable from spatial stimuli in one modality, it is more likely to be predictable from the other modality ($p < 10^{-5}$, 2509 cells, linear mixed-effects model).* ***(C)*** *Signed time average of the visual R-L kernel (V, Eqn 5 0 ms to 300 ms after stimulus onset) versus recorded hemisphere, after fitting the additive neural model ($N = 0$) under passive conditions ($D, M = 0$) (see STAR Methods). There is no significant lateralization in spatial preference (p > 0.05; 273 cells, linear mixed-effects model).* ***(D)*** *As in (C), but for the auditory R-L kernel (A). (p > 0.05; 287 cells).* ***(E)*** *As in (C), but the movement R-L kernel (D, -200 to 400 ms relative to movement onset), after fitting the additive model during active behavior (p > 0.05; 784 cells).* ***(F)*** *Left: each dot represents the mean position of MOs neurons located in a single probe. Color saturation indicates the magnitude of the mean absolute visual kernel size in neurons recorded from each probe during passive conditions. Right: the relationship between the mean absolute visual kernel of neurons in each probe and their medial-lateral position and anterior-posterior position (n = 35 penetrations, Pearson's correlation test).* ***(G)*** *Relationship between the mean absolute visual kernel size and depth from the brain surface in a model fitted to normalized firing rates (see STAR Methods). Black lines and grey shading indicated the median and ± 1 median absolute deviation across probes (n = 35 penetrations, Pearson's correlation test).* ***(H-I)*** *Same as (F-G) but for auditory kernel size.* ***(J-K)*** *Same as (F-G) but for neural activity recorded during the behavioral task (n = 36 penetrations).* ***(L-M)*** *Same as (J-K) but for auditory kernel size.* ***(N-O)*** *Same as (J-K) but for movement-left kernel size.* ***(P-Q)*** *Same as (J-K) but for movement-right kernel size.* ***(R)*** *Example sensory kernels from fitting the additive neural model to a single neuron under passive conditions. The selected neuron has opposing sensitivities for auditory (left-preference) and visual (right-preference) stimulus locations. B: baseline kernel; V: visual direction kernel; A: auditory direction kernel.* ***(S)*** *Cross-validated fit using the model kernels from (R) to neural responses under passive conditions for all audiovisual combinations. Cyan and orange lines show predictions of the additive and full models, black line shows test-set average responses.* ***(T)*** *Difference in facial video motion energy (see STAR Methods) when mice are performing the behavior (active) versus passive presentation of stimuli (passive). Mice exhibit significantly less motion under passive conditions. $***$: p < 0.001 (30 sessions, paired t-test).* ***(U)*** *Absolute wheel position, relative to position at stimulus onset, during active behavior (orange) and passive stimulus presentation (blue). Thin lines represent the average of all trials for individual mice (n = 5), thick lines represent the mean across mice. Dashed line indicates stimulus onset time.* ***(V)*** *To statistically test for increased wheel movement following stimulus onset, we used a randomization method. Dashed vertical lines indicate mean absolute wheel position 0.5 s after stimulus onset during active behavior (top) and passive stimulus presentation (bottom), averaged across mice. Histograms show the same statistic computed from 1000 "pseudosessions" where stimulus onset times were randomized (see STAR Methods). Wheel displacement was significantly larger than the shuffled distribution for active behavior ($***$: p < 0.001, shuffle test) but not during passive presentation (p > 0.05, shuffle test).* ***(W)*** *Neurons were tested individually to see if they encoded the presence or location of visual and auditory stimuli (see STAR Methods). The plot shows the single-neuron decoding accuracy of all significant neurons for each discrimination. Neurons encoding auditory stimulus presence (grey, 82 cells) have higher decoding accuracy than all other categories (36/59/36 cells for black/ magenta/gold). $***$: p < 0.001 (t-test).* ***(X)*** *Mean behavior of the accumulator model with the activity of all neurons in the right hemisphere reduced by 60% to simulate inactivation of right MOs (large circles), plotted with the mean behavior from MOs-inactivated mice (5 mice, small circles, cf. Figure 2H). Solid lines represent the fit of the additive model to the accumulator model output. This model cannot capture the rightward choice bias following right MOs inactivation because MOs neurons preferring either direction of stimulus are found equally in both hemispheres.*

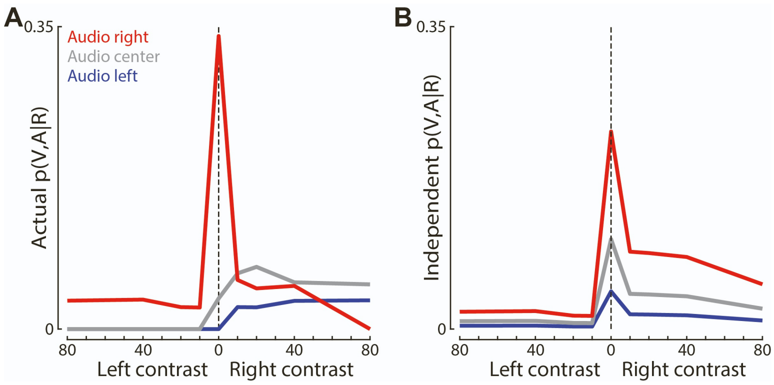

Figure S8 Conditional independence holds only approximately in our task, related to STAR Methods

(A) *The actual value of* $p(V, A \mid R)$ *for each contrast when the auditory stimulus was presented on the right/center/left (red/grey/blue). Values were computed by Bayes' theorem* $p(V, A \mid R) = p(R|V, A)p(V, A)/p(R)$, *summing* $p(V, A)$ *over ~156K trials from 17 mice.* ***(B)*** *As in (A), but for the conditional independence model* $p(V \mid R)p(A, \mid R)$.
